# RNA variant assessment using transactivation and transdifferentiation

**DOI:** 10.1016/j.ajhg.2024.06.018

**Published:** 2024-07-30

**Authors:** Emmylou C. Nicolas-Martinez, Olivia Robinson, Christian Pflueger, Alison Gardner, Mark A. Corbett, Tarin Ritchie, Thessa Kroes, Clare L. van Eyk, Ingrid E. Scheffer, Michael S. Hildebrand, Jean-Vianney Barnier, Véronique Rousseau, David Genevieve, Virginie Haushalter, Amélie Piton, Anne-Sophie Denommé-Pichon, Ange-Line Bruel, Sophie Nambot, Bertrand Isidor, John Grigg, Tina Gonzalez, Sondhya Ghedia, Rhett G. Marchant, Adam Bournazos, Wui-Kwan Wong, Richard I. Webster, Frances J. Evesson, Kristi J. Jones, PERSYST Investigator Team, Dimitar N. Azmanov, Christopher P. Barnett, Simon C. Barry, Gareth Baynam, Samuel F. Berkovic, John Christodoulou, David J. Coman, Sandra Cooper, Mark A. Corbett, Martin Delatycki, Tracy E. Dudding, Sue Fletcher, Alison E. Gardner, Jozef Gecz, Megan J. Higgins, Michael S. Hildebrand, Lachlan A. Jolly, Ryan Lister, Julie McGaughran, Christian Pflueger, Cathryn Poulton, Tony Roscioli, Ingrid Scheffer Hamish S. Scott, Andrew H. Sinclair, Amanda B. Spurdle, Tiong Y. Tan, Clare L. van Eyk, Irina Voineagu, Sandra T. Cooper, Ryan Lister, Jozef Gecz, Lachlan A. Jolly

**Affiliations:** 1 The Robinson Research Institute, University of Adelaide, Adelaide, SA 5005, Australia; 2 School of Biomedicine, University of Adelaide, Adelaide, SA 5005, Australia; 3 Harry Perkins Institute of Medical Research, Nedlands, WA 6009, Australia; 4 Australian Research Council Centre of Excellence in Plant Energy Biology, School of Molecular Sciences, The University of Western Australia, Crawley, WA 6009, Australia; 5 Adelaide Medical School, University of Adelaide, Adelaide, SA 5005, Australia; 6 Epilepsy Research Centre, Department of Medicine, The University of Melbourne, Austin Health, Heidelberg, VIC 3084, Australia; 7 Murdoch Children’s Research Institute, Parkville, VIC 3052, Australia; 8 Florey Institute of Neuroscience and Mental Health, University of Melbourne, Parkville, VIC 3052, Australia; 9 Department of Paediatrics, University of Melbourne, Royal Children’s Hospital, Parkville, VIC 3052, Australia; 10 Institut des Neurosciences Paris-Saclay, UMR 9197, CNRS, Université Paris-Saclay, Saclay, France; 11 Montpellier University, Inserm U1183, Reference Center for Rare Diseases Developmental Anomaly and Malformative Syndromes, Genetics Department, Montpellier Hospital, Montpellier, France; 12 Genetic Diagnosis Laboratory, Strasbourg University Hospital, Strasbourg, France; 13 CRMRs "Anomalies du Développement et syndromes malformatifs" et "Déficiences Intellectuelles de causes rares", Centre de Génétique, CHU Dijon, Dijon, France; 14 INSERM UMR1231, GAD "Génétique des Anomalies du Développement," FHU-TRANSLAD, University of Burgundy, Dijon, France; 15 Speciality of Ophthalmology, Save Sight Institute, Faculty of Medicine and Health, The University of Sydney, Sydney, NSW 2000, Australia; 16 Department of Clinical Genetics, Royal North Shore Hospital, St Leonards, NSW 2065, Australia; 17 Kids Neuroscience Centre, Kids Research, Children’s Hospital at Westmead, Westmead, NSW 2145, Australia; 18 Faculty of Medicine and Health, The University of Sydney, Sydney, NSW 2000, Australia; 19 Children’s Medical Research Institute, Westmead, NSW 2145, Australia; 20 Department of Paediatric Neurology, Children’s Hospital at Westmead, Sydney, NSW 2000, Australia; 21 Department of Clinical Genetics, Children’s Hospital at Westmead, Sydney, NSW 2000, Australia; 22 South Australian Health and Medical Research Institute, Adelaide, SA 5000, Australia

## Abstract

Understanding the impact of splicing and nonsense variants on RNA is crucial for the resolution of variant classification as well as their suitability for precision medicine interventions. This is primarily enabled through RNA studies involving transcriptomics followed by targeted assays using RNA isolated from clinically accessible tissues (CATs) such as blood or skin of affected individuals. Insufficient disease gene expression in CATs does however pose a major barrier to RNA based investigations, which we show is relevant to 1,436 Mendelian disease genes. We term these “silent” Mendelian genes (SMGs), the largest portion (36%) of which are associated with neurological disorders. We developed two approaches to induce SMG expression in human dermal fibroblasts (HDFs) to overcome this limitation, including CRISPR-activation-based gene transactivation and fibroblast-to-neuron transdifferentiation. Initial transactivation screens involving 40 SMGs stimulated our development of a highly multiplexed transactivation system culminating in the 6- to 90,000-fold induction of expression of 20/20 (100%) SMGs tested in HDFs. Transdifferentiation of HDFs directly to neurons led to expression of 193/516 (37.4%) of SMGs implicated in neurological disease. The magnitude and isoform diversity of SMG expression following either transactivation or transdifferentiation was comparable to clinically relevant tissues. We apply transdifferentiation and/or gene transactivation combined with short- and long-read RNA sequencing to investigate the impact that variants in *USH2A*, *SCN1A*, *DMD*, and *PAK3* have on RNA using HDFs derived from affected individuals. Transactivation and transdifferentiation represent rapid, scalable functional genomic solutions to investigate variants impacting SMGs in the patient cell and genomic context.

## Graphical abstract

**Figure:**
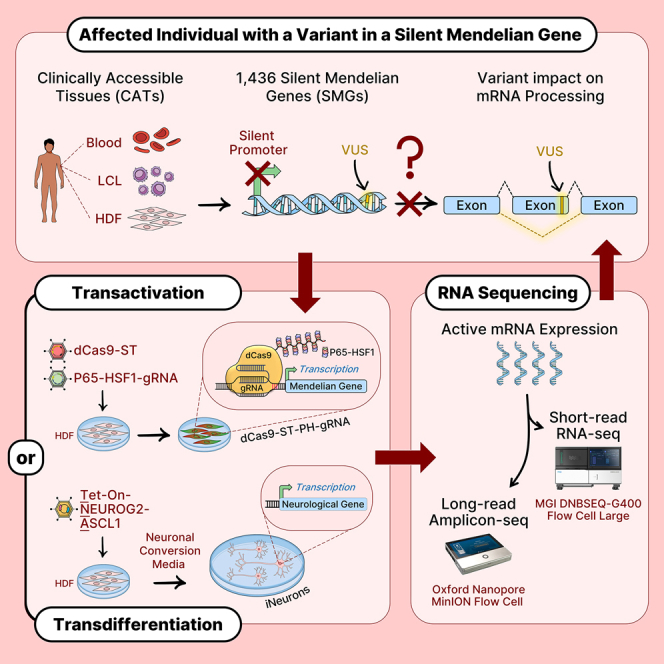

## Teaser

Silent or insufficient disease gene expression in clinically accessible tissues is a major barrier to RNA-based investigations of variant impact. This study overcomes this limitation by applying either gene transactivation or neuronal transdifferentiation to obtain RNA of silent disease genes for variant assessment utilizing dermal fibroblasts from affected individuals.

## Introduction

The expanding catalog of Mendelian disease genes is accompanied by exponential growth in the number of variants of uncertain significance (VUSs), which remain challenging to resolve and continue to accumulate.^1^ While aggregation of genomic data from healthy and affected individuals may resolve a portion of VUSs through sequence reanalysis, many current and future VUSs will remain orphan findings, requiring additional evidence to resolve their effect. The American College of Medical Genetics and Genomics and the Association for Molecular Pathology (ACMG-AMP) provide guidelines for the interpretation of DNA variant pathogenicity and state that a well-established functional assay demonstrating the deleterious impact of the variant on gene activity is considered strong evidence for pathogenicity.^2^^,^^3^ Studies conducted in the context of an affected individual’s genetic background are recommended due to the possible influence of variant effect modifiers. These include variants in *cis* or *trans* that impact expression or splice quantitative trait loci,^4^ contribute to genetic risk or susceptibility,^5^ or that influence important mechanisms such as nonsense mediated mRNA decay (NMD),^6^ genetic compensation,^7^ allele-biased expression,^8^ and X inactivation^9^ among others. As such, patient blood or skin samples/cell lines are often desired for these investigations to enable direct and unequivocal assessment of variant impacts. For DNA variants predicted to alter RNA processing, functional studies that determine empirically and exactly how RNA splicing patterns are altered and/or whether aberrant transcript(s) are susceptible to NMD, is often vital for clinical interpretation.^10^^,^^11^^,^^12^ Such studies are critical as variants suspected to alter RNA splicing or abundance can be difficult to interpret from DNA sequence alone and are also frequent. Variants that affect pre-mRNA splicing account for at least 13% of disease-causing variants and are likely underestimated due to the ascertainment bias toward coding variants,^10^^,^^13^ while variants predicted to result in a premature termination codon (PTC) and potentially eliciting downstream NMD represent an estimated 30% of all disease-causing variants.^14^ Establishing splice altering or a PTC variant effect is important for at least two major reasons. Firstly, it is crucial for accurate classification of these variants as pathogenic or benign, and secondly, it is essential to understand the actual variant effect on RNA and hence its precise assessment for current or future therapies (e.g., anti-sense oligonucleotide or nonsense suppression approaches).^15^^,^^16^^,^^17^ Consequently, even variants classified as pathogenic based on DNA sequence alone benefit from functional studies to address variant mechanism in view of personalized genomic medicine.^15^

Splice altering variants can lead to diverse molecular outcomes including cryptic splicing, exon skipping, intron inclusion, leaky splicing, or the introduction of pseudo-exons.^18^ It is accepted that variants in the canonical ±1 or ±2 splice sites are pathogenic if found in genes where loss of function is an established disease mechanism.^3^ Non-canonical splice variants affecting extended donor and acceptor sites as well as distal intronic and exonic pre-mRNA features are far more challenging to interpret and there is a growing bottleneck in their resolution.^18^^,^^19^^,^^20^^,^^21^^,^^22^ Predictive algorithms are evolving for the prioritization of splicing variants, including the latest generation of machine learning methods such as SpliceAI and more recently SpliceVault.^23^^,^^24^^,^^25^^,^^26^^,^^27^^,^^28^ Yet most non-canonical splice altering variants remain classified as VUSs because predictive evidence remains insufficient alone to re-classify them as pathogenic in clinical settings.^2^^,^^3^ In contrast, nonsense or frameshift variants are frequently assessed as loss-of-function and, as such, pathogenic based on DNA sequence alone, with NMD of such mRNAs assumed, albeit infrequently assessed experimentally. While such loss of function mechanism may also be supported by clinical or other evidence, in other cases it is less clear, and the role of NMD should be questioned given the many reported examples where mRNAs containing NMD-compliant PTCs fully or partially escape NMD, leading to unexpected mechanisms of disease.^29^^,^^30^^,^^31^^,^^32^^,^^33^^,^^34^^,^^35^^,^^36^^,^^37^

The ACMG guidelines state that functional investigation of variants through RNA analysis can garner strong evidence if assays are well established, reproducible, robust, and conducted in the context of the affected individual’s biological environment and genetic background.^2^^,^^3^^,^^11^ Functional RNA investigations are shown to result in the reclassification of 75% of putative splicing variants,^11^ while RNA-sequencing (RNA-seq)-based expression outlier analysis also increases diagnostic yield significantly.^38^^,^^39^^,^^40^^,^^41^ RNA-seq has now emerged as the first-tier approach to resolve mRNA altering variants,^12^^,^^39^^,^^42^^,^^43^ an approach that also addresses the variants mechanism of action, as it reveals the full spectrum of aberrant splicing outcomes, and involvement of NMD, and therefore may inform treatment options, i.e., “variant treatability.”^15^^,^^16^^,^^17^ Despite these benefits, long-standing challenges remain if the variant requiring RNA-based assessment is in a gene that is not sufficiently expressed in clinically accessible tissues (CATs) of blood and skin: how can RNA be functionally assessed if the expression of the corresponding gene or gene isoform is silent in CATs? Access to a biopsy from the clinically relevant tissue (CRTs) may be an option (e.g., muscle biopsy), but the risks are often too high (e.g., when considering CRTs like brain or liver) or collection of CRTs is not practically possible (e.g., fetal tissue from an adult individual or specific rare tissues). RNA analysis using high cycle and/or nested PCR or ultradeep RNA-seq (>billion reads/sample) are options for some lowly expressed genes but might be inherently biased in view of the PCR amplicon(s) design or the mRNA isoform diversity inherent to the CAT.^11^^,^^44^ Other approaches include engineering of exogenous cDNAs or mini-gene expression constructs^45^^,^^46^ or introduction of variants (e.g., by CRISPR-Cas9) into the genomes of generic models, often cancerous cell lines, expressing the gene of interest.^47^ These techniques are, however, “variant centric,” involving extensive redesign of reagents on a per variant basis. Other relevant considerations and limitations of such assays can also include targeting only single or even partial gene isoforms, impact of episomal expression artifacts, and variant assessment in non-patient cell and genome context. Creation of patient induced pluripotent stem cells (iPSCs) followed by tissue-specific differentiation^48^ is another solution but requires proficiency in iPSC techniques and carries a large resource burden per variant. Collectively, while approaches with established utility exist to overcome the issue of lack of disease gene expression in CATs, they are non-trivial in terms of resources and expertise as well as their scalability for higher-throughput applications. Consequently, there is a major gap in our ability to assess variants impacting RNA in the context of an individual’s own genome in genes and isoforms that are not expressed in CATs. In this study, we address this challenge by developing broadly applicable gene transactivation and cell transdifferentiation approaches for variant effect assessment using human dermal fibroblasts (HDFs). We couple these techniques with short- and long-read RNA-seq to investigate and resolve the mechanism of action of variants in Mendelian disease genes that are not otherwise sufficiently expressed in CATs.

## Subjects, material, and methods

### Subjects

This study was approved by the Women’s and Children’s Health Network Human Research Ethics Committee, South Australia, Australia (HRE00188) and the Sydney Children’s Hospitals Network Human Research Ethics Committee (protocol 2019/ETH11736), and French Institutional Review Boards (Nantes, Dijon and Montpellier). All subject information and materials were provided following informed guardian consent. The individual with the *SCN1A* variant was recruited via the Epilepsy Research Centre, Department of Medicine, The University of Melbourne, Austin Health, Heidelberg, VIC, 3084, Australia. The individual with the *USH2A* variants was recruited via Specialty of Ophthalmology, Save Sight Institute, Faculty of Medicine and Health, The University of Sydney, Sydney, NSW, 2000, Australia. The individual with the *DMD* variant was recruited via the Kids Neuroscience Centre, Kids Research, Children’s Hospital at Westmead, Westmead, NSW, 2145, Australia. Two out of three individuals with *PAK3* variants were recruited using French National Genetics Network on Intellectual Disability and the DEFIDIAG infrastructure. The molecular *PAK3* analyses were performed through genetic diagnosis protocols using trio genome sequencing. The third individual with a *PAK3* variant was identified as part of a diagnostic process, using exome sequencing. One identified variant was submitted on the ClinVar database with the following accession number: SCV001736950.1 (g.110437602G>T [GenBank: NC_000023.10] [c.1066G>T (GenBank: NM_002578.5); p.Glu356Ter (GenBank: NP_002569.1)]).

### Recombinant DNA engineering

All plasmids were prepared using Endotoxin Free Maxi Prep Kits as per the manufacturer’s instructions (Qiagen, Hilden, Germany). For gene transactivation, the vector p.dCas9-ST-BFP was obtained from Addgene (#60903; Watertown, MA, USA). The P2A-Blue Fluorescent Protein (BFP) cassette was substituted for the P2A-mCherry cassette using *NotI* and *XbaI* restriction sites to generate p.dCas9-ST-mCherry (used for transient transductions). The P2A-mCherry cassette in p.dCas9-ST-mCherry vector was replaced with P2A-mCherry-T2A-Blasticidin Resistance cassette using *NotI* and *XhoI* restriction sites to create the p.dCas9-ST-mCherry-BSD vector (used for generating stable cell lines). The p.P65-HSF1 vector was kindly gifted by Ryan Lister (The University of Western Australia, Perth, Australia). The guide RNA (gRNA) cassettes containing a multiplex of four gRNAs targeting each gene were synthesized and packaged into pUC57 backbones commercially (GenScript, Nanjing, China) and then inserted into the p.P65-HSF1 vector using *EcoRI* and *KpnI* restriction sites. The gRNA pooled library was synthesized and cloned into pJR100 (Addgene: #187240) by Vector Builder (Chicago, IL, USA). For nuclease assays, the p.Cas9 vector was obtained from Addgene (#48138) and gRNAs against *AGAP1*, *GRM7*, and *PAK3* cloned in as previously described.^49^ Briefly, the forward and reverse 20 bp gRNA oligonucleotides for the three genes were synthesized commercially (GenScript) with additional nucleotide overhang sequences (forward oligo: 5′-*cacc*NN … NN-3’; reverse oligo: 5′-*aaac*NN … NN-3′). Oligonucleotide pairs were phosphorylated using T4 Polynucleotide Kinase (New England BioLabs) and annealed together using 10X T4 Ligation Buffer (New England BioLabs) following heat cycle: 5 min at 95°C and a ramp down to 25 °C at 5°C per min. The oligo duplexes were then ligated into the p.Cas9 backbone via the *BbsI* cloning site. The correct insertion of the guides was confirmed via Sanger sequencing (Australian Genome Research Facility) using the hU6 forward primer (Table S1). For HDF transdifferentiation, the p.TNA vector (i.e., pLVX-UbC-rtTA-Ngn2:2A:Ascl1) was obtained from Addgene (#127289).

### Generating lentiviral particles

Lentiviral particles were generated by Functional Genomics South Australia (FGSA, University of Adelaide, Adelaide, Australia) using methods as previously described.^50^ Briefly, human embryonic kidney 293T cells (#CRL-3216) were co-transfected with three plasmids: (1) the transfer vectors (either p.dCas9-ST, p.TNA, or p.P65-HSF1-gRNA vectors), (2) a packaging vector (psPAx2; Addgene #12260), and (3) a viral envelope vector (pMD2.G, Addgene: #12269), using Lipofectamine LTX and OPTI-MEM reagents as per the manufacturer’s protocol (Thermo Fisher Scientific, Waltham, MA, USA). Viral supernatants were collected at 24 h and 48 h time points post-transfection, passed through 0.45 μm filters, and concentrated by ultracentrifugation. Viral titers were determined by flow cytometry as previously described,^51^ typically producing 1 × 10^4^ – 1 × 10^6^ infective units/μL. Lentiviral particles were aliquoted and stored at −80°C.

### Cell culture

HEK293T cells and hTERT-immortalized foreskin fibroblast BJ-5ta (#CRL-4001) are from the American Type Culture Collection (Manassas, VA, USA). Control HDFs obtained from healthy individuals are from either Coriell Institute (lines GM02936 and GM05659; Camden, NJ, USA) or derived in house.^52^ HEK293T and HDFs were grown in Dulbecco’s modified Eagle medium (DMEM; Thermo Fisher Scientific) supplemented with 10% fetal bovine serum (FBS, CellSera, Rutherford, NSW, Australia) and 50 U/ml PenStrep (Thermo Fisher Scientific). BJ-5ta was cultured in growth media comprised of a 4:1 mixture of DMEM and Medium 199 (Thermo Fisher Scientific) supplemented with 10% FBS and 50 U/ml PenStrep. Cell cultures were kept in a humidified incubator maintained at 5% CO_2_ and 37°C. To deliver vector transgenes into HEK293T cells, Lipofectamine 3000 (Thermo Fisher Scientific) was used following the manufacturers 6-well format protocol. To deliver transgenes into HDFs and BJ-5ta, growth media specific for their cell type were used, but the FBS content was increased to 15% and further supplemented with 1% MEM Non-Essential Amino Acids (NEAA, Thermo Fisher Scientific) and 4 μg/mL Polybrene (Sigma-Aldrich, St. Louis, MO, USA). To inhibit NMD in HDFs, cells were incubated in growth media with 200 μg/mL cycloheximide (CHX) (Sigma-Aldrich) for 4 h or 24 h before collection. To generate cell lines stably expressing dCas9-ST-mCherry-BSD and P65-HSF1-GFP-NeoR, cells were selected via flow cytometry. Briefly, cells were prepared by triturating in Dulbecco’s phosphate-buffered saline (DPBS; Thermo Fisher Scientific) supplemented with 2% FBS and 1% PenStrep at a density of 1×10^7^ cells/mL and immediately sorted for mCherry- and GFP-positive cells using BD FACSFusion Cell Sorter (BD Biosciences) and collected in a fresh growth medium supplemented with 20% FBS and 1% PenStrep. To select gRNA-BFP-expressing cells for single-cell RNA-seq, cells were sorted by flow cytometry using the BD FACSymphony S6 Cell Sorter (BD Biosciences). The sorting buffer used was DPBS containing 5% FBS and 0.5mM EDTA, while collection buffer used was 1% BSA in DPBS. Untransduced cells and cells expressing GFP, BFP, and/or mCherry were used to adjust the voltage and set the gates.

iNeurons were derived as previously described with modifications.^53^ Transduction of HDFs with lentivirus delivering the p.TNA vector transgene was conducted at multiplicity of infection (MOI) 20 in HDF media containing Polybrene (4 μg/mL). Transduced fibroblasts were selected with 1 μg/mL of puromycin. Six-well plates or 35 mm dishes plates were coated in rhLaminin-521 solution (0.5 μg/cm2; Thermo Fisher Scientific) in DPBS and left overnight at 37°C. The following day, wells were washed thrice with DPBS. HDFs were seeded in coated plates at a density of 2.8 × 10^5^ cells/cm^2^. The media was aspirated 24 h later, cells were washed once with DPBS, and neuronal conversion (NC) media was added. NC media were made fresh before use and consisted of a 1:1 ratio of Neurobasal A and DMEM/F12 supplemented with (1% v/v) Pen-Strep, B27 and N2, and 1 μg/ml Laminin-521 (all from Thermo Fisher Scientific); 100 μg/ml db-cAMP and 2 μg/ml doxycycline (from Sigma Aldridge); and 100 ng/ml Noggin, 0.5 μM LDN-193189, 0.5 μM A83-1, 3 μM CHIR-99021, 5 μM Forskolin, and 10 μM SB-431542 (all from Stem Cell Technologies, Vancouver, BC, Canada). NC media were either replenished every second day in an initial experiment but optimized to give rise to an alternative replenishment regime: media were changed every day in the first week, every second day in the second week, and half media changes every second day in the third week of culture. Extended culturing of iNeurons past 21 days was performed using maturation media consisting of BrainPhys (Stem Cell Technologies) supplemented with 1% (v/v) Pen-Strep, B27 and N2, 1 μg/ml Laminin-521, 100 μg/ml db-cAMP (Sigma Aldridge), and 20 ng/ml of both GDNF and BDNF (R&D Systems, Minneapolis, MN, USA). Half media changes occurred every second day. Where indicated, iNeurons were treated with 100 μg/mL CHX for 24 h.

### RNA isolation, cDNA synthesis, and PCR

RNA extraction from HEK293T, HDF, and BJ-5ta was performed using RNeasy Plus Mini Kit (Qiagen) and RNase-free DNase Set (Qiagen) based on the supplier’s spin-column protocol. RNA extraction from iNeurons was performed using TRIzol (Thermo Fisher Scientific) as per manufactures protocol, with further processing using the RNeasy Plus Mini Kit and RNase-free DNase Set. RNA concentrations were determined using either Qubit RNA BR or HS Assay Kit (Thermo Fisher Scientific) as per the manufacturer’s instructions. cDNA from RNA was generated using SuperScript IV Reverse Transcriptase (Invitrogen) and Random Hexamers (Invitrogen) carried out based on manufacturer’s protocol. Briefly, 500 ng–2 μg of RNA was used for the cDNA synthesis reaction mixture, which was incubated at 23°C for 10 min to anneal the primers, then at 50°C for 60 min for the cDNA synthesis, and at 80°C for 10 min to inactivate the process. The resulting cDNA was diluted in deionized H_2_O at a 1:3 ratio prior to use in subsequent reactions. Real-time quantitative polymerase chain reaction (real-time qPCR) was performed using either the Power SYBR Green PCR Master Mix (Applied Biosystems) or the TaqMan Fast Advanced Mastermix (Applied Biosystems) with β-Actin (*ACTB*) as the housekeeping gene. The primers and Taqman probes (Thermo Fisher Scientific) used are listed in Table S1. The reactions were performed using standard cycling parameters on Step One Plus Real-Time PCR system (Applied Biosystems), and data were collected using StepOne Software v2.3 (Applied Biosystems). For the nuclease assay, PCR was performed on genomic DNA flanking the gRNA target sites using Taq DNA Polymerase (Roche) combined with FailSafe PCR 2X PreMix Buffer J (Lucigen) performed according to the manufacturer’s recommended cycling temperatures with annealing temperature set at 60°C. For patient cDNAs, PCR was performed with primers flanking the variant using Phusion High-Fidelity DNA Polymerase (Thermo Fisher Scientific) combined with the 5X Phusion GC Buffer, and 15% DMSO performed based on the supplier’s standard cycling temperatures with annealing temperature set at 60°C. Primers are listed in Table S1.

### Short-read RNA-seq

Library construction for short-read RNA-seq (srRNA-seq) was performed by the South Australian Genomic Centre (SAGC, Adelaide, Australia). In brief, the quality of RNA was first assessed based on the RNA integrity number evaluated using the 2100 Bioanalyzer system (Agilent, Santa Clara, USA) as per the manufacturer’s instructions. Libraries were generated using the Universal Plus RNA-Seq Library Kit (Tecan, Mannedorf, Switzerland) using Poly(A) selection (for transactivation samples) or rRNA depletion (iNeuron samples) as per manufacturer’s instructions. Conversion to MGI library was performed using the MGIEasy Universal Library Conversion Kit (MGI, Shenzhen, China). The MGI-compatible libraries were pooled in equimolar concentration and sequenced on the DNBSEQ-G400 Flow Cell Large (MGI, Shenzhen, China) to a minimum of 8 × 10^7^ paired-end 150 bp reads. Each sample was sequenced to a depth of ∼80 × 10^6^ 150 bp paired-end reads. FASTQ files were aligned and mapped to the human genome assembly GRCh38/hg38 with HISAT2, StringTie, and Ballgown.^54^ Salmon^55^ was used to generate read counts. Differential gene expression was performed using edgeR^56^ with biomaRt^57^ used to assign ENsembl IDs to gene symbols. Log fold change and adjusted *p* values were generated for comparisons between day 0 and other time point comparisons for iNeurons. To obtain junction read counts, a custom file was generated, including annotations for all transcripts of interested from Gencode, and junction reads were extracted using Rsubread Bioconductor package (for mapping, quantification and variant analysis of sequencing data) and seqinr (to retrieve and analyze biological sequences) using featureCounts, juncCounts.^58^

### Oxford nanopore amplicon sequencing

Library preparation of amplicons was carried out using the native barcoding amplicons protocol (version NBA_9093_v109_revC_12Nov2019) and sequenced on a MinION Mk1B. Super accuracy base calling was performed with MinKNOW (version 23.07.12, Guppy version 7.1.4). Sequences were mapped to human reference genome (GRCh38, GenBank: GCA_000001405.15) using minimap (version 2.17) with the default setting for spliced nanopore sequence data^59^ and visualized using the Integrative Genomic Viewer (IGV).

### scRNA-seq

Stable cell lines HEK293T-dCas9-ST-PH Clone 7 and BJ-5ta-dCas9-ST-PH Clone A were transduced with the pooled gRNA library targeting 40 genes (160 gRNAs in total) at a low MOI of 3 and 10, respectively, to achieve a transduction efficiency of ∼30%. Day 4 post-transduction, cells were sorted for BFP by flow cytometry. A total of 20,000 cells were targeted for each stable cell line resulting in ∼125 cells analyzed per gRNA. Single-cell RNA-seq (scRNA-seq) libraries were prepared using Chromium Next GEM Single-Cell 3′ Reagents Kits v3.1 (10× Genomics) following the manufacturer’s protocols. Briefly, single-cell suspensions of 10,000 cells per lane (2 lanes per cell line) were loaded on Chromium Chip G to generate single-cell Gel Beads in Emusion (GEMs). cDNA amplification was performed with 11 cycles. Sample indexing was performed with 9 cycles for both the 3′ gene expression library and the gRNA library construction using Dual Index Plate TT, Set A (PN-3000431) and Dual Index Plate NT, Set A (PN-3000483), respectively. Prior to the sequencing of 3′ gene expression libraries, the 40 target genes and 200 control genes (selected based on having third quartile [Q3] gene expression values and low variance in different HDFs) were enriched using a Twist custom panel (Table S2) following the Twist Target Enrichment Standard Hybridization v1 protocol (Twist Bioscience). Amplification of the indexed targets post-hybridization was carried out with 12 cycles using KAPA 2× HiFi PCR Mix (Roche) and purified using 1.2× solid-phase reversible immobilization beads. Library size distribution and abundance were assessed with D5000 ScreenTape (Agilent), and accurate molarity concentrations were measured by qPCR using Illumina a p5 (5′-AATGATACGGCGACCACCGA-3′) and p7 (5′-AAGCAGAAGACGGCATACGAGAT-3′) PCR primer cocktail and library standards (1, 2, 5, 10, and 20 nM) on a CFX384 Real-Time PCR (Bio-Rad). Libraries were sequenced on a NovaSeq 6000 (Illumina) using an SP Reagent 100-Cycle Kit (Illumina) in a paired-end format, resulting in a total of >60 M reads for each pulldown-enriched cDNA library and >24 M reads for each gRNA library. Sequencing data were processed with cellranger (v7.1.0). The count matrix for scRNA-seq and the count matrix for the gRNA presence was integrated and processed with Seurat (v4.3.0) in R (v4.2.3). Cells without detectable gRNA expression served as negative controls in the analysis (*n* = 200). Detailed analysis and code are available at https://github.com/ryanlister/RNA-variant-assessment-Nicolas-et-al-2024-.git.

### Assay for transposase-accessible chromatin with high-throughput sequencing (ATAC-seq)

ATAC-seq was performed on three control HDF lines together with BJ-5ta and HEK293T lines as per the Omni-ATAC-seq protocol with slight modifications.^60^ Briefly, cells were grown to 80% confluence. ∼500,000 cells were resuspended and permeabilized on ice for 3 min in 50 μL ice-cold ATAC resuspension buffer (ATAC-RSB; 10 mm Tris-HCL pH 7.4, 10mm NaCl, 3mm MgCl_2_) containing 0.1% NP40 (Sigma Aldrich), 0.1% Tween 20 (Sigma Aldrich), and 0.01% Digitonin (Promega, Maddison, WI, USA). Following permeabilization, samples were resuspended in 1 mL ice-cold ATAC-RSB containing 0.1% Tween 20 and pelleted at 4°C at 500 g for 5 min. Cells and nuclei were resuspended in 100 μL of ice-cold ATAC-RSB before being counted prior to transposition. 50,000 cells were subjected to tagmention in 1× Tagmentation Buffer (1 m Tris-HCl pH 7.6, 1 m MgCl_2_, 10% Dimethyl Formamide, 0.1% Tween 20 [Sigma], and 0.01% Digitonin [Promega]) using 2.5 μL Tn5 loaded transposase (in-house made Tn5, 25 μg/mL final) in 50 μL final volume for 30 min at 37°C. Reaction was stopped and purified using a Bioline PCR Clean-up kit (Meridian Bioscience, Cincinnati, OH, USA) and eluted in 25 μL of H_2_O. Indexing PCR was performed in 50 μL reaction using NEBNext High Fidelity PCR master mix (New England Biolabs, Ipswich, MA, USA) with the following conditions: 72°C for 5 min, 98°C for 30 s, 8 cycles of 98°C for 10 s, 63°C for 30 s, 72°C for 1 min, and hold at 12°C. Final clean-up of product was performed using 1.0× Ampure XP beads (Beckman Coulter, Brea, CA, USA) and visualized on the Agilent D5000 TapeStation. ATAC-seq data were adapter and quality trimmed with fastp^61^ using standard settings followed by mapping with bowtie2^62^ against the human reference genome hg38 in parallel with gnu-parallel.^63^ Reads mapped to the mitochondrial genome and to the ENCODE Exclusion List Regions (ENCFF001TDO) were removed.^64^ Duplicate reads were identified and removed by samtools markdup^65^ prior to peak calling with MACS2 (–nomodel –extsize 150 – shift-75–gsizehs–keep-dupall).^66^ ATAC-seq peaks were intersected with +/− 2 kb of promoter annotations with bedtools intersect.^67^ Counts in promoter peaks were aggregated with bedtools multicov followed by library size and peak width normalization.

### Immunofluorescence and microscopy

Cells were fixed with 4% paraformaldehyde diluted in DPBS for 20 min at room temperature. Cells were processed for immunofluorescent staining as previously described.^68^ Primary antibodies and their dilutions include MAP2 (AB15452, 1:1000), NEUN (MAB377, 1:200), PSA-NCAM (MAB5324, 1:1000), TAU1 (MAB3420, 1:1000), and TUBB3 (T2200, 1:300), all from Sigma Aldridge, and NESTIN (ab92391, 1:250) and SYN1 (ab254349, 1:500), both from Abcam (Cambridge, UK). Quantification of immunostained iNeurons was performed as previously described.^69^ Fluorescence was viewed using either the Zeiss AxioImager M2, or Zeiss Vert.A1 microscopes (Carl Zeiss, Jena, Germany). Images were captured using Axiocam Mrm cameras and Axiovision v4.9.1 software (Carl Zeiss).

### Data resources and analysis

The 4,878 Mendelian disease genes were extracted from the Nijmegen Disease Gene Panel 3.2.0 (Radboud University Medical Centre, Nijmegen, Netherlands). The 3,000 neurological disease genes were derived from combining genes listed in PannelApp Australia’s “Intellectual disability syndromic and non-syndromic” panel (Version 0.5619) and “Progressive Neurological Conditions” panel (Version 14.216). Other disease gene lists are referenced from PanelApp Australia (versions provided in relevant figures). The number of variants associated with genes was extracted from either ClinVar Miner^70^ (accessed December 12, 2023) or Human Genome Mutation Database^71^ (HGMD Professional; accessed December 1, 2024). The minimum required sequencing depth (MRSD) test^72^ was performed using the recommended default parameters: splice junction read coverage = 8, proportion of splice junctions covered = 75%, confidence level = 95%. Gene ontology analyses were performed using ShinyGO v0.77 with results ranked based on fold enrichment and false discovery rate corrected *p* values.^73^ Genome visualization was performed using IGV and UCSC Genome Browser. Tissue expression data were extracted from either the Genotype Tissue Expression^8^ (GTEx) database (version 8) or Human Protein Atlas (HPA).^74^ Cap analysis of gene expression data used to identify transcriptional start sites (TSSs) were obtained from the FANTOM 5 project.^75^ Comparison of splicing of expressed neurological genes (*n* = 2,484) between CATs and CRTs was performed using MAJIQ-CAT.^76^ All data were statistically analyzed and displayed using either Microsoft Excel, GraphPad Prism 10, or EdgeR. Error bars and statistical analyses are described within figure legends.

## Results

### Defining the silent Mendelian genes

**Figure 1:**
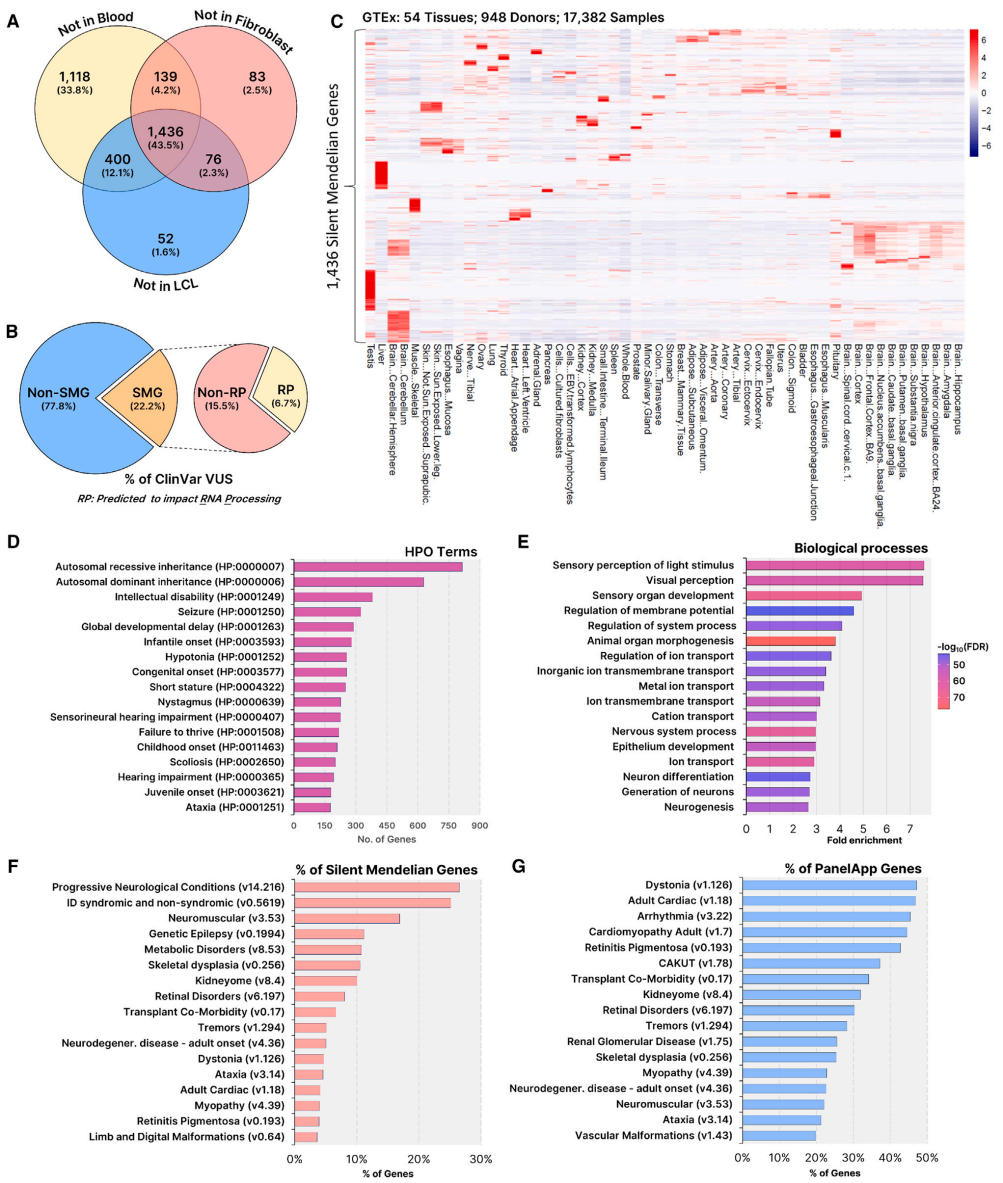
Silent Mendelian genes have restricted tissue expression and are prominently involved in neurological disorders (A) 1,436 Mendelian genes are silent. Analysis of 4,878 Mendelian disease genes (Nijmegen DG Panel 3.2.0) using minimum required sequencing depth (MRSD) identified 1,436 genes that are not sufficiently expressed in whole blood, LCLs, or HDFs for the purpose of conducting robust analysis of mRNA splicing using srRNA-seq. These genes are termed silent Mendelian genes (SMGs). (B) Large numbers of VUS are found in SMGs. From the catalog of VUSs in ClinVar, 22.2% of all are found in SMGs, of which ∼30% are predicted to impact RNA processing (RP; 6.66% of all ClinVar VUSs). (C) SMGs display highly restricted tissue-specific expression. Heatmap showing the level of mRNA expression (TPM) of each of the 1,436 SMGs across 54 different tissues taken from 948 donors (data obtained from GTEx Version 8). (D–G) Phenotypes, disease categories, and biological processes associated with SMGs. (D) Most frequently associated human phenotype ontology (HPO) terms. (E) Top-ranked gene ontology (GO) biological processes (analyzed via ShinyGO 0.77 using whole-genome background, ranked by Fold enrichment and false discovery rate [FDR]). (F–G) Disease types ranked based on (F) their contribution to the number of SMGs or (G) on the proportion of known associated genes that are silent. Disease gene lists referenced from PanelApp Australia (accessed December 12, 2023).

To assess the scale of the known disease genes with insufficient expression in CATs, we analyzed the 4,878 Mendelian disease genes (Nijmegen DG Panel 3.2.0) using the minimum required sequencing depth (MSRD) algorithm.^72^ This model calculates the srRNA-seq depth required for sufficient read coverage across splice junctions to robustly assess alternative splicing events using RNA obtained from different CATs (e.g., whole blood, lymphoblastoid cell lines [LCLs] and HDFs). We adopted the recommended MSRD parameter settings for our study (see subjects, material, and methods), albeit more stringent parameters would elevate MRSDs and further accentuate outomes.^72^ This analysis revealed that 1,436 (∼30%) of these Mendelian disease genes are not sufficiently expressed in any of these CATs to conduct robust analysis of splicing using srRNA-seq at any sequencing depth^72^ (Figure 1A; Table S3). We term these 1,436 genes the “silent Mendelian genes” (SMGs), noting a muscle biopsy sample could be used to obtain sufficient mRNA to assess a further 166 of them, although these are not routinely collected^72^ (Figure S1; Table S3). Of the 1,436 SMGs, 1,364 of them (95%) have an assigned VUSs in ClinVar, which total 283,353 SMG VUSs and equate to 22.2% of all VUSs in ClinVar^70^ (Figure 1B). The proportion of these SMG VUSs that affect RNA processing is unknown, but previous studies predicted this to be ∼30%.^14^ This is supported by queries to the HGMD wherein 38.3% of known pathogenic variants are predicted to impact RNA processing (including splice altering, nonsense, and frameshifting small insertions and deletion variants). Applying the conservative estimate that 30% of variants impact RNA processing suggests that ∼85,000 VUSs (or ∼6.66% of all ClinVar VUSs) may be found in SMGs for which functional RNA studies would be beneficial but challenging to perform due to lack of expression in CATs (Figure 1B). Most of the SMGs display highly restricted, tissue-specific expression based on analysis of GTEx data (Figure 1C). The most frequent human phenotype ontology terms associated with SMGs are intellectual disability (HP: 0001249), seizures (HP: 0001250), global developmental delay (HP: 0001263), and infantile onset (HP: 0003593) (Figure 1D; Table S4). Gene ontology reveals SMGs are enriched for ion and membrane transport genes involved in muscular and nervous system functions (Figures 1E and S1; Tables S5–S7). The largest proportion of SMGs are involved in disorders of the nervous system (Figure 1F), while greater than 40% of known dystonia, cardiac, and retinal disorder genes are silent (Figure 1G). These data define the silent Mendeliome and highlight its relevance to a large proportion of current VUSs associated with a range of disorders that manifest in specific organ systems, particularly the nervous system.

### Gene transactivation induces the expression of SMGs

**Figure 2:**
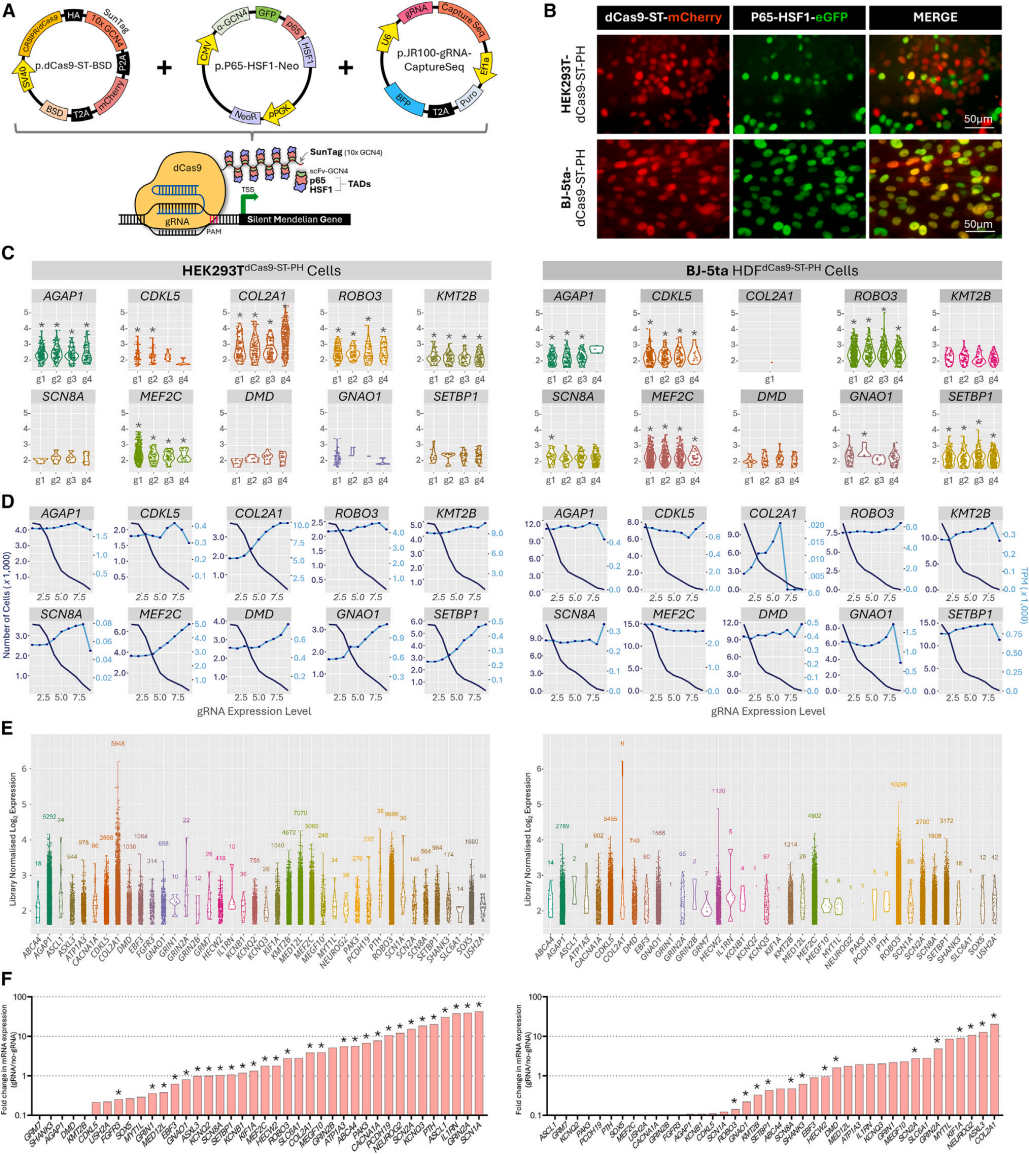
SMGs are conducive to gene transactivation (A) The dCas9-ST-PH-gRNA gene transactivation system. Co-expression of three transgenes results in the assembled transactivation complex on a gene promoter consisting of (1) the enzymatically dCas9 fused to a SunTag array (10 copies of GCN4 epitopes; dCas9-ST), (2) the hybrid p65 and HSF1 (heat shock factor 1) transcriptional transactivation domains (TADs) fused to a single-chain variable fragment (scFv) that recognizes the GCN4 epitope, and (3) the short gRNA, which directs the dCas9-ST-PH complex to the target gene promoter. The dCas9-ST can recruit up to ten copies of the hybrid transactivator P65-HSF1. (B) HEK293T and HDF clonal cell lines stably expressing dCas9-ST-PH. Representative images of HEK293T^dCas9−ST-PH^ and HDF^dCas9−ST-PH^ cell lines showing stable co-expression of transgenes encoding dCas9-ST (as reported by mCherry encoded in cis) and P65-HSF1 (as reported by EGFP encoded in cis). (C–E) Transactivation screen using single-cell transcriptomics. A pooled gRNA expression plasmid library (160 gRNAs; 4 gRNAs per gene, targeting 40 SMGs) was delivered by lentivirus to the stable HEK293T^dCas9−ST-PH^ and HDF^dCas9−ST-PH^ using a low multiplicity of infection to deliver ∼1 gRNA vector per cell. >20,000 cells per cell line were subjected to single-cell Perturb-seq using the 10× Genomics platform. (C) Cells expressing >6 molecules of a given gRNAs species were analyzed for expression of their target gene. ^∗^*p* < 0.05 (adjusted for multiple comparisons). (D) In general, the number of gRNAs per cell is positively associated with target gene expression and negatively associated with cell number analyzed. Data are pooled from all four gRNAs per gene. Dark blue lines are the number of cells, light blue lines are transcripts per million (TPM), and x axis is gRNA expression. (E) Expression levels of the 40 targeted SMGs in single cells. Each dot represents the expression of the target gene in a single cell analyzed. (F) Transactivation screen using bulk-cell transcriptomics. The pooled gRNA expression plasmid library was transfected to the stable HEK293T^dCas9−ST-PH^ or transduced into HDF^dCas9−ST-PH^ cells at high efficiency. Isolated RNA was subjected to srRNA-seq. Bar graph showing the transactivation of 40 SMGs (*n* = 4 biological replicates; ^∗^*p* < 0.05, Genewise statistical test).

To overcome the insufficient expression of SMGs for functional gene variant investigation, we initially employed gene transactivation technologies. We repurposed and further developed a third generation CRISPR activation (CRISPRa) system, known as deactivated Cas9 (dCas9)-Suntag.^77^^,^^78^ Our challenge was to engineer cell lines derived from individuals with gene variants in SMGs to co-express three transgenes encoding (1) an enzymatically dCas9 protein fused to a Suntag motif (10 copies of the GCN4 epitope; dCas9-ST), (2) the p65-HSF hybrid transcriptional activator fused to a single-chain variable fragment antibody that binds the Suntag GCN4 epitopes, and (3) gRNAs designed to direct the dCas9-ST and p65-HSF complex to the promoter of the desired silent genes. Ultimately, co-expression of these components (collectively called dCas9-ST-PH-gRNA) recruits multiple copies of the p65-HSF transcriptional activator to the promoter of a targeted SMG to induce gene expression (Figure 2A). The highly programmable nature of dCas9 enables targeting of theoretically any promoter of choice by simple alteration of the gRNA sequences.

We selected 40 SMGs (Table S8) to screen the efficacy of gene transactivation using the dCas9-ST-PH-gRNA design. These 40 SMGs were chosen based on a need for VUS assessment in our laboratory or otherwise known high *de-novo* variant burden in developmental disorders^79^ and collectively have >20,000 VUSs associated with them in ClinVar^70^ (Figure S2). To empower effective gRNAs design, we performed ATAC-seq on three different control HDF cell lines (and an HEK293T cell line) to identify open chromatin regions most suitable for gRNA placement and supplemented this information with other publicly accessible datasets (FANTOM5,^75^ ENCODE,^64^ GTEx,^8^ dbSNP^80^), which collectively informed us on gene isoform selection, TSSs, histone marks of active promoter regions, and regions of common genome variation (Figure S3). We combined these resources with the gRNA design tool E-CRISP^81^ to design and choose four gRNAs for each of the 40 SMGs (160 gRNAs in total) (Figure S3; Table S9). We validated the ability of a subset of 12 gRNAs targeting *AGAP1* (MIM: 608651), *PAK3* (MIM: 300142), and *GRM7* (MIM: 604101) to recruit Cas9 to their promoters using a Cas9 nuclease assay^49^ (Figure S4). We then engineered both HEK293T and HDF (BJ5a) stable clonal cell lines that express dCas9-ST-PH (all components of the transactivation system except the gRNA, HEK293T^dCas9−ST-PH^, and HDF^dCas9−ST-PH^ cells, respectively) (Figures 2B and S5). In this design, the HEK293Ts represented an easy-to-manipulate surrogate fibroblast cell type, while HDFs represent a CAT-derived cell type and our ultimate target. Delivery of previously published gRNAs targeted to *IL1RN*^82^ (MIM: 147679) activated its expression as expected (Figure S5). Next, the 160 gRNAs targeted to the 40 selected SMGs were cloned as a pooled library into a gRNA expression vector compatible with the Perturb-seq gRNA screening approach^83^^,^^84^ (Figure S6; Table S10). In this approach, the pooled library of gRNAs was delivered at low dosage to the HEK293T^dCas9−ST-PH^ and HDF^dCas9−ST-PH^ cell lines such that each cell in the respective cultures received on average no more than a single gRNA type. The cell population was then subjected to single-cell RNA-seq (scRNA-seq) wherein the gRNA expressed in each cell was identified, and the expression of the gene targeted by the gRNA was measured in the same cell.^83^^,^^84^ Cells without any detectable gRNA expression serve as negative controls (*n* = 200). The gRNA pool was delivered by lentivirus at a low MOI to both HEK293T^dCas9−ST-PH^ and HDF^dCas9−ST-PH^ cells and purified by puromycin selection followed by fluorescent activated cell sorting (FACS). Isolated cells were subjected to scRNA-seq via the Peturb-seq 10× Genomics pipeline.^83^^,^^84^ We found significant upregulation of 9/40 genes in HEK293T^dCas9−ST-PH^ cells and 8/40 genes in HDF^dCas9−ST-PH^ cells for which at least one of the four gRNAs transactivated its target gene (Figures 2C and S7). This included four genes that were significantly transactivated in both cell lines (*AGAP1*, *CDKL5* [MIM: 300203], *MEF2C* [MIM: 600662], and *ROBO3* [MIM: 608630]), and other genes that were cell-type specific (e.g., *COL2A1* [MIM: 120140] in HEK293T^dCas9−ST-PH^ cells and *SETBP1* [MIM: 611060] in HDF^dCas9−ST-PH^ cells) (Figures 2C and S7). Further interrogation revealed that for many targeted genes, the abundance of the gRNAs expressed in the cell was directly related to the level of target gene expression and indirectly related to the number of cells contributing to the analysis (Figures 2D and S8). These relationships in genes not reaching significance suggest (1) they are likely amenable to transactivation in response to higher gRNA expression per cell, and (2) the lack of significant transactivation was reflective of an insufficient number of cells analyzed (on average 125 cells per gRNA per cell type; >20,000 cells per cell type in total). We therefore aggregated the gRNA-wise analysis into a gene-wise analysis by identifying individual cells expressing one of the 40 target genes, and quantifying the level of expression, independent of gRNA thresholds. These data reveal that for all genes analyzed, at least some cells had elevated SMG expression, although such cells were still rare (<100 cells) for 16/40 and 25/40 of genes analyzed in HEK293T^dCas9−ST-PH^ and HDF^dCas9−ST-PH^ cells, respectively. Subsequent collapsing of the single-cell data into a pseudo-bulk cell RNA-seq analysis provided further support that most genes show at least some degree of transactivation, more potently observed in HEK293T^dCas9−ST-PH^ compared to HDF^dCas9−ST-PH^ cells (Figures 2E and S9). Given these data, we reasoned that delivery of higher dosage of gRNAs to cells would more potently induce gene expression in the cell populations and facilitate assessment of gene transactivation using bulk srRNA-seq. The vector library was therefore delivered to cells using high efficiency transfection (for HEK293T^dCas9−ST-PH^ cells) or high efficiency transduction (for HDF^dCas9−ST-PH^ cells). Subsequent srRNA-seq revealed significantly increased expression of 29/40 genes in HEK293T cells, and 14/40 genes in HDFs (Figure 2F). Collectively these data reveal that most of the 40 SMGs tested were amenable to some level of transactivation in HEK293T cells, with a less potent effect in HDFs. The efficacy and magnitude of transactivation was variable between gRNAs, genes, and cell types and, in general, was favored by high gRNA expression.

### A highly multiplexed robust transactivation system for HDFs

**Figure 3:**
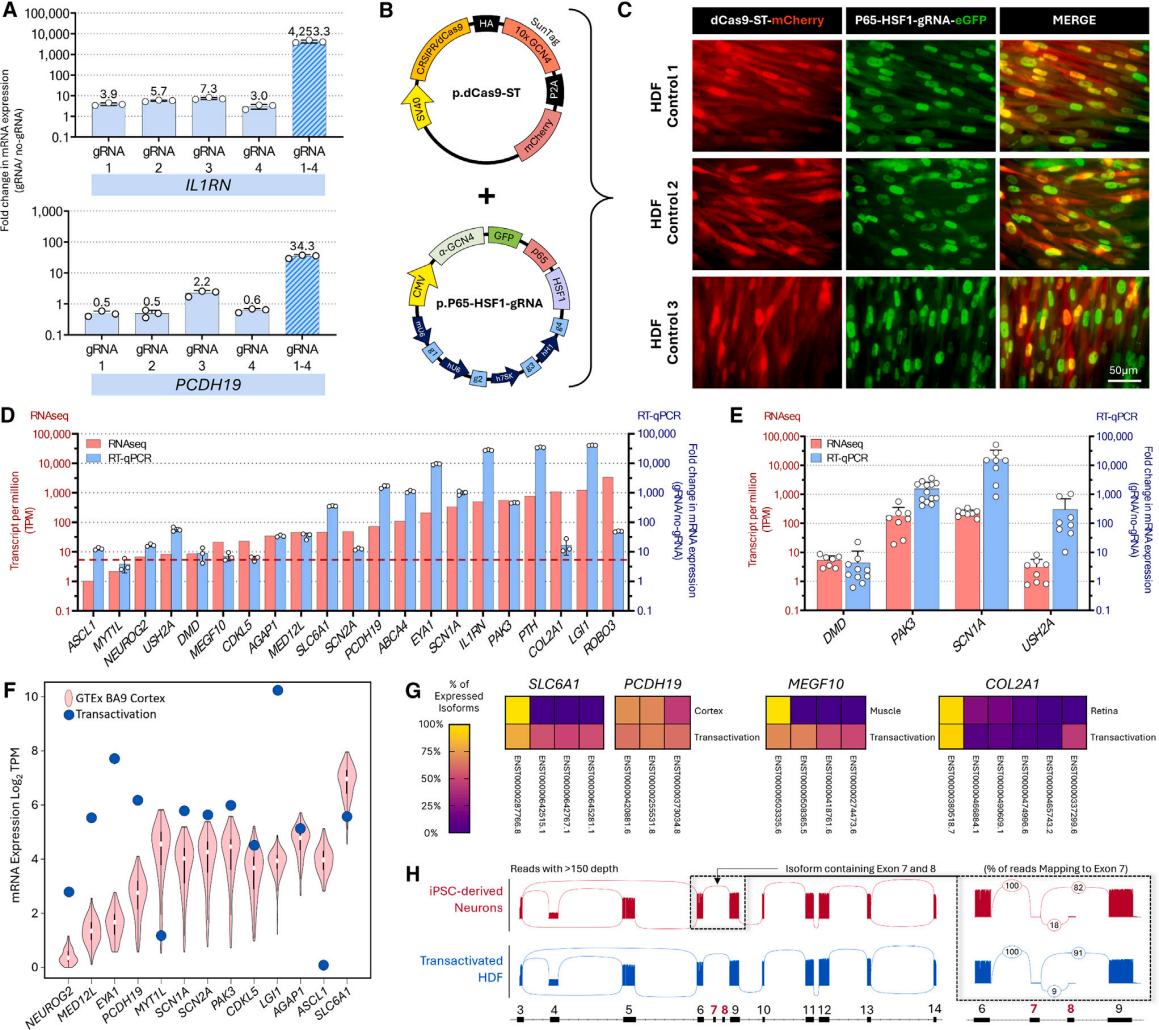
Robust Transactivation of SMGs in HDFs (A) Comparison of transactivation levels of *IL1RN* and *PCDH19* using single gRNAs versus a multiplex of four gRNAs. Relative gene expression analyzed via real-time qPCR with values normalized to *ACTB* and expressed relative to the negative control (dCas9-ST-PH-no gRNA). Error bars respresent standard deviation. (B) The dCas9-ST-PH-gRNA complex was engineered across two lentiviral transgenes with fluorescent reporters. (C) Optimized transient delivery of dCas9-ST-PH-gRNA complex to HDFs. Highly efficient lentiviral co-delivery of dCas9-ST and P65-HSF1-gRNA transgenes in three control HDFs. Representative images showing co-expression of p.dCas9-ST transgene (mCherry) and p.p65-HSF-gRNA (eGFP) 72 h after transduction. (D) Co-expression of dCas9-ST-PH complex and 4 gRNAs successfully transactivates expression of many SMGs in HDFs. Bar graph showing the individual transactivation levels of 20 SMGs and *IL1RN* mediated by co-expression of dCas9-ST-PH complex and four gRNAs. Expression levels (TPM) generated from srRNA-seq (red) and relative gene expression generated from real-time qPCR (blue) with values normalized to *ACTB* and expressed relative to negative control (dCas9-ST-PH with no gRNA). Error bars respresent standard deviation. Red dotted line corresponds to TPM = 5. (E) SMGs can be robustly activated across multiple experiments and HDFs. Bar graph showing the transactivation of *DMD*, *PAK3*, *SCN1A*, and *USH2A* mediated by dCas9-ST-PH-gRNA in multiple different HDF lines. The bar graph data presents the mean and standard deviation from the biological replicates, with each dot plot representing a different cell line. Data presented are expression levels (TPM) generated from srRNA-seq (red bars) and relative gene expression generated from real-time qPCR with values normalized to *ACTB* and expressed relative to the negative control (dCas9-ST-PH with no gRNA; blue bars). (F) Transactivated SMGs expression levels are comparable to endogenous expression levels in CRTs. Violin plots show endogenous expression of a subset of SNGs in the adult cerebral cortex. The blue dots show the expression of the same genes transactivated in HDFs. Data presented are expression levels (TPM) calculated independently for cortex data accessed from GTEx (Version 8) and transactivated HDF data generated from srRNA-seq, respectively. (G) Transactivated genes in HDFs express diverse isoforms. Comparison isoforms expressed in CRTs (extracted from GTEx Version 8) with transactivated HDFs (srRNA-seq). (H) Complex and rare splicing events are observed using transactivation. Sashimi plot displaying complex splicing patterns of *PAK3* in iPSC-derived neurons recapitulated following transactivation in HDFs. Only events with read depth greater than 150 are shown. Insert highlights rare isoform containing exon 6 and 7. All reads that map to exon 6 are shown.

Of the cell types derived from CATs, HDFs are the best at recapitulating the splicing patterns of genes observed in CRTs.^76^^,^^85^ This was supported by our own interrogation of non-silent neurological disorder genes (Figure S10). We therefore focused on further modifying the transactivation system and its delivery to HDFs for the end purpose of analyzing the impact of gene variants in SMGs using CATs from many different individuals. Given that the single-cell and bulk gRNA screens suggested modest and variable potency of single gRNAs to induce SMG expression, we tested if multiplexing the expression of all four gRNA per gene simultaneously increased efficacy.^82^ Indeed, multiplexing gRNA expression targeted to *IL1RN* and *PCDH19* (MIM: 300460) resulted in more potent gene expression than single guides alone (582× and 15×, respectively) (Figure 3A). We thus created 4-plex gRNA expression cassettes of 20 SMGs together with *IL1RN* (which served as a positive control,^82^ albeit also an SMG^86^) (Table S10). We proceeded to optimize the delivery of the vector transgenes to cells in a transient manner, making it efficient to conduct experiments across many different cell lines and to gain maximal expression of gRNAs and other components. First, to reduce the number of vectors required for transactivation (and hence increase delivery to cells), we cloned the multiplex gRNA cassettes into the plasmids expressing p65-HSF1 (Figures 3B and S11). Co-expression with the second vector encoding dCas9-ST in cells reconstitutes the highly multiplexed system (collectively called dCas9-ST-PH-gRNA) in which up to 40 p65-HSF transcriptional activators are recruited to each promoter (Figure S11). The transactivation of each gene was tested one at a time, initially in HEK293T cells by co-transfection. The mRNA expression of all genes was tested by real-time qPCR and found to be elevated, ranging from 3 to 17,000 times higher than controls (expression of dCAS9-ST-PH without gRNAs) (Figure S11). Transactivation levels of *IL1RN* were sufficient to detect protein by western blot (Figure S11). We redesigned and tested alternative gRNAs for two genes that displayed modest transactivation levels, which improved transactivation for *DMD* (18 times higher; MIM: 300377), but not *MYT1L* (1.3 times higher; MIM: 613084) (Figure S11). All vectors were then packaged into lentiviral particles to facilitate co-delivery to HDFs. We optimized a protocol for transient lentiviral co-transduction of vectors to express dCAS9-SPH-*PAK3*-gRNA in HDFs using readouts of both live-cell transgene expression and endpoint transactivation of *PAK3* mRNA expression (Figures 3C and S12). Next, the optimized three-day transient transduction protocol was applied to test the ability of dCAS9-ST-PH-gRNA system to transactivate the expression of the selected 20 SMGs in HDFs one gene at a time (Table S10). The mRNA expression levels of the 20 SMGs were analyzed using both real-time qPCR and srRNA-seq. All genes tested were found to be transactivated, with increased mRNA expression levels ranging from 6 to 90,000 times greater than negative controls (no gRNA) by real-time qPCR (Figure 3D). This increase in relative expression aligned well with mRNA quantification using srRNA-seq, which ranged from 1 to >3,300 transcripts per million (TPM) (Figure 3D). We aimed to gauge how many of the 20 transactivated genes achieved expression levels conducive to downstream srRNA-seq-based assessment of splicing. We found that the median number of exon junction spanning reads (i.e., reads critical to map splicing events) for each transactivated SMGs ranged between 4 and >14,000 per gene (Figure S13; Table S11). In alignment with the MRSD parameter used to define SMGs (see subjects, material, and methods), 17/20 (85%) transactivated genes achieved >7 read counts across >75% of junctions thus illustrating compliance with srRNA-seq-based analysis of splicing under this definition (Figure S13; Table S11). We also found that the median junction read depth of each transactivated SMG was highly correlated with its TPM (Pearson’s correlation r = 0.97, *p* = 3.672e-13) (Figure S13). Thus, while utilizing TPM as a proxy for compliance with srRNA-seq-based splicing analysis has caveats,^72^ these data provide support and enable alignment with other RNA diagnostic benchmarking studies utilizing TPM thresholds. For example, genes with TPM >5 are suggested to be compliant for srRNA-seq-based assessment of splicing,^11^ which was achieved for 18/20 (90%) of transactivated SMGs tested (Figure 3D). The remaining 2/20 had TPM >0.5, suggesting downstream studies would require either real-time PCR-based methods or deeper sequencing. We next repeated transactivation experiments several times for a set of SMGs (*DMD*, *PAK3*, *SCN1A* [MIM: 182389], and *USH2A* [MIM: 608400]) to reveal robust inter-experimental transactivation across multiple different HDF cell lines (Figure 3E). For the subset of neurological disorder genes tested, the expression levels (TPM) in transactivated HDFs were generally aligned with the median expression observed in the CRT of adult cortex (with exceptions) (Figure 3F). In one example, the expression of *PCDH19* (MIM: 300460), a gene involved in developmental epileptic encephalopathy (MIM: 300088), was 10 times higher in transactivated HDF than that observed in the brain cortex as reported in GTEx^8^ (Figure 3F). An advantage of gene transactivation is that it potentially permits the investigation of variant impact across multiple gene isoforms (all isoforms driven from a given promoter). Indeed, we detected a diversity of gene isoforms following gene transactivation in HDFs, including those prominently expressed in the CRT (Figures 3G and S14). For example, the diversity of *SLC6A1* (MIM: 137165), *PCDH19*, *MEGF10* (MIM: 612435), and *COL2A1* isoforms detected in transactivated HDFs closely resemble that of their CRT. We investigated *PAK3* isoform diversity in further detail given that it has over 25 different annotated isoforms. We compared *PAK3* splicing in transactivated HDFs and human iPSC-derived neurons using Oxford Nanopore long-read sequencing of amplicons generated by PCR of *PAK3* cDNA. Gene transactivation captured the major *PAK3* isoforms expressed in neurons, as well as extremely rare isoforms encoding exons 6 and 7 (Figure 3H). Collectively, these data reveal that combining multiplexed gRNA expression together with an already highly multiplexed dCas9-ST-PH system for transient gene transactivation is a robust way to obtain mRNA of SMGs from HDFs that can recapitulate the abundance, isoform diversity and local splicing events of the CRT. Of the 20 SMG that were transactivated, the majority (90%) achieved levels considered conducive to srRNA-seq-based assessment of splicing based on TPM.

### Investigating variants in SMGs using transactivation

**Figure 4:**
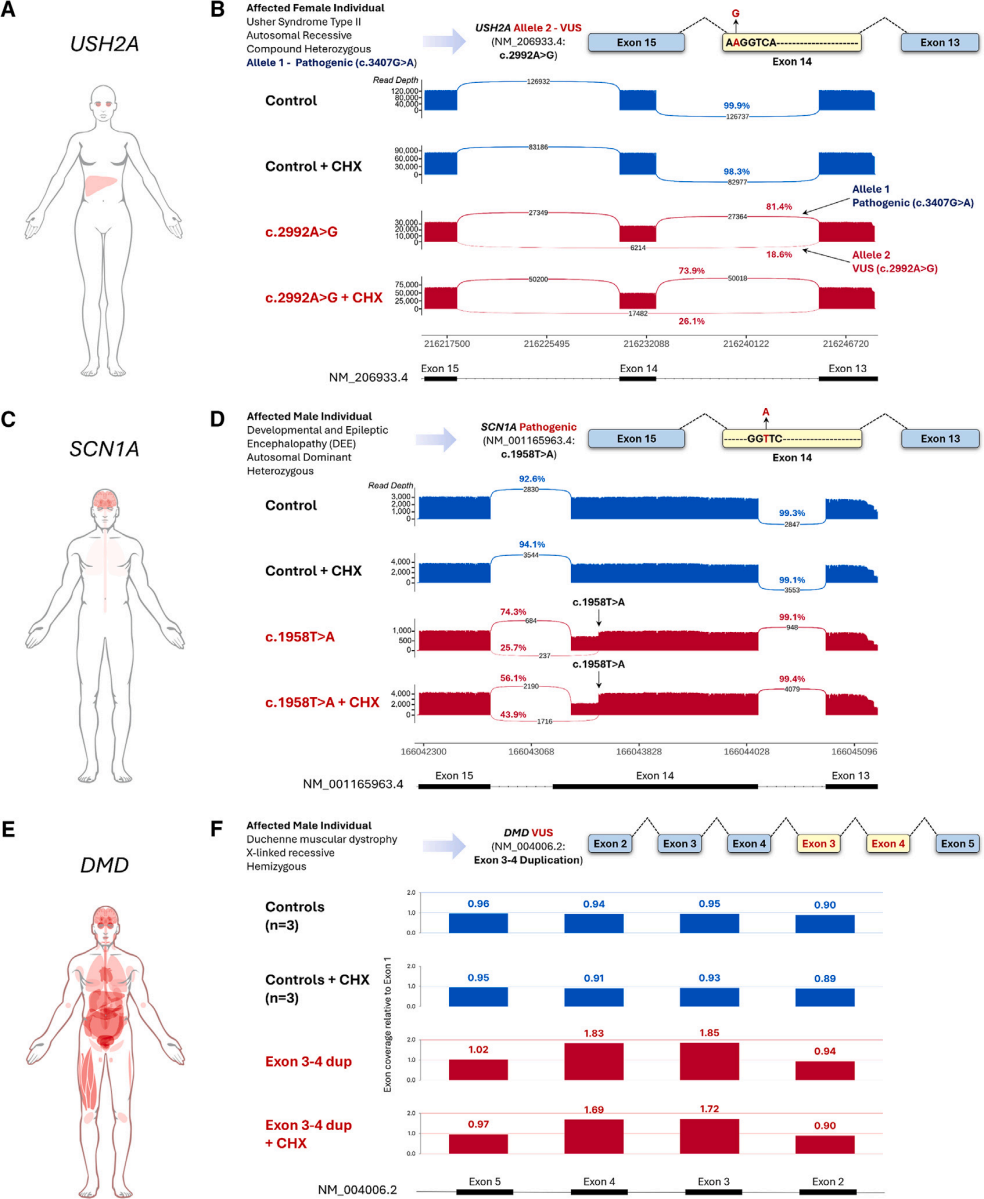
Investigation of RNA variants in SMGs using transactivation of HDFs (A) Illustration of *USH2A* mRNA expression (red) in human adult tissues as reported by the Human Protein Atlas (HPA). (B) Diagram depicts the *USH2A* variant under investigation. Sashimi plots report *USH2A* mRNA splicing. Data derived from Oxford Nanopore long read sequencing of RT-PCR amplicons (exons 13–16) produced using RNA isolated following transactivation of *USH2A* in HDFs derived from healthy control and affected individuals in the presence and absence of cycloheximide (CHX). Arrows in the sashimi plots specify the reads coming from the alleles with pathogenic variant and allele with VUS as segregated by allelic phasing. (C) Illustration of *SCN1A* mRNA expression (red) in human adult tissues (HPA). (D) Diagram depicts the *SCN1A* variant under investigation. Sashimi plots report *SCN1A* mRNA splicing. Data derived from Oxford Nanopore long-read sequencing RT-PCR amplicons (exons 13–17) produced from RNA isolated following transactivation of *SCN1A* in HDFs derived from healthy control and affected individuals in the presence and absence of CHX. Arrows on the sashimi plot indicates the position of the pathogenic variant. (E) Illustration of *DMD* mRNA expression (red) in human adult tissues (HPA). (F) Diagram depicts the *DMD* variant under investigation. Graphs represent relative read depth of reported across DMD exons 2–5 as determined using long read sequencing of RT-PCR amplicons produced from RNA isolated following transactivation of *DMD* in HDFs derived from healthy control and affected individuals in the presence and absence of CHX. Note read depth is 1.8 times greater (∼double) in exons 3– and 4 only in samples from the affected individual and is not influenced by CHX.

To demonstrate the utility of transactivation of SMGs we investigated the impact of variants suspected to be the cause of Mendelian disease and predicted to impact RNA processing. We obtained HDFs derived from the affected individuals and applied our transient transactivation protocol (Figure S12D). First, we investigated a VUS in *USH2A* in which recessive loss-of-function variants cause Usher syndrome (MIM: 276901), featuring moderate to profound hearing loss from birth and childhood onset retinitis pigmentosa leading to loss of vision. *USH2A* is only expressed in the eye, liver, and testis (Figures 4A and S15). Specifically, we investigated a VUS ( c.2992A>G [GenBank: NM_206933.4] [p.Arg998Gly) found in *trans* with a known pathogenic variant (c.3407G>A [p.Ser1136Asn]) (Figure 4B). The missense VUS was predicted to be benign by several algorithms; however, the single base pair change altered the penultimate base of exon 14 conceivably impacting splicing despite weak *in silico* predictions (SpliceAI^26^) (Figure S15). We transactivated *USH2A* in three control HDFs and the HDF derived from the affected individual and treated the cells with or without CHX for 4 h, a translational blocker that therefore inhibits NMD. RNA was then subjected to srRNA-seq. While in controls, the reads supported canonical splicing of exons 13 to 14, and 14 to 15 in the variant sample reads were found to skip exon 14 (Figure S15). A targeted PCR coupled with long-read Oxford Nanopore sequencing and allelic phasing (based on the c.2992A>G and c.3407G>A variants in *trans*) confirmed that 98% of all reads from the VUS allele (and 18.6% of all reads) skipped exon 14 (Figures 4B and S15). Skipping exon 14 deletes 184 bp of the transcript (c.2810_2993del) and creates a protein coding frameshift that results in a premature termination codon in exon 15, p.Gly937Aspfs^∗^13. This truncates the major isoform open reading frame by 82% and is predicted non-functional as it lacks the majority of key protein domains. The c.2810_2993del PTC-containing transcript was also found to be slightly enriched after the 4 h CHX treatment aligned with potential regulation by NMD, albeit warranting further examination with longer CHX treatments (e.g., 24 h as used below). Collectively, these data reveal deleterious impacts of the VUS on the *USH2A* allele.

Next, we investigated the mechanisms of action of a known pathogenic variant in *SCN1A*, haploinsufficiency of which causes a developmental epileptic encephalopathy called Dravet syndrome (MIM: 607208). *SCN1A* is only expressed in the brain, lung, and fallopian tube (Figures 4C and S16). The affected individual carried a nonsense variant in exon 13 (c.1958T>A [GenBank: NM_001165963.4] [p.Leu653^∗^]) with the assumed mechanism of haploinsufficiency (Figure 4D). However, the individual had a very severe presentation of Dravet syndrome ultimately resulting in sudden unexpected death during epilepsy. Intriguingly, Splice AI^26^ predicted that the variant strengthens a splice donor one base downstream (donor gain delta score = 0.72) (Figure S16). We transactivated *SCN1A* in three control HDFs and the HDF derived from the affected individual, treated the cells with or without CHX for 24 h, and performed srRNA-seq to investigate. In controls, canonical splicing of exon 13 to 14 of the main isoform was found in all cell lines (Figure S16). The variant indeed caused use of an internal exon 13 splice donor (54 bp upstream of the canonical splice donor of the main transcript), and although still encoding the nonsense codon, the mRNA species was apparently expressed at considerable levels, suggesting at least partial escape from NMD (Figure S16). The aberrant splicing event was confirmed using a targeted PCR coupled with long-read Oxford Nanopore sequencing (Figures 4D and S16). In this PCR-based assay, 25.7% of reads were found mis-spliced in the absence of CHX aligned with a partial escape from NMD as noted above. However, the addition of CHX did increase the proportion of mis-spliced reads to 43.9%, suggesting escape is indeed partial rather than complete (Figures 4D and S15). We further observed the partial escape phenomena using long-read sequencing of two additional, independent PCR amplicons (Figure S16). Whether mis-spliced reads escaping NMD culminate in the translation of the predicted 653 amino acid N-terminal SCN1A peptide remains to be determined but may help explain the severe nature of the individual’s phenotype.

We also investigated a variant in *DMD*, an X chromosome gene for which loss of function gives rise to the male neuromuscular disorders Duchene or Becker muscular dystrophy (MIM: 310200 and 300376). *DMD* is expressed in several tissues, but the expression levels of disease-relevant isoforms are insufficient for analysis in CATs (a muscle biopsy is typically needed) (Figures 4E and S17). The individual was a four-year-old male presenting with mild hypertrophy of the gastrocnemius and biceps, early motor delay, and limb girdle weakness with elevated serum creatine kinase levels (25,000 U/L, reference levels <180 U/L). Diagnostic massively parallel sequencing and multiplex ligation-dependent probe amplification identified a duplication of exons 3 and 4 that is predicted to be in-frame (c.(93 + 1_94-1)_(264 + 1_265-1)dup [GenBank: NM_004006.2]), but how the mRNA is actually spliced remained undetermined, and as such, the variant was classified as a VUS (Figure 4F). We transactivated *DMD* in three control HDFs and the HDF derived from the affected individual, treated the cells with or without CHX, and performed srRNA-seq. In controls, canonical splicing through exons 2–5 was found in all control cell lines; however, reads from the variant cell line were suspiciously absent in this region (Figure S17). We investigated further using targeted real-time PCR spanning the duplicated exons and found an increased size of the PCR product from the affected individual consistent with duplication of exons 3 and 4 in the mRNA (Figure S17). Long-read Oxford Nanopore sequencing of the PCR products was performed and sequences mapped to the reference transcript GenBank: NM_004006.2. Reads were successfully mapped in control samples as expected, while in the sample from the affected individual, reads mapping to exon 4 contained downstream sequences that did not align to intron 4 or exon 5 (Figure S17). We queried the misaligned sequences using the BLAST-Like Alignment Tool, which aligned them to *DMD* exons 3 and 4 thus revealing duplications of exons 3 and 4 in the mRNA. (Figure S17). We then remapped the reads using LAST, an approach that also utilizes a BLAST-like algorithm to enable reassignment of the misaligned segments of sequence to exons 3 and 4 and determined exon-level read counts to quantify the number of exons in the mRNA.^87^ As expected, reads mapping to exons 3 and 4 were in the same proportion to exons 1, 2, and 5 in controls while they were almost double (1.8 times greater) in samples from the affected individual. (Figure 4F). Collectively, these data confirm the presence of novel mRNAs encoding the tandem duplication of exons 3 and 4, which we validated using RNA isolated from a muscle biopsy of the affected individual (Figure S18). The duplication event is in-frame and inserts 57 amino acids that disrupt the actin-binding domain of dystrophin and is predicted to be highly deleterious to its function. In aggregate, these variant investigations in *USH2A*, *SCN1A*, and *DMD* enabled by transactivation of HDFs derived from affected individuals support the use of transactivation for the purpose of functionally investigating variants suspected of altering RNA processing in SMGs.

### Transdifferentiation of HDFs directly to neurons induces expression of silent neurological genes

**Figure 5:**
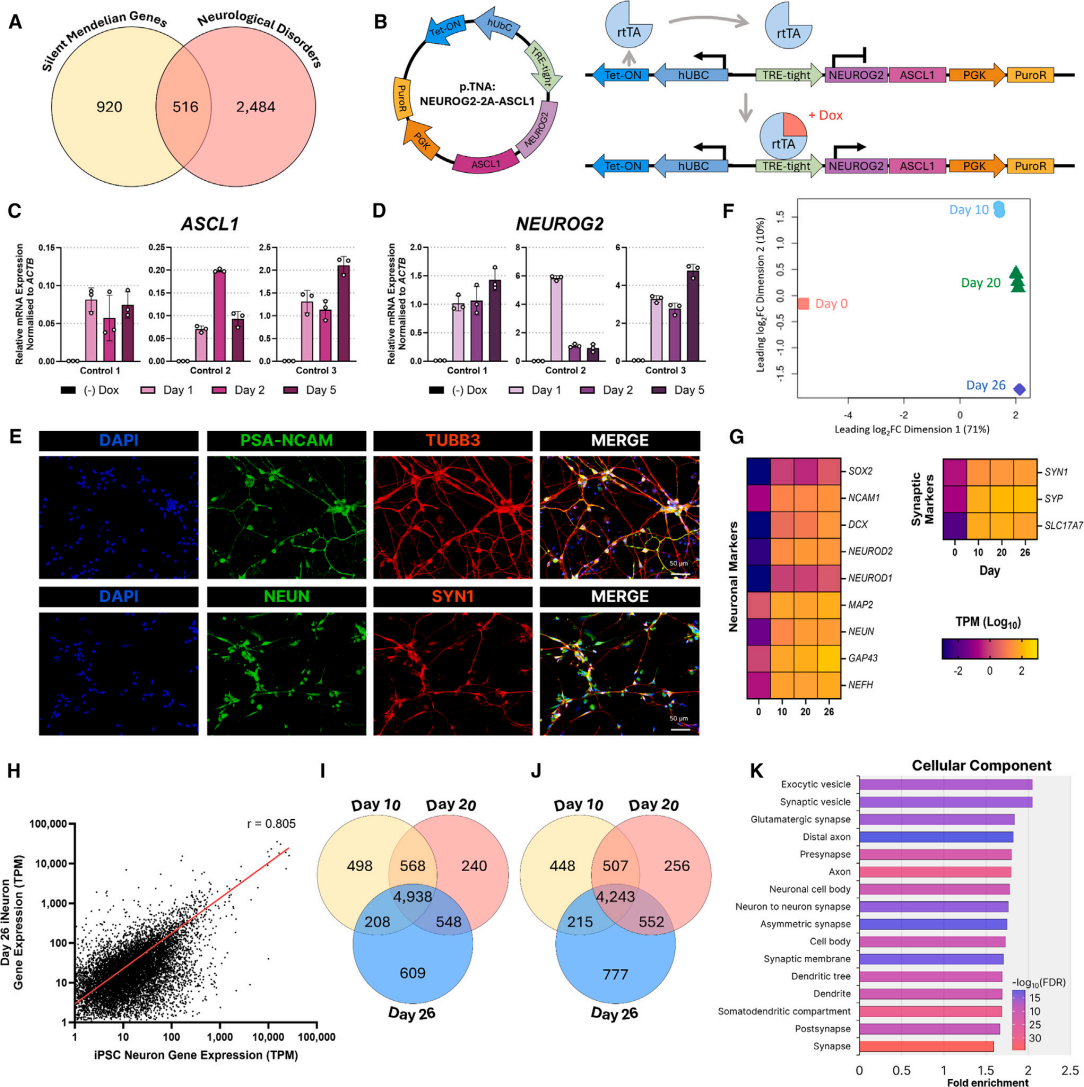
Transdifferentiation of HDFs directly into iNeurons (A) 516 neurological disorder genes are silent. A comparison between silent mendelian genes (SMGs) and a list of 3,000 neurological disorders reveals an overlap of 516 genes. These genes, herein referred to as silent neurological genes (SNGs), are not expressed at sufficient levels in CATs of blood, LCLs, or HDFs to enable analysis of mRNA splicing using srRNA-seq. (B) Schematic of the vector transgene featuring a Tet-On inducible promoter driving overexpression of *NEUROG2* and *ASCL1* (abbreviated as TNA). In the TNA transgene, the human ubiquitin C (hUbC) promoter drives the expression of Tet-ON encoding the reverse tetracycline-controlled transactivator (rtTA). rtTA binds the TRE-tight promoter when in the presence of doxycycline thus inducing NGN2 and ASCL1 expression. The phosphoglycerate kinase promoter (PGK) drives the constitutive expression of a puromycin-resistance cassette. The TNA transgene can be packaged into lentivirus. (C and D) Fibroblasts transduced with TNA overexpress *NERUOG2* and *ASCL1* in response to doxycycline treatment. RT-qPCR performed on RNA isolated from 3 control HDF lines transduced with TNA and treated with or without 2 μg/mL doxycycline (dox) for 1, 2, and 5 days (C) *ASCL1* expression and (D) *NEUROG2* expression. Error bars respresent standard deviation. (E) iNeurons display overt neuronal morphology and express a set of neuronal marker proteins. Immunofluorescent imaging of day 22 control iNeurons: PSA-NCAM (green), TUBB3 (red), NeuN (green), SYN1 (red), DAPI (blue). Scale bars, 50 μm. (F) Principal component analysis (PCA) of srRNA-seq. RNA was collected at day 0, 10, 20, and 26 of transdifferentiation. Experiment done in quadruplicate. Note that ∼70% of the transcriptional variance occurs by day 10 of transdifferentiation. (G) The srRNA-seq analysis reveals that iNeurons express cohorts of neuronal cell and synapse marker genes. Expression is reported as TPM (log_10_). (H) The transcriptional profile of iNeurons correlates with iPSC-derived neurons. The expression of genes (>1 TPM, *n* = 11,119 genes) was correlated between iNeurons (day 26, *n* = 4) and iPSC-derived neurons (day 90 of iPSC neuronal differentiation, *n* = 1) using Pearson’s correlation (r = 0.805, *p* < 0.0001). (I and J) Differential gene expression analysis of iNeuron transdifferentiation. The srRNA-seq transdifferentiation data were used to identify differentially expressed genes between HDFs (day 0) and other time points (day 10, 20, and 26) during transdifferentiation and the overlapping genes of each comparison identified. (I) Comparison of upregulated genes. (J) Comparison of downregulated genes. (K) Gene ontology analysis of genes upregulated in iNeurons at day 26 of transdifferentiation performed using ShinyGO 0.77. The highest-ranking GO terms are reported as fold enrichment and the FDR (-log_10_FDR).

**Figure 6:**
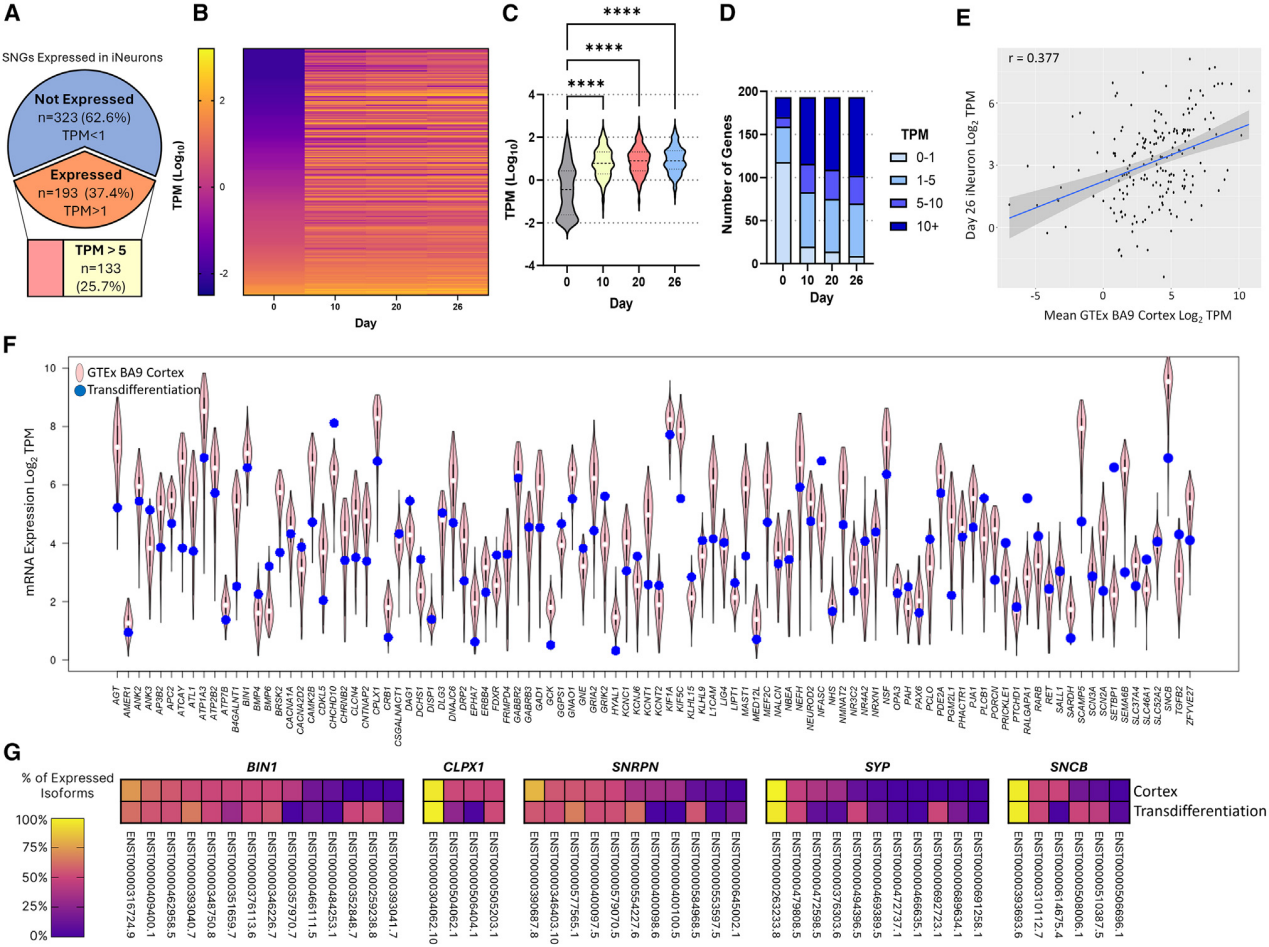
Expression of SNGs in iNeurons (A) 193 SNGs are expressed during the transdifferentiation of HDFs to iNeurons. Querying the list differentially expressed genes identified in cells undergoing transdifferentiation at days 10, 20, and 26 reveals that 193 of the 516 SNG genes are upregulated in iNeurons and with expression >1 TPM in at least one time point analyzed, with 133 of these displaying TPM >5. (B–D) Expression of the 193 SNGs during transdifferentiation of HDFs to iNeurons. (B) Heatmap shows expression of individual genes reported as TPM (log_10_). (C) Violin plots show significant upregulation of the cohort of 193 SNGs. Expression is reported as the mean TPM from across all four replicates for each time point. Statistical analysis was determined by ordinary one-way ANOVA with Tukey’s multiple comparison test. ^∗∗∗∗^*p* < 0.0001. (D) Categorization of the 193 genes as having expression within 0–1 TPM, 1–5 TPM, 5–10 TPM, and 10+ TPM, at each time point. (E) Correlation of expression (TPM) between the 193 iNeuron expressed SNGs at day 26 transdifferentiation and mean expression (TPM) in the human adult frontal cortex. (F) Comparison of expression of 100 of the 193 iNeuron-expressed SNGs with range of expression observed in the adult frontal cortex samples. (G) Comparison of the isoform diversity between SNGs expressed in iNeurons to that of the adult frontal cortex. Adult frontal cortex expression data were extracted from the GTEx database Version 8.

While SMGs are relevant to a range of disorders manifesting in different organ systems, there is a prominent association of SMGs with disorders of the nervous system (Figures 1 and S1). We cross referenced the list of SMGs with a combined list of 3,000 neurological disorder genes (combining Intellectual Disability and Progressive Neurological Disease Gene Pannels, Pannel App Australia) to define a list of 516 silent neurological genes (SNGs), equating to more than a third of SMGs (Figure 5A; Table S12). Gene ontology analysis revealed that SNGs are enriched in synaptic functions and ion transport (Figure S19; Tables S13–S15). We reasoned that conversion of HDFs to a neuronal cell identity may induce the endogenous expression of many of the SNGs, providing a single approach to induce the expression of many SNGs. While the conversion of HDFs to neurons via an iPSC state is a potential avenue, we considered the current associated resource burden (time, cost, expertise) prohibitive for larger scale diagnostics. We thus investigated the approach of cell transdifferentiation, which facilitates the conversion of HDFs directly into a neuronal-cell-like identity (known as induced neurons, iNeurons), bypassing the need for an iPSC intermediate.^88^ As such, transdifferentiation is rapid, taking <1 month to generate iNeurons from HDFs. Transdifferentiation of HDFs into iNeurons is driven by the overexpression of a combination of pioneer master pro-neural transcription factors (for example, *POUF3F2* [MIM: 600494], *NEUROG2* [MIM: 606624], *ASCL1* [MIM: 100790], *MYT1L,* and others) and culture in media containing compounds known to drive the differentiation of pluripotent stem cells into neuronal cell fate (e.g., inhibitors of the transfroming growth factor β/bone morphogenetic protein signaling pathway).^88^ We adopted a transdifferentiation approach utilizing the Tet-On (doxycycline) inducible expression of *NEUROG2* and *ASCL1* (herein referred as TNA) encoded on a single lentiviral vector (Figure 5B).^53^ We transduced three control HDF lines and selected for cells harboring the TNA transgene with puromycin (Figure S20). Addition of doxycycline induced expression of *NEUROG2* and *ASCL1* in all lines as expected (Figures 5C and 5D). Initial transdifferentiation of HDF to iNeurons generated cells that displayed overt neuronal cell morphology and expressed a cohort of neuronal cell marker genes/proteins (MAP2, TUBB3, NEUN, NESTIN, TAU1, and SYN1) (Figure S20). We noted depletion of media nutrients (data not shown), and so we modified the protocol for lower density culture with increased media replenishment, the latter of which resulted in elevated expression of neuronal cell marker genes (Figure S20). We also extended the culture of iNeurons beyond the third week using a maturation media containing a cocktail of neurotrophic factors, including brain-derived neurotrophic factor and glial-derived neurotrophic factor.^53^ In summary, we generated iNeurons from HDFs as confirmed by their overt neuronal cell morphology and expression of several neuronal cell marker genes (Figures 5E and S20). To more extensively characterize the HDF-derived iNeurons, we conducted srRNA-seq using RNA collected from before (day 0), during (day 10), and following transdifferentiation (day 20) and maturation (day 26) of iNeurons in quadruplicate (Table S16). Principal component analysis revealed distinct transcriptional profiles of all timepoints with ∼70% of variance occurring during the first 10 days of transdifferentiation (Figure 5F). Analysis of neural cell marker gene expression confirmed induction of a host of neuronal cell and synapse genes (Figure 5G), and the transcriptome of iNeurons was found to correlate well with that of iPSC-derived cortical excitatory neurons (Pearson’s correlation r = 0.805, *p* < 0.0001) (Figure 5H). Compared to HDFs, ∼4,938 genes were upregulated, and ∼4,243 genes were down regulated in common across all iNeuron conversion time points (false discovery rate <0.05, log_2_ fold change > or <1.5) (Figures 5I and 5J). Gene ontology analysis of upregulated genes of day 26 iNeurons revealed enrichment of terms relating to neuronal cell development and synaptic functions (Figures 5K and S21; Tables S17–S19). All together, these further confirm the transdifferentiation of HDFs to neuronal-like cells. We therefore proceeded to investigate how many of the SNGs are expressed in iNeurons. The analysis found that 193/516 SNGs (37.4%) were both differentially upregulated compared to HDFs and expressed at an abundance of at least one TPM during at least one time point analyzed (163 are common to all), with median expressions of 7.73, 9.05, and 9.78 TPM at days 10, 20, and 26 respectively (Figures 6A–6D; Table S20). We again set out to gauge how many of the 193 SNGs expressed in iNeurons displayed expression levels conducive to downstream srRNA-seq-based assessment of splicing. We first assessed if the exon junction read counts for SNGs satisfied the MRSD parameters used to define suitability for RNA-seq-based assessment of splicing (i.e., genes with a minimum of eight junction reads across 75% of junctions). For this, we analyzed a subset of 30 SNGs with diverse TPMs in iNeurons (<5 TPM, *n* = 10; >5 and <10 TPM, *n* = 10; and >10 TPM, *n* = 10) (Table S21). We found 27/30 of the selected SNGs satisfied these criteria (Figure S22; Table S21). We again found that the median number of exon junction reads correlated with TPMs across the 30 SNGs (Pearson’s correlation r = 0.89, *p* = 4.737e-11), encouraging us to utilize TPMs in reference to RNA diagnostic benchmarking studies^11^ (Figure S22). Of the SNGs expressed in iNeurons, we found 133/193 (69%) had TPM > 5, suggesting suitability for srRNA-seq-based assessment of RNA splicing, with the remainder likely requiring RT-PCR or deeper sequencing^11^ (Figure 6A). We then compared the expression of the 193 iNeuron-expressed SNGs to the adult frontal cortex (from GTEx) as the surrogate CRT, with the caveat that this may not be true for all genes in the cohort. The relative mRNA expression (TPM) of the 193 SNGs in iNeurons at day 26 of transdifferentiation was positively correlated with the relative mean expression (TPM) in the adult frontal cortex (Pearson’s correlation r = 0.377, *p* = 6.9 × 10^−8^) (Figure 6E) and most often fell within the range of expression observed across different adult cortex samples (Figures 6F and S23). Furthermore, the diversity of SNGs expressed isoforms in iNeurons was comparable to the isoform diversity observed in the frontal cortex (Figures 6G and S24). These data support transdifferentiation of HDFs to iNeurons as a rapid and robust avenue to induce the expression of 193 SNGs for the purpose of investigating SNG variants using HDFs derived from affected individuals, with investigation of variants in 133 such SNGs likely achievable using srRNA-seq.

### Investigating variants in SNGs using transdifferentiation

**Figure 7:**
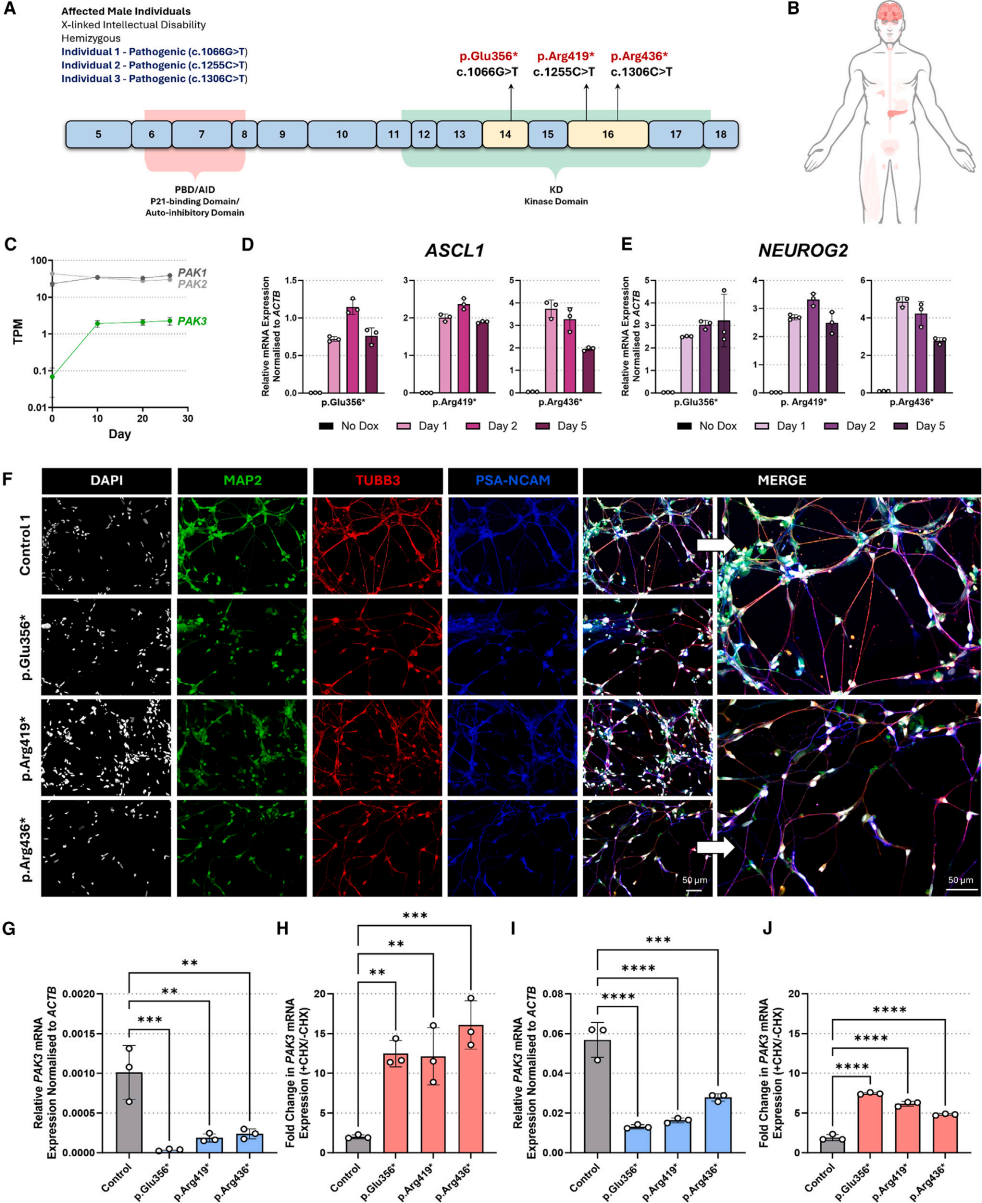
Pathological mechanisms of *PAK3* variants resolved using HDF transdifferentiation (A) Diagram of *PAK3* cDNA (GenBank: NM_002578.5) and encoded protein structure. *PAK3* nonsense variants are in exons 14 and 16, which encode the kinase domain and are downstream of the auto-inhibitory domain. (B) *PAK3* expression is predominately limited to the brain and pancreas (red) data from The HPA. (C) *PAK3* expression is upregulated during transdifferentiation. Expression of RAC1 family of activated kinases, *PAK1*, *PAK2*, and *PAK3*, during transdifferentiation of HDF to iNeurons. Expression data extracted from srRNA-seq (see Figure 5) and expressed as the mean TPM derived from four replicates per time point. (D and E) HDFs derived from individuals with the *PAK3* variants and transduced with TNA transgene overexpress *NERUOG2* and *ASCL1* in response to doxycycline treatment for 1, 2, and 5 days. Real-time qPCR assessment of (D) *ASCL1* mRNA expression and (E) *NEUROG2* mRNA expression. Expression is normalized to *ACTB* expression. (F) iNeurons transdifferentiated from HDFs derived from individuals with *PAK3* variants display overt neuronal morphology and express neuronal marker genes. Immunofluorescent imaging of day 22 iNeurons: MAP2 (green), TUBB3 (red), PSA-NCAM (blue), SYN1 (red), DAPI (white). Scale bars, 50 μm. (G) *PAK3* variant mRNA expression is reduced in iNeurons. Real-time qPCR of *PAK3* expression in day 22 control (*n* = 3) and nonsense variant iNeurons. Expression normalized to *ACTB*. (H) *PAK3* variant mRNA is subject to NMD. Real-time qPCR of *PAK3* mRNA expression in day 22 control (*n* = 3) and variant iNeurons treated with or without cycloheximide (CHX) for 24 h prior to RNA collection. mRNA expression is reported as the fold change in expression of CHX treated versus non-CHX treated cells. Expression normalized to *ACTB*. (I) *PAK3* variant mRNA is lowly expressed in transactivated HDFs. *PAK3* was transactivated in HDFs derived from *n* = 3 control individuals and individuals with PAK3 variants. Isolated RNA was subjected to real-time qPCR. Expression is normalized to ACTB. (J) *PAK3* variant mRNA acquired through transactivation is subject to NMD. Real-time qPCR of *PAK3* expression transactivated HDFs treated with or without cycloheximide (CHX) for 24 h prior to collection. Expression is reported as the fold change in expression of CHX-treated versus non-CHX-treated cells. Expression normalized to *ACTB*. Statistical analysis was determined by ordinary one-way ANOVA with Tukey’s multiple comparison test. Significance set as ^∗^*p* < 0.05, ^∗∗^ <0.01, ^∗∗∗^*p* < 0.001, and ^∗∗∗∗^*p* < 0.0001. All error bars respresent standard deviation.

To illustrate the potential of HDF transdifferentiation to iNeurons in the assessment of gene variants in SNGs, we investigated the role of NMD in the processing of a set of three nonsense variants found in an X chromosome intellectual disability gene, *PAK3*,^89^^,^^90^^,^^91^ discovered in male individuals with intellectual disability (MIM: 300558). The *PAK3* variants included (NM_002578.5, NP_002569.1): c.1066G>T (p.Glu356^∗^), c.1255C>T (p.Arg419^∗^), and c.1306C>T (p.Arg436^∗^) (Figure 7A). While these variants are classified as pathogenic based on DNA sequence alone, the mechanism of pathogenicity remains uncertain because *PAK3* is predominantly expressed in brain, pancreas, and other secretory glands, and as such, its mRNA is unavailable for study (Figures 7B and S25).^92^ It is predicted that the nonsense variant mRNAs are degraded by NMD and hence act via loss-of-function mechanism. However, the DNA-based rules governing whether an mRNA is subjected to NMD remain uncertain with many exceptions documented.^29^^,^^30^^,^^31^^,^^32^^,^^33^^,^^34^^,^^35^^,^^36^^,^^37^ If these nonsense *PAK3* mRNAs were to escape NMD, then the encoded truncated protein would lack its kinase domain and encode a protein consisting only of its inhibitory domain, with potential to also inhibit PAK3 heterodimeric partners such as that encoded by *PAK1* (MIM: 602590), a gene for which haploinsufficiency also causes intellectual disability (MIM: 618158).^93^ Referencing an scRNA-seq gene expression atlas of the human brain revealed that *PAK3* was highly expressed in neuronal cell populations (Figure S25). Likewise, we found *PAK3* robustly expressed in iNeurons along with *PAK1* and *PAK2* (MIM: 605022) (Figure 7C). We therefore investigated the role of NMD in the processing of nonsense *PAK3* mRNAs using transdifferentiation of HDFs derived from each of the three affected individuals. The *PAK3* variant HDFs, alongside three male control HDFs were engineered to harbor the TNA transgene, and all cell lines expressed transgenic *NEUROG2* and *ASCL1* in response to doxycycline (Figures 5C, 5D, 7D, and 7E). Following transdifferentiation, all cell lines displayed overt neuronal cell morphology (Figure S26) and expressed a range of neuronal cell marker genes and/or proteins without significant difference between *PAK3* and controls (MAP2, TUBB3, polysialylated (PSA)-NCAM, SYN1, DCX, SOX2, and RBFOX3, also known as NeuN) (Figures 7F and S26). At day 21, parallel cultures were treated with or without CHX for 24 h to inhibit NMD and RNA isolated for real-time qPCR analysis of *PAK3* expression. Compared to controls, *PAK3* expression was reduced in all three *PAK3* iNeuron samples, which had *PAK3* nonsense variants (Figure 7G). While inhibition of NMD with CHX resulted in a 2-fold increase in *PAK3* expression in controls, it caused a 12– to 16-fold increase in *PAK3* expression in *PAK3* variant iNeurons (Figure 7H). We found analogous results using *PAK3* mRNA derived from gene transactivation in the same HDF samples (Figures 7I and 7J). These data align with robust degradation of *PAK3* nonsense variant mRNAs by NMD, and as such, supports a loss-of-function pathogenic mechanism. More generally, these data provide proof-of-principle support that iNeurons can be used to investigate the mechanism of SNG variant effect on mRNA processing.

## Discussion

This investigation revealed that one-third of Mendelian genes are not expressed at sufficient levels to functionally assess RNA variants in CATs of blood and skin using srRNA-seq (Figure 1). Of these SMGs, the largest proportion are SNGs. Patients with VUSs in such genes that require functional RNA studies to resolve pathogenicity often never receive a genetic diagnosis because the RNA is unobtainable without invasive procedures. Variants in these genes account for 22.2% of VUSs in ClinVar, currently equating to 283,353 individuals without a diagnosis, and continue to accumulate. We repurpose the technologies of gene transactivation (developed for functional genomics) and transdifferentiation (developed for cell therapy and disease modeling) approaches into functional RNA diagnostic capacities suitable for variants in SMGs and SNGs, respectively. These approaches induce the expression of SMGs and SNGs at endogenous gene loci in patient-derived cells using a comparatively small resource investment. These approaches have several attractive features for clinical implementation. (1) They are gene-centric rather than variant centric, meaning all variants in a gene can be assessed using the same technique. (2) They activate the endogenous genomic loci thereby permitting study of variant impact on multiple full-length RNA isoforms complete with all regulatory and structural features. (3) They enable assessment of variant impact in the context of the patient’s genetic and cellular background, which influences splicing, RNA processing, NMD, translation, X inactivation, dosage compensation, allelic-specific expression, and beyond. (4) They are highly adaptable, with transactivation easily tailored to target any SMG promoter by simple re-engineering of only the gRNA components and with transdifferentiation providing a single tool relevant to many SNGs. (5) They are cost and time efficient for analysis of many VUSs, with relatively low up-front costs to design and implement, diminishing costs with re-use and relatively short workflows of days (for transactivation) to weeks (for transdifferentiation) when compared to iPSC, mini-gene, or CRISPR-editing-based alternatives. These features are aligned with “on-demand” diagnostic applications, and with on-going development, these techniques have the potential for up-scaling as an “off-the-shelf” diagnostic product for routine use. In addition to the significance of a genetic diagnosis, which cannot be overstated,^94^^,^^95^^,^^96^^,^^97^^,^^98^^,^^99^ the transactivation and transdifferentiation approaches also unveil “variant treatability” by revealing the mechanisms of pathogenicity—how the variant RNA is physiologically spliced and processed. This knowledge is prerequisite for development of new clinical trials or therapeutics, for example, involving antisense oligonucleotides (ASOs) or nonsense readthrough therapies.^1^^,^^15^^,^^16^

In this study, we developed and utilized gene transactivation to resolve the impact of variants on RNA processing in *DMD*, *SCN1A*, *USH2A*, and *PAK3* and test the efficacy of gene transactivation across 40 SMGs in HDFs derived from both affected and healthy individuals (Figures 2–4). This third generation CRISPRa system is the most highly multiplexed of all transactivation systems to date, with superior transactivation ability shown in benchmarking studies.^100^ Another study recently reported the use of gene transactivation to study variants in two SMGs, *MPZ* (MIM: 159440) and *SPAST* (604277), using the second generation dCas9- VP64-p65-Rta (VPR) fusion system, which was first *in-vitro* transcribed to RNA and delivered via electroporation to HDFs.^101^ These studies converge to highlight the utility of transactivation for variants assessment in SMGs using independent approaches. The choice of which system to adopt by users should include considerations on the requirements of equipment, expertise, facilities, and regulatory compliances among others. Both systems utilize commercially available reagents (see subjects, material, and methods) and have rapid work flows once reagents are in hand. Our vector system uses lentiviral delivery of transgenes and is therefore compatible with both short transient transactivation studies and creation of stable cell lines to permit ongoing studies, e.g., looking at variant impact on protein or cellular functions or testing of therapeutics such as ASOs. We exploited this feature to make stable transgenic HDFs for the purpose of testing the ability of 160 gRNAs to transactivate across 40 SMGs (Figure 2). These initial screens involving either scRNA-seq or srRNA-seq approaches revealed that transactivation can be gRNA, gene, and cell-type specific. The screening experiments suggested ∼35%–75% of SMGs were amenable to transactivation, depending on cell type (HEK293T and HDFs, respectively). These screens were, by design, tailored toward testing large numbers of SMGs at the expense of high levels of gRNA expression. Given these data suggested that those gene transactivation levels were related to gRNA expression level, the screens likely returned many false negative results. This encouraged us to extensively test the transactivation of SMGs one gene at a time, with enhanced gRNA expression, achieved by both optimizing efficient transient lentiviral delivery of the transgenes to HDFs and co-expressing all four gRNAs per gene simultaneously (Figure 3). Of the 20 SMGs we tested, all showed some level of transactivation in HDFs (and HEK293Ts), ranging from a 1 to 3,387 TPM, with 90% of SMGs tested >5 TPM and hence likely amenable for splice variant analysis using srRNA-seq.^11^ In most cases, the level of transactivation and isoform diversity was akin to the expression of the gene in its CRT. We observed preservation of reference splicing events in transactivated genes, aligned with previous studies highlighting HDFs as the best performing surrogate CAT for analysis of splicing of genes from inaccessible CRTs.^76^^,^^85^ Indeed, our investigation of the neurological genes that are expressed in HDFs shows that ∼90% of them are spliced in HDFs as they are in the cerebral cortex. Inevitably some splicing events exist in genes that are reliant on tissue-specific splicing factors absent in HDFs, which transactivated genes will be unable to model. While such tissue-specific splicing events are relatively rare among all splicing events, it remains an innate limitation of the approach, and a common limitation to all current gold-standard RNA-based diagnostics using CATs as surrogates for CRTs.^102^^,^^103^ We recommend management of this limitation: for each variant studied, an investigation should first consider if the splicing event of interest is conserved between the CAT being used and the CRT in control samples. If this event is not conserved in controls, then the assay is not suitable. If the event is conserved in controls, then this supports that any deviation from the canonical event is meaningful to pursue.

Why some genes were more conducive to transactivation than others remains an open question. In general, we observed that most strongly transactivated genes had open chromatin regions around the promoter (e.g., *PCDH19*), but this relationship was not definitive with some genes with closed chromatin achieving strong transactivation (e.g., *PTH* [MIM: 168450]) and some genes with open chromatin displaying weak transactivation (e.g., *DMD*). It has been shown by others, and within our own data (Figure 2), that different gRNAs can have different potencies and that different combinations of gRNAs when multiplexed can have divergent synergistic properties.^82^ We did not empirically optimize the best gRNA combinations, instead prioritizing a streamlined workflow, which nonetheless supports that rational selection of the four gRNAs per gene used in combination with the dCas9-Suntag system works robustly for most genes (Figure 3). This four-gRNA multiplex regime provides both opportunity for synergistic activity, and insurance against the inefficient actions of one (or more) gRNAs selected. For one gene with weak transactivation, *MYT1L*, we redesigned the gRNAs but failed to improve the outcome, while for another, *DMD*, redesigned gRNAs improved the outcome. The design and testing of gRNAs at a genome-wide scale continues to evolve, and with this comes resources to better aid gRNA selection.^104^^,^^105^ We also observed that cell type influences the ability to transactivate genes. For example, in the single guide screens, *COL2A1* transactivation was specific for HEK293T cells, while *SETBP1* (MIM: 611060) transactivation was better in HDFs. Thus, some genes may benefit from transactivation in alternative CAT-derived cell lines such as LCLs, T cells, or urothelial cells for which lentiviral delivery of transgenes has been demonstrated.^50^^,^^106^ Finally, the transactivation elements of the dCas9-ST-PH themselves can be modified. Our system utilized the p65-HSF hybrid transcriptional activator, but a given gene may respond better to a different transcriptional activator (e.g., VPR), or epigenetic modifier (e.g., Tet family of DNA demethylases or histone modifiers) or combinations therein.^78^^,^^107^^,^^108^^,^^109^ An excellent feature of the dCas9-ST system is that such elements are easily interchanged and can even be combined, as each dCas9-ST molecule has 10 docking sites for which to recruit any variety of co-expressed activators or epigenetic modifiers alone or in combination.^108^^,^^109^ Nonetheless, while the challenging examples draw opportunity for ongoing development, the current approach we tested across 40 SMGs, including 20 SMGs in great depth, works efficiently for its purpose of generating RNA from SMGs for diagnostic purposes.

Given that more than a third of SMGs were SNGs, contributing more than any other disease classification, we reasoned that HDF transdifferentiation to iNeurons would be a viable solution to induce expression of a large proportion of SMGs with a single method. Similar transdifferentiation approaches have been used for the study of muscle specific genes through *MYOD1* (MIM: 159970)-based transdifferentiation of HDFs to myoblasts.^110^ Of note, the application of HDF transdifferentiation to iNeurons for the purpose of resolving RNA variants in SNGs was also reported during the review of this study and warrants attention.^111^ We generated iNeurons that expressed a host of neuronal cell marker genes and showed transcriptional correlation to excitatory neurons produced from iPSCs (Figure 5). The iNeurons expressed 193/1436 (13.4%) of all SMGs, or 193/516 (37.4%) of the SNGs, at >1 TPM, with median TPMs ranging from 7.7 to 9.8 TPMs depending on time point analyzed (Figure 6). Most of these genes (162) are common to all timepoints. Of the 193 SNGs expressed in iNeurons, 133 had a TPM >5 at at least one time point, suggesting sufficient expression to enable the analysis of a splice variant by srRNA-seq, while the remainder (TPM between >1 and <5) would likely require RT-PCR-based analysis or deeper sequencing (i.e., beyond 80 million reads used in this study).^11^

Given that the PCA analysis of transcriptomes during iNeuron transdifferentiation also revealed that the largest variance in the data (70% of all) occurs during the first 10 days, for many genes, a truncated time course (10 days or even less) may be sufficient to detected robust SNG expression. This rapid reshaping of the transcriptome is aligned with the known roles of pioneer transcription factors *ASCL1* and *NEUROG2*, which sit at the apex of a neuronal transcription factor hierarchical network to collectively activate many neuronal genes.^112^^,^^113^ This method is conducive to ongoing development, whether relating to increased efficiency of iNeuron conversion (∼40%–60%^53^), iNeuron purification (e.g., FACS using cell surface markers PSA-NCAM^53^) or generating alternative target cell types (e.g., inhibitory neurons, dopaminergic neurons, astrocytes, or oligodendrocytes^88^^,^^114^^,^^115^^,^^116^^,^^117^) that can all contribute to accessing RNA from a larger number of SNGs from HDFs. In any case, the current optimized protocol derived in this study serves as a single-method solution to study the mechanism of variants in 193 SNGs (by srRNA-seq or RT-PCR), with its utility highlighted by the study of variants in *PAK3* (Figure 7). Furthermore, iNeurons go beyond diagnostic purposes to provide neuronal cell models of the affected individual. These can be used to further assess the impacts of such variants, and potentially treatments, at the level of neuronal cell function, which has already been shown for a host of other Mendelian and non-Mendelian neurological disorders.^118^^,^^119^^,^^120^^,^^121^

Collectively, our study demonstrates the utility of both gene transactivation and cell transdifferentiation to enable the study of RNA from SMGs and SNGs, respectively. The combined potential of these approaches may provide access to RNA from almost any SMG or SNG, with each method possessing scope for ongoing improvement and development. Further studies using these platforms will benchmark their utility in years to come, including important comparisons to RNA extracted from CRTs and other models such as iPSC differentiation. These initial studies reveal potential benefits to the many individuals with variants in SMGs living without a diagnosis and enduring the burdens of a diagnostic odyssey and/or lack of precision treatments.

## Consortia

The PERSYST Investigator Team: Dimitar N. Azmanov, Christopher P. Barnett, Simon C. Barry, Gareth Baynam, Samuel F. Berkovic, John Christodoulou, David J. Coman, Sandra Cooper, Mark A. Corbett, Martin Delatycki, Tracy E. Dudding, Sue Fletcher, Alison E. Gardner, Jozef Gecz, Megan J Higgins, Michael S. Hildebrand, Lachlan A. Jolly, Ryan Lister, Julie McGaughran, Christian Pflueger, Cathryn Poulton, Tony Roscioli, Ingrid Scheffer Hamish S. Scott, Andrew H. Sinclair, Amanda B. Spurdle, Tiong Y. Tan, Clare L. van Eyk, and Irina Voineagu.

See also supplemental information.

## Data and code availability

All data needed to evaluate the conclusions in the paper are present in the paper and/or the supplemental information. The RNA sequencing data from iNeurons has been deposited in NCBI’s Gene Expression Omnibus (GEO): GSE272900. All other RNA sequencing data are available upon request and if in line with the written informed consents provided by the affected individuals or their legal guardians.

## Web resources

E-CRISP, http://www.e-crisp.org/E-CRISP/
FANTOM ZENBU, https://fantom.gsc.riken.jp/zenbu/
GTEx portal browser, https://www.gtexportal.org/home/
The Human Protein Atlas, https://www.proteinatlas.org/
MAJIQ-CAT, https://tools.biociphers.org/majiq-cat/
MRSD Web Portal, https://mcgm-mrsd.github.io/
PanelApp Australia, https://panelapp.agha.umccr.org/
UCSC Genome Browser, https://genome.ucsc.edu/

## Supplemental information

Document S1. Figures S1–S26, PERSYST Investigator Team affiliations, and acknowledgments

Data S1. Tables S1–S21

Document S2. Article plus supplemental information

## References

[1] Boycott K.M., Hartley T., Biesecker L.G., Gibbs R.A., Innes A.M., Riess O., Belmont J., Dunwoodie S.L., Jojic N., Lassmann T. (2019). A Diagnosis for All Rare Genetic Diseases: The Horizon and the Next Frontiers. Cell.

[2] Brnich S.E., Abou Tayoun A.N., Couch F.J., Cutting G.R., Greenblatt M.S., Heinen C.D., Kanavy D.M., Luo X., McNulty S.M., Starita L.M. (2019). Recommendations for application of the functional evidence PS3/BS3 criterion using the ACMG/AMP sequence variant interpretation framework. Genome Med..

[3] Richards S., Aziz N., Bale S., Bick D., Das S., Gastier-Foster J., Grody W.W., Hegde M., Lyon E., Spector E. (2015). Standards and guidelines for the interpretation of sequence variants: a joint consensus recommendation of the American College of Medical Genetics and Genomics and the Association for Molecular Pathology. Genet. Med..

[4] Consortium G.T. (2020). The GTEx Consortium atlas of genetic regulatory effects across human tissues. Science.

[5] Abdellaoui A., Yengo L., Verweij K.J.H., Visscher P.M. (2023). 15 years of GWAS discovery: Realizing the promise. Am. J. Hum. Genet..

[6] Nguyen L.S., Wilkinson M.F., Gecz J. (2014). Nonsense-mediated mRNA decay: inter-individual variability and human disease. Neurosci. Biobehav. Rev..

[7] Ma Z., Zhu P., Shi H., Guo L., Zhang Q., Chen Y., Chen S., Zhang Z., Peng J., Chen J. (2019). PTC-bearing mRNA elicits a genetic compensation response via Upf3a and COMPASS components. Nature.

[8] Consortium G.T. (2013). The Genotype-Tissue Expression (GTEx) project. Nat. Genet..

[9] Shvetsova E., Sofronova A., Monajemi R., Gagalova K., Draisma H.H.M., White S.J., Santen G.W.E., Chuva de Sousa Lopes S.M., Heijmans B.T., van Meurs J. (2019). Skewed X-inactivation is common in the general female population. Eur. J. Hum. Genet..

[10] Truty R., Ouyang K., Rojahn S., Garcia S., Colavin A., Hamlington B., Freivogel M., Nussbaum R.L., Nykamp K., Aradhya S. (2021). Spectrum of splicing variants in disease genes and the ability of RNA analysis to reduce uncertainty in clinical interpretation. Am. J. Hum. Genet..

[11] Bournazos A.M., Riley L.G., Bommireddipalli S., Ades L., Akesson L.S., Al-Shinnag M., Alexander S.I., Archibald A.D., Balasubramaniam S., Berman Y. (2022). Standardized practices for RNA diagnostics using clinically accessible specimens reclassifies 75% of putative splicing variants. Genet. Med..

[12] Maddirevula S., Kuwahara H., Ewida N., Shamseldin H.E., Patel N., Alzahrani F., AlSheddi T., AlObeid E., Alenazi M., Alsaif H.S. (2020). Analysis of transcript-deleterious variants in Mendelian disorders: implications for RNA-based diagnostics. Genome Biol..

[13] Baralle D., Buratti E. (2017). RNA splicing in human disease and in the clinic. Clin. Sci..

[14] Mort M., Ivanov D., Cooper D.N., Chuzhanova N.A. (2008). A meta-analysis of nonsense mutations causing human genetic disease. Hum. Mutat..

[15] Mittal S., Tang I., Gleeson J.G. (2022). Evaluating human mutation databases for "treatability" using patient-customized therapy. Med.

[16] Pitout I., Flynn L.L., Wilton S.D., Fletcher S. (2019). Antisense-mediated splice intervention to treat human disease: the odyssey continues. F1000Res..

[17] Keeling K.M., Xue X., Gunn G., Bedwell D.M. (2014). Therapeutics based on stop codon readthrough. Annu. Rev. Genomics Hum. Genet..

[18] Caminsky N., Mucaki E.J., Rogan P.K. (2014). Interpretation of mRNA splicing mutations in genetic disease: review of the literature and guidelines for information-theoretical analysis. F1000Res..

[19] Teraoka S.N., Telatar M., Becker-Catania S., Liang T., Onengut S., Tolun A., Chessa L., Sanal O., Bernatowska E., Gatti R.A., Concannon P. (1999). Splicing defects in the ataxia-telangiectasia gene, ATM: underlying mutations and consequences. Am. J. Hum. Genet..

[20] Ars E., Serra E., Garcia J., Kruyer H., Gaona A., Lazaro C., Estivill X. (2000). Mutations affecting mRNA splicing are the most common molecular defects in patients with neurofibromatosis type 1. Hum. Mol. Genet..

[21] Soemedi R., Cygan K.J., Rhine C.L., Wang J., Bulacan C., Yang J., Bayrak-Toydemir P., McDonald J., Fairbrother W.G. (2017). Pathogenic variants that alter protein code often disrupt splicing. Nat. Genet..

[22] Wimmer K., Schamschula E., Wernstedt A., Traunfellner P., Amberger A., Zschocke J., Kroisel P., Chen Y., Callens T., Messiaen L. (2020). AG-exclusion zone revisited: Lessons to learn from 91 intronic NF1 3' splice site mutations outside the canonical AG-dinucleotides. Hum. Mutat..

[23] Cygan K.J., Sanford C.H., Fairbrother W.G. (2017). Spliceman2: a computational web server that predicts defects in pre-mRNA splicing. Bioinformatics.

[24] Jian X., Boerwinkle E., Liu X. (2014). In silico prediction of splice-altering single nucleotide variants in the human genome. Nucleic Acids Res..

[25] Naito T. (2019). Predicting the impact of single nucleotide variants on splicing via sequence-based deep neural networks and genomic features. Hum. Mutat..

[26] Jaganathan K., Kyriazopoulou Panagiotopoulou S., McRae J.F., Darbandi S.F., Knowles D., Li Y.I., Kosmicki J.A., Arbelaez J., Cui W., Schwartz G.B. (2019). Predicting Splicing from Primary Sequence with Deep Learning. Cell.

[27] Danis D., Jacobsen J.O.B., Carmody L.C., Gargano M.A., McMurry J.A., Hegde A., Haendel M.A., Valentini G., Smedley D., Robinson P.N. (2021). Interpretable prioritization of splice variants in diagnostic next-generation sequencing. Am. J. Hum. Genet..

[28] Dawes R., Bournazos A.M., Bryen S.J., Bommireddipalli S., Marchant R.G., Joshi H., Cooper S.T. (2023). SpliceVault predicts the precise nature of variant-associated mis-splicing. Nat. Genet..

[29] Lindeboom R.G., Supek F., Lehner B. (2016). The rules and impact of nonsense-mediated mRNA decay in human cancers. Nat. Genet..

[30] MacArthur D.G., Balasubramanian S., Frankish A., Huang N., Morris J., Walter K., Jostins L., Habegger L., Pickrell J.K., Montgomery S.B. (2012). A systematic survey of loss-of-function variants in human protein-coding genes. Science.

[31] Miller J.N., Pearce D.A. (2014). Nonsense-mediated decay in genetic disease: friend or foe?. Mutat. Res. Rev. Mutat. Res..

[32] Rivas M.A., Pirinen M., Conrad D.F., Lek M., Tsang E.K., Karczewski K.J., Maller J.B., Kukurba K.R., DeLuca D.S., Fromer M. (2015). Human genomics. Effect of predicted protein-truncating genetic variants on the human transcriptome. Science.

[33] Buhler M., Paillusson A., Muhlemann O. (2004). Efficient downregulation of immunoglobulin mu mRNA with premature translation-termination codons requires the 5'-half of the VDJ exon. Nucleic Acids Res..

[34] Wang J., Gudikote J.P., Olivas O.R., Wilkinson M.F. (2002). Boundary-independent polar nonsense-mediated decay. EMBO Rep..

[35] Zhang J., Maquat L.E. (1996). Evidence that the decay of nucleus-associated nonsense mRNA for human triosephosphate isomerase involves nonsense codon recognition after splicing. RNA.

[36] Romao L., Inacio A., Santos S., Avila M., Faustino P., Pacheco P., Lavinha J. (2000). Nonsense mutations in the human beta-globin gene lead to unexpected levels of cytoplasmic mRNA accumulation. Blood.

[37] Silva A.L., Ribeiro P., Inacio A., Liebhaber S.A., Romao L. (2008). Proximity of the poly(A)-binding protein to a premature termination codon inhibits mammalian nonsense-mediated mRNA decay. RNA.

[38] Yepez V.A., Mertes C., Muller M.F., Klaproth-Andrade D., Wachutka L., Fresard L., Gusic M., Scheller I.F., Goldberg P.F., Prokisch H., Gagneur J. (2021). Detection of aberrant gene expression events in RNA sequencing data. Nat. Protoc..

[39] Cummings B.B., Marshall J.L., Tukiainen T., Lek M., Donkervoort S., Foley A.R., Bolduc V., Waddell L.B., Sandaradura S.A., O'Grady G.L. (2017). Improving genetic diagnosis in Mendelian disease with transcriptome sequencing. Sci. Transl. Med..

[40] Kremer L.S., Bader D.M., Mertes C., Kopajtich R., Pichler G., Iuso A., Haack T.B., Graf E., Schwarzmayr T., Terrile C. (2017). Genetic diagnosis of Mendelian disorders via RNA sequencing. Nat. Commun..

[41] Murdock D.R., Dai H., Burrage L.C., Rosenfeld J.A., Ketkar S., Muller M.F., Yepez V.A., Gagneur J., Liu P., Chen S. (2021). Transcriptome-directed analysis for Mendelian disease diagnosis overcomes limitations of conventional genomic testing. J. Clin. Invest..

[42] Lee H., Huang A.Y., Wang L.K., Yoon A.J., Renteria G., Eskin A., Signer R.H., Dorrani N., Nieves-Rodriguez S., Wan J. (2020). Diagnostic utility of transcriptome sequencing for rare Mendelian diseases. Genet. Med..

[43] Gonorazky H.D., Naumenko S., Ramani A.K., Nelakuditi V., Mashouri P., Wang P., Kao D., Ohri K., Viththiyapaskaran S., Tarnopolsky M.A. (2019). Expanding the Boundaries of RNA Sequencing as a Diagnostic Tool for Rare Mendelian Disease. Am. J. Hum. Genet..

[44] Wai H.A., Lord J., Lyon M., Gunning A., Kelly H., Cibin P., Seaby E.G., Spiers-Fitzgerald K., Lye J., Ellard S. (2020). Blood RNA analysis can increase clinical diagnostic rate and resolve variants of uncertain significance. Genet. Med..

[45] Fraile-Bethencourt E., Diez-Gomez B., Velasquez-Zapata V., Acedo A., Sanz D.J., Velasco E.A. (2017). Functional classification of DNA variants by hybrid minigenes: Identification of 30 spliceogenic variants of BRCA2 exons 17 and 18. PLoS Genet..

[46] Carvill G.L., Engel K.L., Ramamurthy A., Cochran J.N., Roovers J., Stamberger H., Lim N., Schneider A.L., Hollingsworth G., Holder D.H. (2018). Aberrant Inclusion of a Poison Exon Causes Dravet Syndrome and Related SCN1A-Associated Genetic Epilepsies. Am. J. Hum. Genet..

[47] Prasuhn J., Martensson C.U., Krajka V., Klein C., Rakovic A. (2017). Genome-Edited, TH-expressing Neuroblastoma Cells as a Disease Model for Dopamine-Related Disorders: A Proof-of-Concept Study on DJ-1-deficient Parkinsonism. Front. Cell. Neurosci..

[48] Brooks I.R., Garrone C.M., Kerins C., Kiar C.S., Syntaka S., Xu J.Z., Spagnoli F.M., Watt F.M. (2022). Functional genomics and the future of iPSCs in disease modeling. Stem Cell Rep..

[49] Ran F.A., Hsu P.D., Wright J., Agarwala V., Scott D.A., Zhang F. (2013). Genome engineering using the CRISPR-Cas9 system. Nat. Protoc..

[50] Jolly L.A., Sun Y., Carroll R., Homan C.C., Gecz J. (2018). Robust imaging and gene delivery to study human lymphoblastoid cell lines. J. Hum. Genet..

[51] Jolly L.A., Homan C.C., Jacob R., Barry S., Gecz J. (2013). The UPF3B gene, implicated in intellectual disability, autism, ADHD and childhood onset schizophrenia regulates neural progenitor cell behaviour and neuronal outgrowth. Hum. Mol. Genet..

[52] Johnson B.V., Kumar R., Oishi S., Alexander S., Kasherman M., Vega M.S., Ivancevic A., Gardner A., Domingo D., Corbett M. (2020). Partial Loss of USP9X Function Leads to a Male Neurodevelopmental and Behavioral Disorder Converging on Transforming Growth Factor beta Signaling. Biol. Psychiatry.

[53] Zhou-Yang L., Eichhorner S., Karbacher L., Bohnke L., Traxler L., Mertens J. (2021). Direct Conversion of Human Fibroblasts to Induced Neurons. Methods Mol. Biol..

[54] Kim D., Paggi J.M., Park C., Bennett C., Salzberg S.L. (2019). Graph-based genome alignment and genotyping with HISAT2 and HISAT-genotype. Nat. Biotechnol..

[55] Patro R., Duggal G., Love M.I., Irizarry R.A., Kingsford C. (2017). Salmon provides fast and bias-aware quantification of transcript expression. Nat. Methods.

[56] Robinson M.D., McCarthy D.J., Smyth G.K. (2010). edgeR: a Bioconductor package for differential expression analysis of digital gene expression data. Bioinformatics.

[57] Smedley D., Haider S., Ballester B., Holland R., London D., Thorisson G., Kasprzyk A. (2009). BioMart--biological queries made easy. BMC Genom..

[58] Liao Y., Smyth G.K., Shi W. (2019). The R package Rsubread is easier, faster, cheaper and better for alignment and quantification of RNA sequencing reads. Nucleic Acids Res..

[59] Li H. (2018). Minimap2: pairwise alignment for nucleotide sequences. Bioinformatics.

[60] Corces M.R., Trevino A.E., Hamilton E.G., Greenside P.G., Sinnott-Armstrong N.A., Vesuna S., Satpathy A.T., Rubin A.J., Montine K.S., Wu B. (2017). An improved ATAC-seq protocol reduces background and enables interrogation of frozen tissues. Nat. Methods.

[61] Chen S., Zhou Y., Chen Y., Gu J. (2018). fastp: an ultra-fast all-in-one FASTQ preprocessor. Bioinformatics.

[62] Langmead B., Salzberg S.L. (2012). Fast gapped-read alignment with Bowtie 2. Nat. Methods.

[63] Tange O. (2011). GNU Parallel: The command-line power tool. The USENIX Magazine.

[64] Consortium E.P., Moore J.E., Purcaro M.J., Pratt H.E., Epstein C.B., Shoresh N., Adrian J., Kawli T., Davis C.A., Dobin A. (2020). Expanded encyclopaedias of DNA elements in the human and mouse genomes. Nature.

[65] Li H., Handsaker B., Wysoker A., Fennell T., Ruan J., Homer N., Marth G., Abecasis G., Durbin R., Genome Project Data Processing S. (2009). The Sequence Alignment/Map format and SAMtools. Bioinformatics.

[66] Zhang Y., Liu T., Meyer C.A., Eeckhoute J., Johnson D.S., Bernstein B.E., Nusbaum C., Myers R.M., Brown M., Li W., Liu X.S. (2008). Model-based analysis of ChIP-Seq (MACS). Genome Biol..

[67] Quinlan A.R., Hall I.M. (2010). BEDTools: a flexible suite of utilities for comparing genomic features. Bioinformatics.

[68] Jolly L.A., Nguyen L.S., Domingo D., Sun Y., Barry S., Hancarova M., Plevova P., Vlckova M., Havlovicova M., Kalscheuer V.M. (2015). HCFC1 loss-of-function mutations disrupt neuronal and neural progenitor cells of the developing brain. Hum. Mol. Genet..

[69] Bohnke L., Zhou-Yang L., Pelucchi S., Kogler F., Frantal D., Schon F., Lagerstrom S., Borgogno O., Baltazar J., Herdy J.R. (2022). Chemical Replacement of Noggin with Dorsomorphin Homolog 1 for Cost-Effective Direct Neuronal Conversion. Cell Reprogram.

[70] Henrie A., Hemphill S.E., Ruiz-Schultz N., Cushman B., DiStefano M.T., Azzariti D., Harrison S.M., Rehm H.L., Eilbeck K. (2018). ClinVar Miner: Demonstrating utility of a Web-based tool for viewing and filtering ClinVar data. Hum. Mutat..

[71] Stenson P.D., Mort M., Ball E.V., Chapman M., Evans K., Azevedo L., Hayden M., Heywood S., Millar D.S., Phillips A.D., Cooper D.N. (2020). The Human Gene Mutation Database (HGMD®): optimizing its use in a clinical diagnostic or research setting. Hum. Genet..

[72] Rowlands C.F., Taylor A., Rice G., Whiffin N., Hall H.N., Newman W.G., Black G.C.M., kConFab I., O'Keefe R.T., Hubbard S. (2022). MRSD: A quantitative approach for assessing suitability of RNA-seq in the investigation of mis-splicing in Mendelian disease. Am. J. Hum. Genet..

[73] Ge S.X., Jung D., Yao R. (2020). ShinyGO: a graphical gene-set enrichment tool for animals and plants. Bioinformatics.

[74] Uhlen M., Fagerberg L., Hallstrom B.M., Lindskog C., Oksvold P., Mardinoglu A., Sivertsson A., Kampf C., Sjostedt E., Asplund A. (2015). Proteomics. Tissue-based map of the human proteome. Science.

[75] Forrest A.R., Kawaji H., Rehli M., Baillie J.K., de Hoon M.J., Haberle V., Lassmann T., FANTOM Consortium, the RIKEN PMI, CLST (DGT) (2014). A promoter-level mammalian expression atlas. Nature.

[76] Aicher J.K., Jewell P., Vaquero-Garcia J., Barash Y., Bhoj E.J. (2020). Mapping RNA splicing variations in clinically accessible and nonaccessible tissues to facilitate Mendelian disease diagnosis using RNA-seq. Genet. Med..

[77] Tanenbaum M.E., Gilbert L.A., Qi L.S., Weissman J.S., Vale R.D. (2014). A protein-tagging system for signal amplification in gene expression and fluorescence imaging. Cell.

[78] Pflueger C., Tan D., Swain T., Nguyen T., Pflueger J., Nefzger C., Polo J.M., Ford E., Lister R. (2018). A modular dCas9-SunTag DNMT3A epigenome editing system overcomes pervasive off-target activity of direct fusion dCas9-DNMT3A constructs. Genome Res..

[79] Kaplanis J., Samocha K.E., Wiel L., Zhang Z., Arvai K.J., Eberhardt R.Y., Gallone G., Lelieveld S.H., Martin H.C., McRae J.F. (2020). Evidence for 28 genetic disorders discovered by combining healthcare and research data. Nature.

[80] Sherry S.T., Ward M.H., Kholodov M., Baker J., Phan L., Smigielski E.M., Sirotkin K. (2001). dbSNP: the NCBI database of genetic variation. Nucleic Acids Res..

[81] Heigwer F., Kerr G., Boutros M. (2014). E-CRISP: fast CRISPR target site identification. Nat. Methods.

[82] Kabadi A.M., Ousterout D.G., Hilton I.B., Gersbach C.A. (2014). Multiplex CRISPR/Cas9-based genome engineering from a single lentiviral vector. Nucleic Acids Res..

[83] Replogle J.M., Norman T.M., Xu A., Hussmann J.A., Chen J., Cogan J.Z., Meer E.J., Terry J.M., Riordan D.P., Srinivas N. (2020). Combinatorial single-cell CRISPR screens by direct guide RNA capture and targeted sequencing. Nat. Biotechnol..

[84] Dixit A., Parnas O., Li B., Chen J., Fulco C.P., Jerby-Arnon L., Marjanovic N.D., Dionne D., Burks T., Raychowdhury R. (2016). Perturb-Seq: Dissecting Molecular Circuits with Scalable Single-Cell RNA Profiling of Pooled Genetic Screens. Cell.

[85] Wagner N., Celik M.H., Holzlwimmer F.R., Mertes C., Prokisch H., Yepez V.A., Gagneur J. (2023). Aberrant splicing prediction across human tissues. Nat. Genet..

[86] Aksentijevich I., Masters S.L., Ferguson P.J., Dancey P., Frenkel J., van Royen-Kerkhoff A., Laxer R., Tedgard U., Cowen E.W., Pham T.H. (2009). An autoinflammatory disease with deficiency of the interleukin-1-receptor antagonist. N. Engl. J. Med..

[87] Kielbasa S.M., Wan R., Sato K., Horton P., Frith M.C. (2011). Adaptive seeds tame genomic sequence comparison. Genome Res..

[88] Mertens J., Marchetto M.C., Bardy C., Gage F.H. (2016). Evaluating cell reprogramming, differentiation and conversion technologies in neuroscience. Nat. Rev. Neurosci..

[89] Allen K.M., Gleeson J.G., Bagrodia S., Partington M.W., MacMillan J.C., Cerione R.A., Mulley J.C., Walsh C.A. (1998). PAK3 mutation in nonsyndromic X-linked mental retardation. Nat. Genet..

[90] Bienvenu T., des Portes V., McDonell N., Carrie A., Zemni R., Couvert P., Ropers H.H., Moraine C., van Bokhoven H., Fryns J.P. (2000). Missense mutation in PAK3, R67C, causes X-linked nonspecific mental retardation. Am. J. Med. Genet..

[91] Duarte K., Heide S., Poea-Guyon S., Rousseau V., Depienne C., Rastetter A., Nava C., Attie-Bitach T., Razavi F., Martinovic J. (2020). PAK3 mutations responsible for severe intellectual disability and callosal agenesis inhibit cell migration. Neurobiol. Dis..

[92] Kreis P., Rousseau V., Thevenot E., Combeau G., Barnier J.V. (2008). The four mammalian splice variants encoded by the p21-activated kinase 3 gene have different biological properties. J. Neurochem..

[93] Combeau G., Kreis P., Domenichini F., Amar M., Fossier P., Rousseau V., Barnier J.V. (2012). The p21-activated kinase PAK3 forms heterodimers with PAK1 in brain implementing trans-regulation of PAK3 activity. J. Biol. Chem..

[94] Splinter K., Adams D.R., Bacino C.A., Bellen H.J., Bernstein J.A., Cheatle-Jarvela A.M., Eng C.M., Esteves C., Gahl W.A., Hamid R. (2018). Effect of Genetic Diagnosis on Patients with Previously Undiagnosed Disease. N. Engl. J. Med..

[95] Stavropoulos D.J., Merico D., Jobling R., Bowdin S., Monfared N., Thiruvahindrapuram B., Nalpathamkalam T., Pellecchia G., Yuen R.K.C., Szego M.J. (2016). Whole Genome Sequencing Expands Diagnostic Utility and Improves Clinical Management in Pediatric Medicine. NPJ Genom. Med..

[96] Zurynski Y., Deverell M., Dalkeith T., Johnson S., Christodoulou J., Leonard H., Elliott E.J., APSU Rare Diseases Impacts on Families Study group (2017). Australian children living with rare diseases: experiences of diagnosis and perceived consequences of diagnostic delays. Orphanet J. Rare Dis..

[97] Bhattacharya K., Millis N., Jaffe A., Zurynski Y. (2021). Rare diseases research and policy in Australia: On the journey to equitable care. J. Paediatr. Child Health.

[98] Tan T.Y., Dillon O.J., Stark Z., Schofield D., Alam K., Shrestha R., Chong B., Phelan D., Brett G.R., Creed E. (2017). Diagnostic Impact and Cost-effectiveness of Whole-Exome Sequencing for Ambulant Children With Suspected Monogenic Conditions. JAMA Pediatr..

[99] Stark Z., Tan T.Y., Chong B., Brett G.R., Yap P., Walsh M., Yeung A., Peters H., Mordaunt D., Cowie S. (2016). A prospective evaluation of whole-exome sequencing as a first-tier molecular test in infants with suspected monogenic disorders. Genet. Med..

[100] Zhou H., Liu J., Zhou C., Gao N., Rao Z., Li H., Hu X., Li C., Yao X., Shen X. (2018). In vivo simultaneous transcriptional activation of multiple genes in the brain using CRISPR-dCas9-activator transgenic mice. Nat. Neurosci..

[101] Terkelsen T., Mikkelsen N.S., Bak E.N., Vad-Nielsen J., Blechingberg J., Weiss S., Drue S.O., Andersen H., Andresen B.S., Bak R.O., Jensen U.B. (2024). CRISPR activation to characterize splice-altering variants in easily accessible cells. Am. J. Hum. Genet..

[102] Baralle F.E., Giudice J. (2017). Alternative splicing as a regulator of development and tissue identity. Nat. Rev. Mol. Cell Biol..

[103] Cheng J., Celik M.H., Kundaje A., Gagneur J. (2021). MTSplice predicts effects of genetic variants on tissue-specific splicing. Genome Biol..

[104] Sanson K.R., Hanna R.E., Hegde M., Donovan K.F., Strand C., Sullender M.E., Vaimberg E.W., Goodale A., Root D.E., Piccioni F., Doench J.G. (2018). Optimized libraries for CRISPR-Cas9 genetic screens with multiple modalities. Nat. Commun..

[105] Guna A., Page K.R., Replogle J.M., Esantsi T.K., Wang M.L., Weissman J.S., Voorhees R.M. (2023). A dual sgRNA library design to probe genetic modifiers using genome-wide CRISPRi screens. BMC Genom..

[106] Kim E.Y., Page P., Dellefave-Castillo L.M., McNally E.M., Wyatt E.J. (2016). Direct reprogramming of urine-derived cells with inducible MyoD for modeling human muscle disease. Skelet. Muscle.

[107] Swain T., Pflueger C., Freytag S., Poppe D., Pflueger J., Nguyen T.V., Li J.K., Lister R. (2024). A modular dCas9-based recruitment platform for combinatorial epigenome editing. Nucleic Acids Res..

[108] Morita S., Horii T., Hatada I. (2023). Regulation of Gene Expression Using dCas9-SunTag Platforms. Methods Mol. Biol..

[109] Morita S., Horii T., Kimura M., Hatada I. (2020). Synergistic Upregulation of Target Genes by TET1 and VP64 in the dCas9-SunTag Platform. Int. J. Mol. Sci..

[110] Battistelli C., Garbo S., Maione R. (2022). MyoD-Induced Trans-Differentiation: A Paradigm for Dissecting the Molecular Mechanisms of Cell Commitment, Differentiation and Reprogramming. Cells.

[111] Li S., Zhao S., Sinson J.C., Bajic A., Rosenfeld J.A., Neeley M.B., Pena M., Worley K.C., Burrage L.C., Weisz-Hubshman M. (2024). The clinical utility and diagnostic implementation of human subject cell transdifferentiation followed by RNA sequencing. Am. J. Hum. Genet..

[112] Dennis D.J., Han S., Schuurmans C. (2019). bHLH transcription factors in neural development, disease, and reprogramming. Brain Res..

[113] Barral A., Zaret K.S. (2024). Pioneer factors: roles and their regulation in development. Trends Genet..

[114] Quist E., Trovato F., Avaliani N., Zetterdahl O.G., Gonzalez-Ramos A., Hansen M.G., Kokaia M., Canals I., Ahlenius H. (2022). Transcription factor-based direct conversion of human fibroblasts to functional astrocytes. Stem Cell Rep..

[115] Bruzelius A., Kidnapillai S., Drouin-Ouellet J., Stoker T., Barker R.A., Rylander Ottosson D. (2021). Reprogramming Human Adult Fibroblasts into GABAergic Interneurons. Cells.

[116] Colasante G., Lignani G., Rubio A., Medrihan L., Yekhlef L., Sessa A., Massimino L., Giannelli S.G., Sacchetti S., Caiazzo M. (2015). Rapid Conversion of Fibroblasts into Functional Forebrain GABAergic Interneurons by Direct Genetic Reprogramming. Cell Stem Cell.

[117] Caiazzo M., Giannelli S., Valente P., Lignani G., Carissimo A., Sessa A., Colasante G., Bartolomeo R., Massimino L., Ferroni S. (2015). Direct conversion of fibroblasts into functional astrocytes by defined transcription factors. Stem Cell Rep..

[118] Mertens J., Paquola A.C.M., Ku M., Hatch E., Bohnke L., Ladjevardi S., McGrath S., Campbell B., Lee H., Herdy J.R. (2015). Directly Reprogrammed Human Neurons Retain Aging-Associated Transcriptomic Signatures and Reveal Age-Related Nucleocytoplasmic Defects. Cell Stem Cell.

[119] Mollinari C., Zhao J., Lupacchini L., Garaci E., Merlo D., Pei G. (2018). Transdifferentiation: a new promise for neurodegenerative diseases. Cell Death Dis..

[120] Oh Y.M., Lee S.W., Kim W.K., Chen S., Church V.A., Cates K., Li T., Zhang B., Dolle R.E., Dahiya S. (2022). Age-related Huntington's disease progression modeled in directly reprogrammed patient-derived striatal neurons highlights impaired autophagy. Nat. Neurosci..

[121] Victor M.B., Richner M., Olsen H.E., Lee S.W., Monteys A.M., Ma C., Huh C.J., Zhang B., Davidson B.L., Yang X.W., Yoo A.S. (2018). Striatal neurons directly converted from Huntington's disease patient fibroblasts recapitulate age-associated disease phenotypes. Nat. Neurosci..

