# Supplementary material for: RNA variant assessment using transactivation and transdifferentiation
Source: Am J Hum Genet. 2024 Jul 30;111(8):1673–99. doi: 10.1016/j.ajhg.2024.06.018 (PMC11339655; doi:10.1016/j.ajhg.2024.06.018)
Supplement: Document S1. Figures S1–S26, PERSYST Investigator Team affiliations, and acknowledgments [file mmc1.pdf]

The American Journal of Human Genetics, Volume *111*

Supplemental information

RNA variant assessment

using transactivation and transdifferentiation

Emmylou C. Nicolas-Martinez, Olivia Robinson, Christian Pflueger, Alison Gardner, Mark A. Corbett, Tarin Ritchie, Thessa Kroes, Clare L. van Eyk, Ingrid E. Scheffer, Michael S. Hildebrand, Jean-Vianney Barnier, Véronique Rousseau, David Genevieve, Virginie Haushalter, Amélie Piton, Anne-Sophie Denommé-Pichon, Ange-Line Bruel, Sophie Nambot, Bertrand Isidor, John Grigg, Tina Gonzalez, Sondhya Ghedia, Rhett G. Marchant, Adam Bournazos, Wui-Kwan Wong, Richard I. Webster, Frances J. Evesson, Kristi J. Jones, PERSYST Investigator Team, Sandra T. Cooper, Ryan Lister, Jozef Gecz, and Lachlan A. Jolly

Figure S1

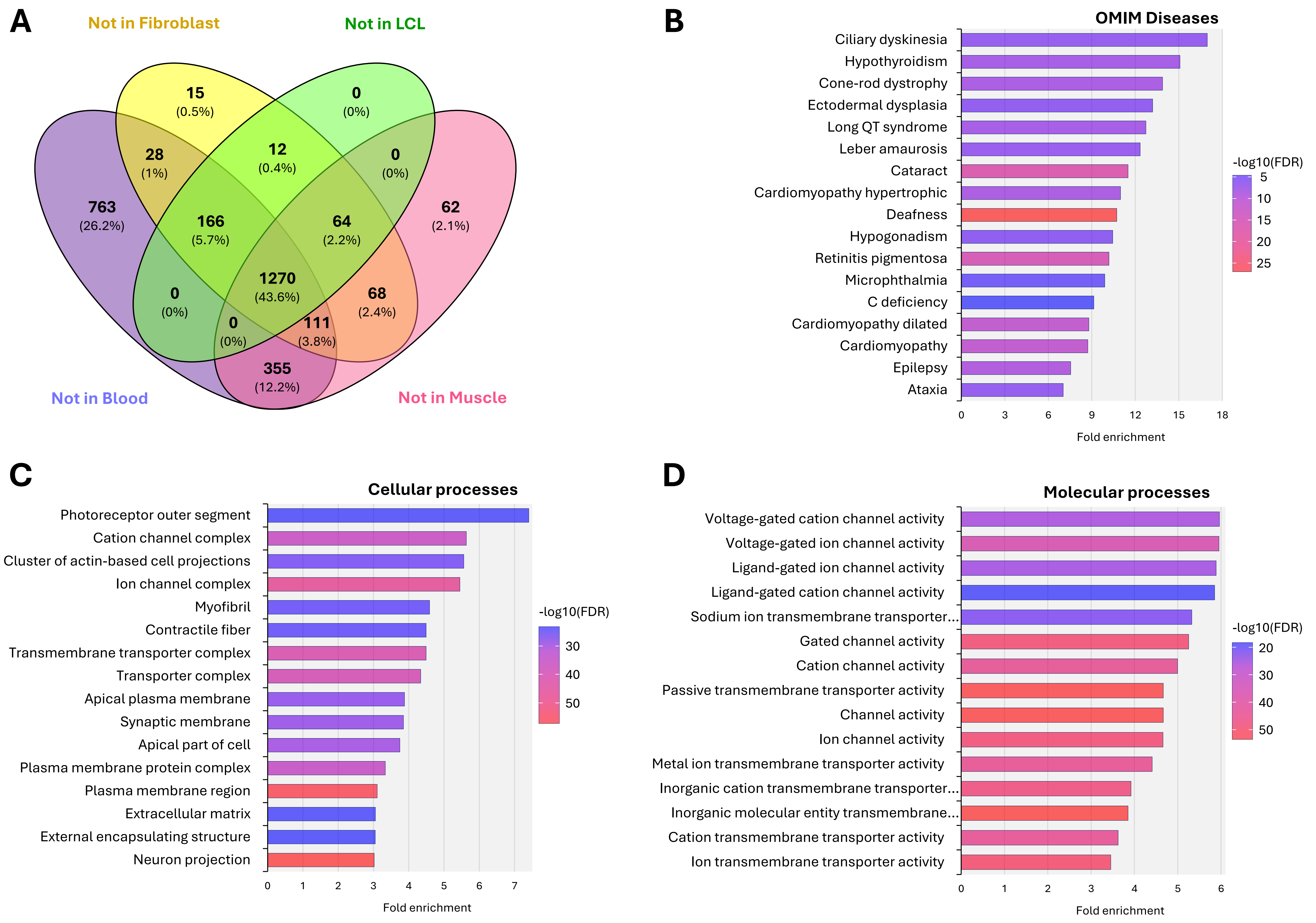

Figure S1. Characteristics of Silent Mendelian Genes (SMGs). (A) Analysis of 4878 Mendelian Disease Genes (Nijmegen DG Panel 3.2.0) using Minimum Required Sequencing Depth (MRSD) algorithm identified 1436 gene which are not sufficiently expressed in the most frequently clinically accessible tissues of whole blood, blood-derived lymphoblastoid cell lines, or human dermal fibroblasts for the purpose of conducting robust analysis of mRNA splicing using short read RNAseq. These genes are termed silent mendelian genes (SMGs). If a muscle biopsy were available, then this could supply sufficient RNA for analysis of a 166 of the SMGs. **(B-D)** Top-ranked Gene Ontology Processes Enriched in the 1436 SMGs. Top ranking OMIM diseases (B), Cellular Processes (C), and molecular processes (D) of the 1436 SMGs at p-value <0.05. Gene Ontology performed using ShinyGO 0.77 and ranked based on Fold enrichment and (FDR). pValues are FDR-corrected.

Figure S2

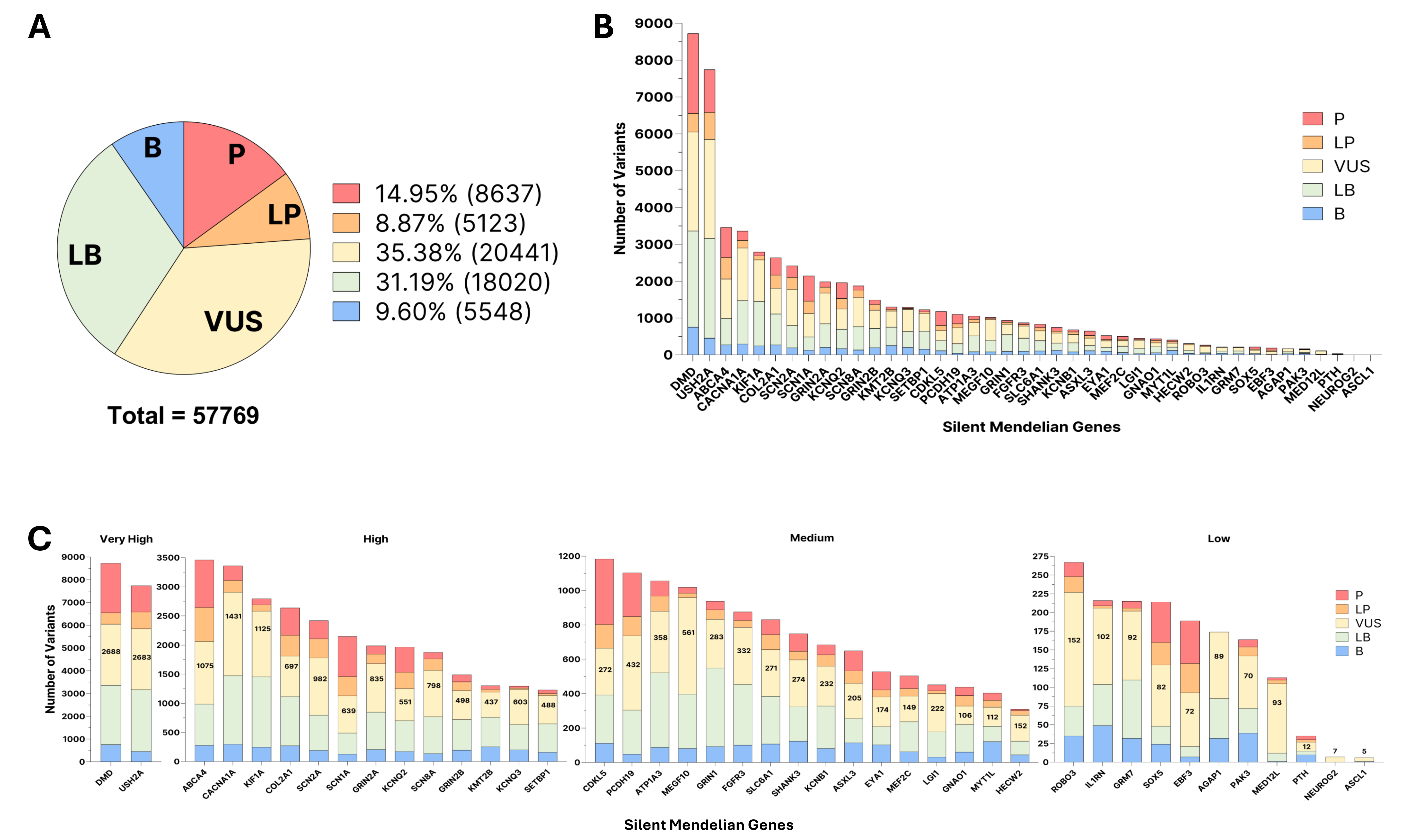

Figure S2. ClinVar variants associated with 40 SMGs targeted in transactivation screen. Numbers of total variants is given, in addition to the proportion classified as pathogenic (P), likely pathogenic (LP).

Figure S3

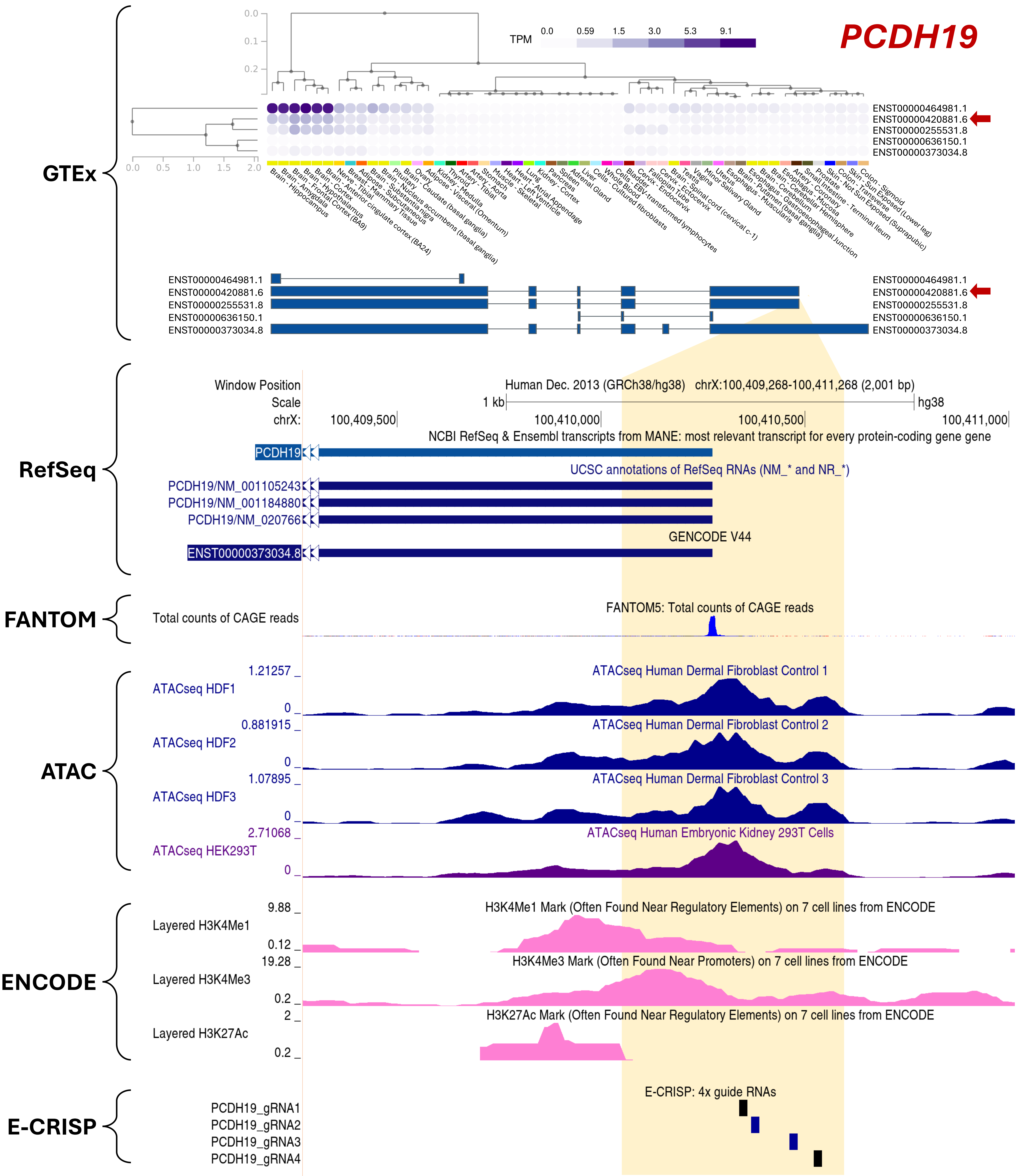

Figure S3. gRNA designs informed through multi-omic assessment. Aggregation of information on isoform expression, transcriptional start site, chromatin accessibility and epigenetic information informs gRNA design. Figure shows and example of multi-omic information utilised in the design of gRNAs for PCDH19. GTEx is first queried to identify isoforms of relevance in the clinically relevant tissue. This identifies relevant 5' ends of transcripts to target, indicted (on this occasion) by the red arrows. Reference transcripts are then located in UCSC browser. The transcriptional start site (TSS) of target gene isoform is extracted from FANTOM5 CAGE data and uploaded as a custom track. This defines the promoter = 500bp region upstream of the TSS (yellow shade). Open chromatin regions (considered most suitable for gRNA placement) identified by ATAC-seq analysis (see Material and Methods) of three human dermal fibroblast lines (HDF1-3) and HEK293T cells are also uploaded as a custom track. Epigenetic feature tracks are also visualised to provide additional information on the promoter state. We utilise chromatin immunoprecipitation sequencing (ChIP-Seq) data derived from the ENCODE project derived from Normal Human Lung Fibroblasts (NHLF) to identify different histone modifications around the promoter of target gene including H3K4Me1 (enriched at active and primed enhancers, and an essential feature of poised chromatin), H3K4Me3 (associated with transcriptionally active or poised chromatin) and H3K27Ac (highly enriched in active enhancers and promoters). Finally, the genomic sequence of the promoter is submitted to the gRNA design tool E-CRISP which returns a series of gRNAs ranked by specificity and uniqueness. These are uploaded to UCSC and four high ranking, non-overlapping gRNAs are selected with preference (where possible) given to those falling in open and active/poised chromatin regions. Uniqueness of the gRNA sequences is validated by UCSC BLAT tool.

Figure S4

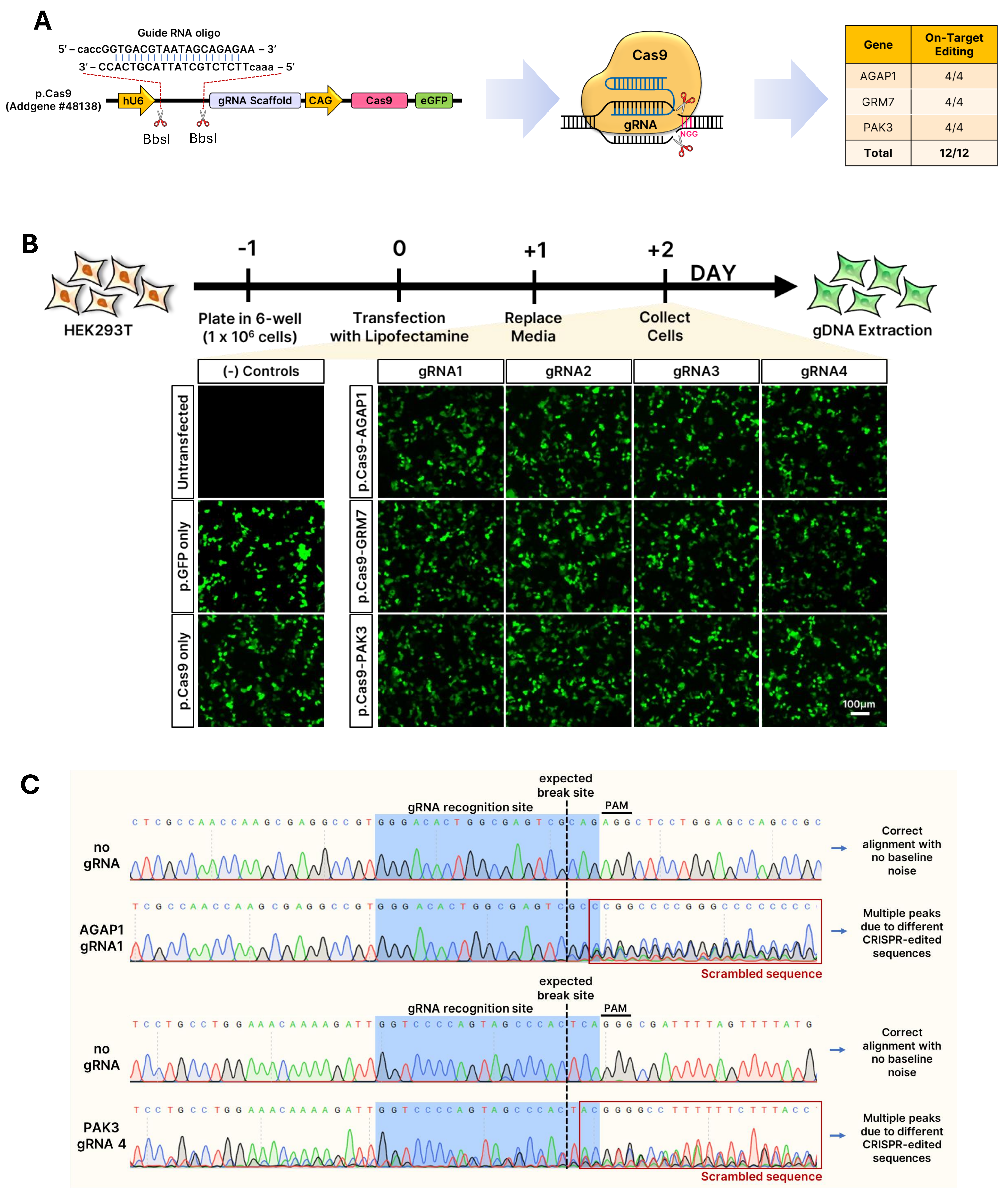

Figure S4. gRNAs direct Cas9 to desired genomic regions. A set of 12 designed gRNAs were examined for their ability to recruit Cas9 to the target genomic loci using the Cas9 nuclease assay. **(A)** Overview and summary of the Cas9 nuclease assay. Each of the 12 individual guide RNAs were cloned into the p.Cas9 plasmid. Diagram shows p.Cas9 plasmid (Addgene #48138) which encodes the active Cas9 enzyme fused to a green fluorescent protein (GFP). The gRNA oligonucleotide pairs synthesised with the appropriate 5' and 3' overhangs were inserted into the p.Cas9 plasmid via the BbsI cloning sites, in between the Pol III promoter hU6 and the gRNA scaffold (aka tracrRNA). Expression of each of these plasmids in cells direct Cas9 nuclease to the gRNA's target genomic loci and introduce double stranded DNA breaks which undergo error-prone repair through the non-homologous end-joining pathway. The introduction of errors into the region illustrates successful targeting of the Cas9 nuclease by its gRNA. In summary, of the 12 gRNAs tested, 100% of them successfully directed Cas9 to its target loci. **(B)** The experimental pipeline shows the transfection of HEK293T cells with p.Cas9-gRNA using Lipofectamine 2000, with the cells collected at Day 2 post-transfection for genomic DNA extraction. The representative fluorescent images show the expression of green fluorescent proteins (eGFP) indicating successful delivery and transient expression of the transgene. Scale bar = 100μm. **(C)** Representative Sanger Sequence chromatograms of PCR amplicons spanning the gRNA targeted genomic DNA regions (gDNA). Mutations in these sequences reveal successful delivery of the Cas9 nuclease. Mutations were found in each case, and include scrambled sequences (as shown), indels, and base substitution. Blue background indicates gRNA target sequence, with the expected break site indicated. PAM (protospacer adjacent motif) denotes the NGG sequence.

Figure S5

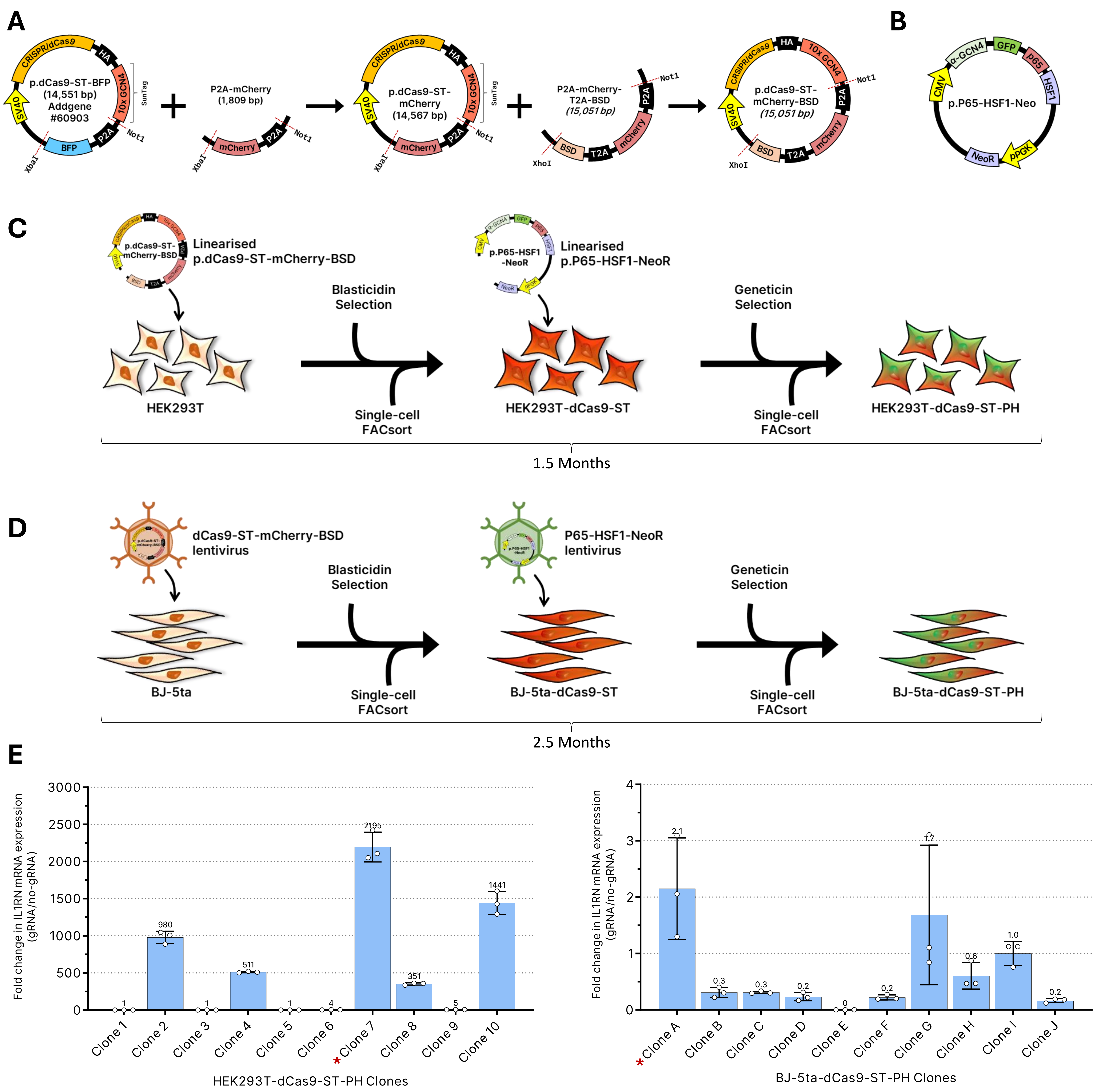

Figure S5. Generating HEK293T and BJ-5ta clonal cell lines stably expressing dCas9-ST-PH for gRNA transactivation screen. (A) Modification of the p.dCas9-ST vector to permit selection of cells expressing the transgene by FACS, microscopy, and antibiotic selection. The plasmid p.dCas9-ST-BFP was procured from Addgene (#60903). To replace the blue fluorescent protein (BFP) tag with a red fluorescent protein (mCherry), the P2A-mCherry fragment was inserted via the cloning sites NotI and XbaI, generating the transgene p.dCas9-ST-mCherry (*Note: This version was specifically used for lentiviral delivery into HDFs*). To allow Blasticidin selection, the fragment is improved into P2A-mCherry-T2A-BSD and was inserted via the cloning sites, NotI and XhoI, generating the final transgene p.dCas9-ST-mCherry-BSD (*Note: This transgene is the specific version used to generate stable HEK293T and BJ-5ta clonal cells*). All fragment inserts were synthesised by GenScript and supplied within the pUC57 plasmid backbone. **(B)** The p.P65-HSF1-NeoR encodes eGFP and neomycin markers, allowing FACs and antibiotic selection. The illustration shows the vector map of p.P65-HSF1-NeoR (gift from Prof. Ryan Lister) which is used to generate stable HEK293T and BJ-5ta clonal cells. The plasmid contains the transactivating domains (TADs) p65 and HSF1 (heat shock factor 1) fused to a single-chain variable fragment (scFv) that recognises the GCN4 epitope, as well as enhanced green fluorescent protein (eGFP) and Neomycin resistance gene, utilised as selection markers. **(C and D)** Generating HEK293T and BJ-5ta clonal cell lines stably expressing dCas9-ST-PH for gRNA transactivation screen. Diagrams show the experimental pipeline for generating and selecting HEK293T and BJ-5ta clonal cells. Briefly, the dCas9-ST-mCherry-BSD was first delivered to the cells, followed by Blasticidin selection. After selection and expansion, cells were then sorted via fluorescence-activated cell sorting (FACs) to create single cell clones. The resulting dCas9-ST-stable cells are then further modified with the P65-HSF1-NeoR transgene. The cells are then subjected to Geneticin selection and lastly, went through single-cell FACs to select clonal cells with varying fluorescence intensity. Note: For HEK293T, transgenes were first linearised and transfected using standard protocol for Lipofectamine 3000. For BJ-5ta, transgenes were packaged in lentivirus and delivered at MOI 20 (dCas9-ST-mCherry-BSD) and MOI 30 (P65-HSF1-Neo). **(E)** Screening of dCas9-ST-PH clonal stable cells. To screen for the most potent transactivating dCas9-ST-PH clonal cell lines, a single guide (IL1RN-gRNA4) was delivered to cells (via lipofection for HEK293T and lentiviral delivery for BJ-5ta). mRNA was isolated an IL1RN expression assessed by RT-qPCR. The bar graphs show the activation levels of *IL1RN* in the 10 different clonal cell lines. Data presented are relative gene expression generated from RT-qPCR with values normalised to *ACTB*. RT-qPCR data are presented as mean and standard deviation from three technical replicates further normalised to negative control (no-gRNA). Clone 7 and Clone A from HEK293T and BJ-5ta were selected, respectively, for use in gRNA screening experiments (red asterisk).

Figure S6

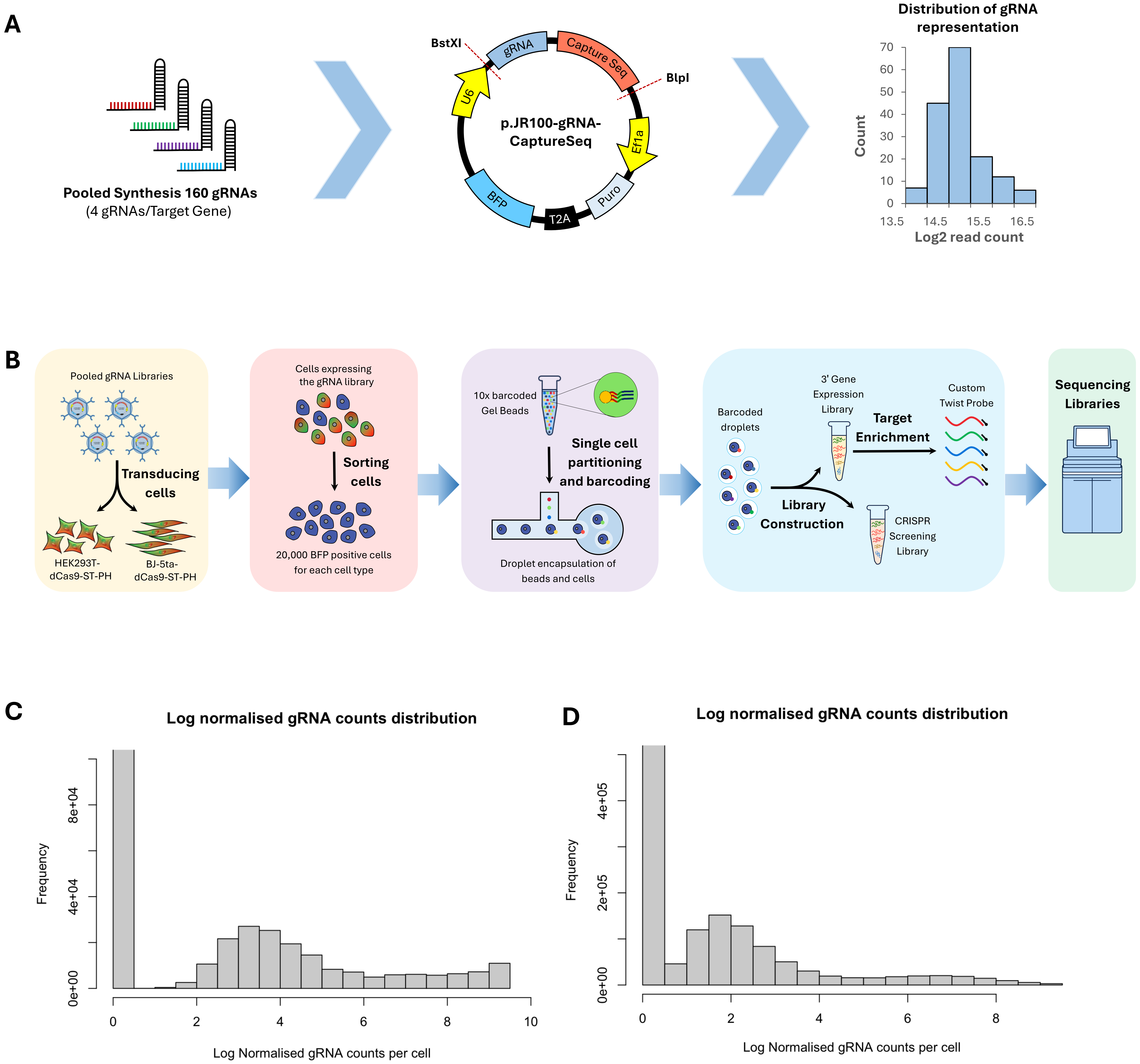

Figure S6. Creation of the gRNA pooled vector library. (A) A total of 160 individual gRNA sequences targeting 40 SMGs (4 gRNAs per SMG) were synthesized and cloned into the p.JR100-BFP vector (Addgene #187240) by VectorBuilder via the BstXI and BlpI cloning sites. The p.JR100-BFP vector encodes for the mouse U6 Pol III, the unique barcode to capture the gRNA, EF1α driving the expression of puromycin-BFP marker expression. The vector library was sequenced using 150bp paired end reads on an Illumina Novaseq platform. Reads were aligned to the gRNA reference with 100% representation of the gRNAs observed as a normal distribution (>93% of all reads mapping correctly (>4.8 Million Reads). **(B)** Outline of the Perturb-Seq work-flow. The gRNA vector library was packaged into lentiviral particles and delivered to cells at low MOI to facilitate expressing just a single gRNA species in each cell. Transduced cells are selected with puromycin for 2 days and then FACs purified (using BFP) prior to preparation for the 10X Genomics Single Cell Sequencing Workflow. Pooled sequencing libraries were sequenced on an Illumina Novaseq. **(C)** For HEK293T$^{dCas9\text{-}ST\text{-}PH}$ cells, 21292 cells passed quality control for assessment. The distribution of the number of gRNA molecules expressed in each cell is shown. D. For HDF$^{dCas9\text{-}ST\text{-}PH}$ cells, 20305 cells passed quality control for assessment. The distribution of the number of gRNA molecules expressed in each cell is shown.

Figure S7

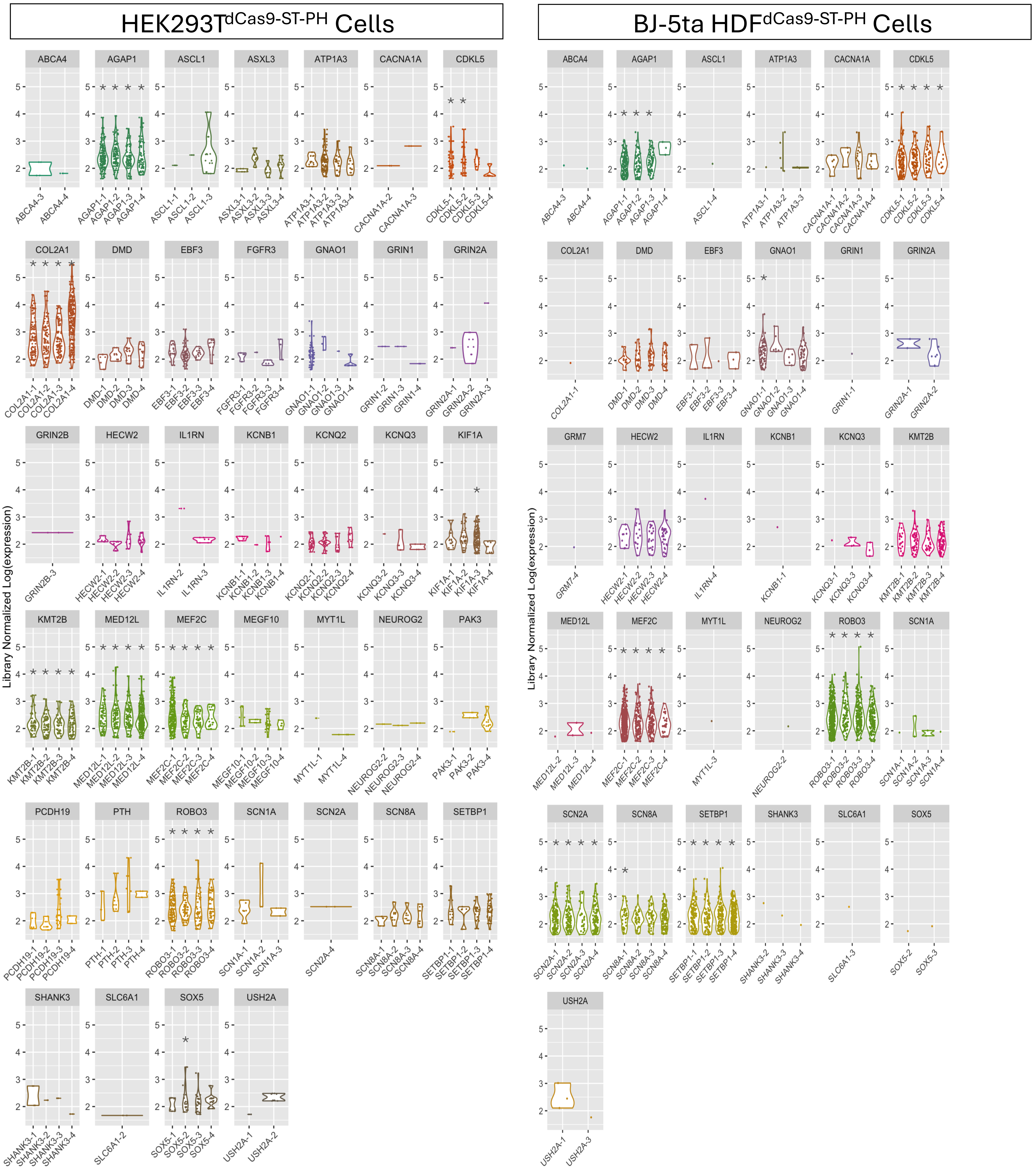

Figure S7. Transactivation screen using single cell transcriptomics. A pooled gRNA expression plasmid library (160 gRNAs; 4 gRNAs per gene, targeting 40 SMGs) was delivered by lentivirus to the HEK293T$^{dCas9\text{-}ST\text{-}PH}$ and HDF$^{dCas9\text{-}ST\text{-}PH}$ cells using a low multiplicity of infection to deliver ~1 gRNA vector per cell. >20,000 cells per cell line were subjected to single cell Perturb-seq using the 10X genomic platform. Cells expressing >6 of a given gRNAs species were analysed for expression of their target gene. *p-adjusted <0.05

Figure S8

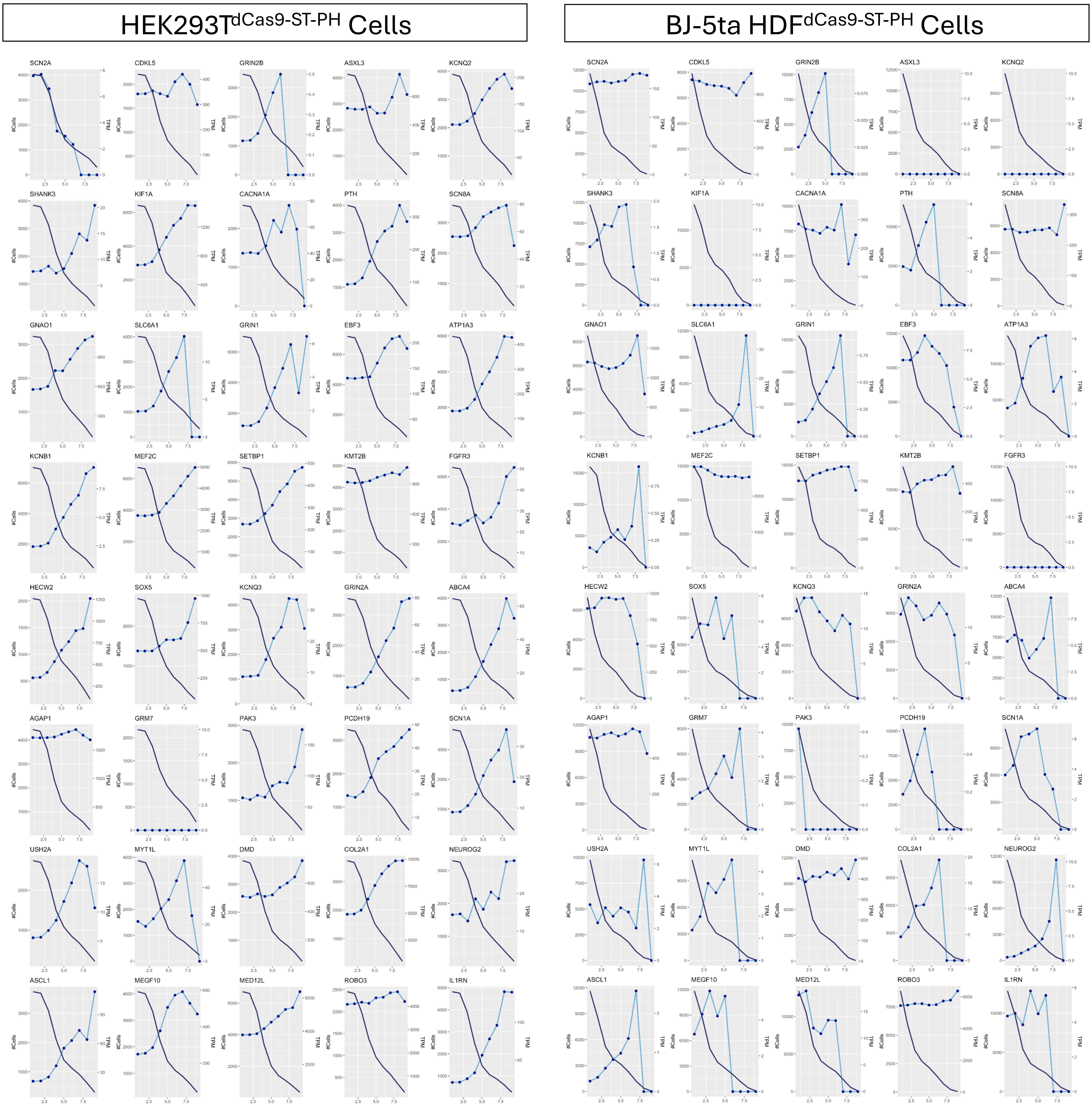

Figure S8. Relationships between gRNA expression, number of cells analysed and target gene expression in scRNAseq gene transactivation screen. A pooled gRNA expression plasmid library (160 gRNAs; 4 gRNAs per gene, targeting 40 SMGs) was delivered by lentivirus to the HEK293T$^{dCas9-ST-PH}$ and HDF$^{dCas9-ST-PH}$ cells using a low multiplicity of infection to deliver ~1 gRNA vector per cell. >20,000 cells per cell line were subjected to single cell Perturb-seq using the 10X genomic platform. For many genes, the number of gRNAs per cell is positively associated with target gene expression, and negatively associated with cell number analysed. Data is pooled from all 4 gRNAs per gene. Dark blues lines are number of cells, light blue lines are transcripts per million (TPM), x-axis is gRNA expression.

Figure S9

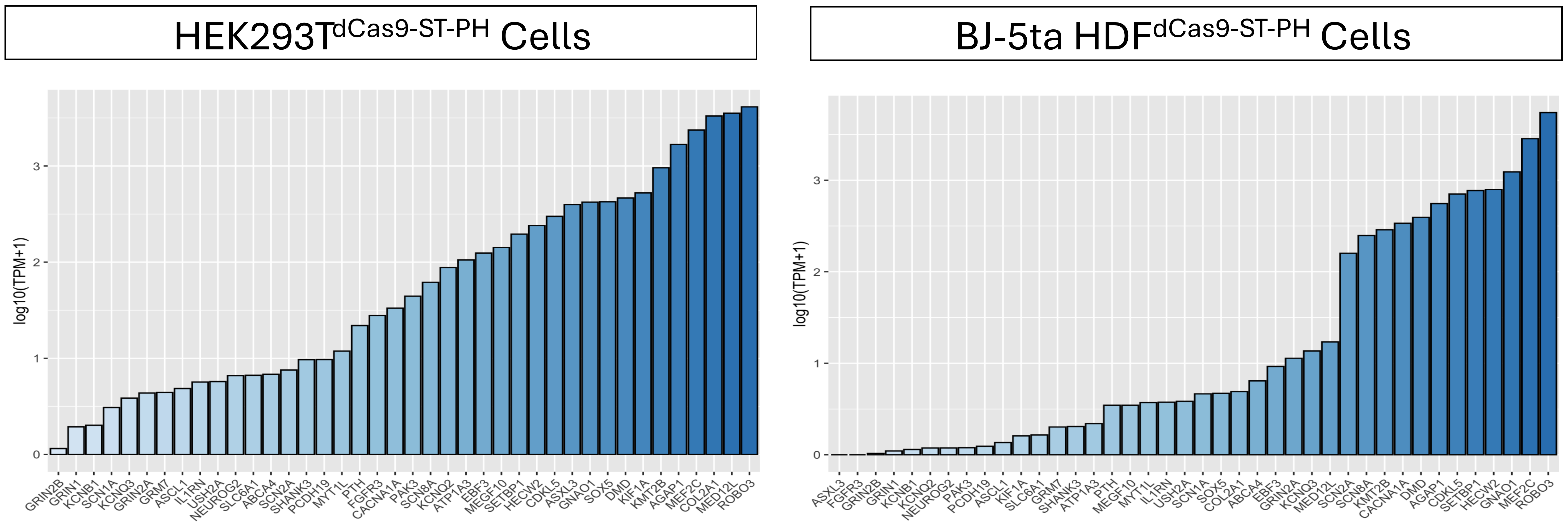

Figure S9. Pseudo-bulk cell analysis of single cell transcriptomics data generated from the scRNAseq Gene Transactivation Screen. A pooled gRNA expression plasmid library (160 gRNAs; 4 gRNAs per gene, targeting 40 SMGs) was delivered by lentivirus to the HEK293T$^{dCas9\text{-}ST\text{-}PH}$ and HDF$^{dCas9\text{-}ST\text{-}PH}$ cells using a low multiplicity of infection to deliver ~1 gRNA vector per cell. >20,000 cells per cell line were subjected to single cell Perturb-seq using the 10X genomic platform. Single cell data was collapsed into a pseudo-bulk cell analyses in which the expression of genes is expressed as the number of reads mapping to a gene as a proportion of the entire number of reads generated in the experiment. (Transcripts per million; TPM).

Figure S10

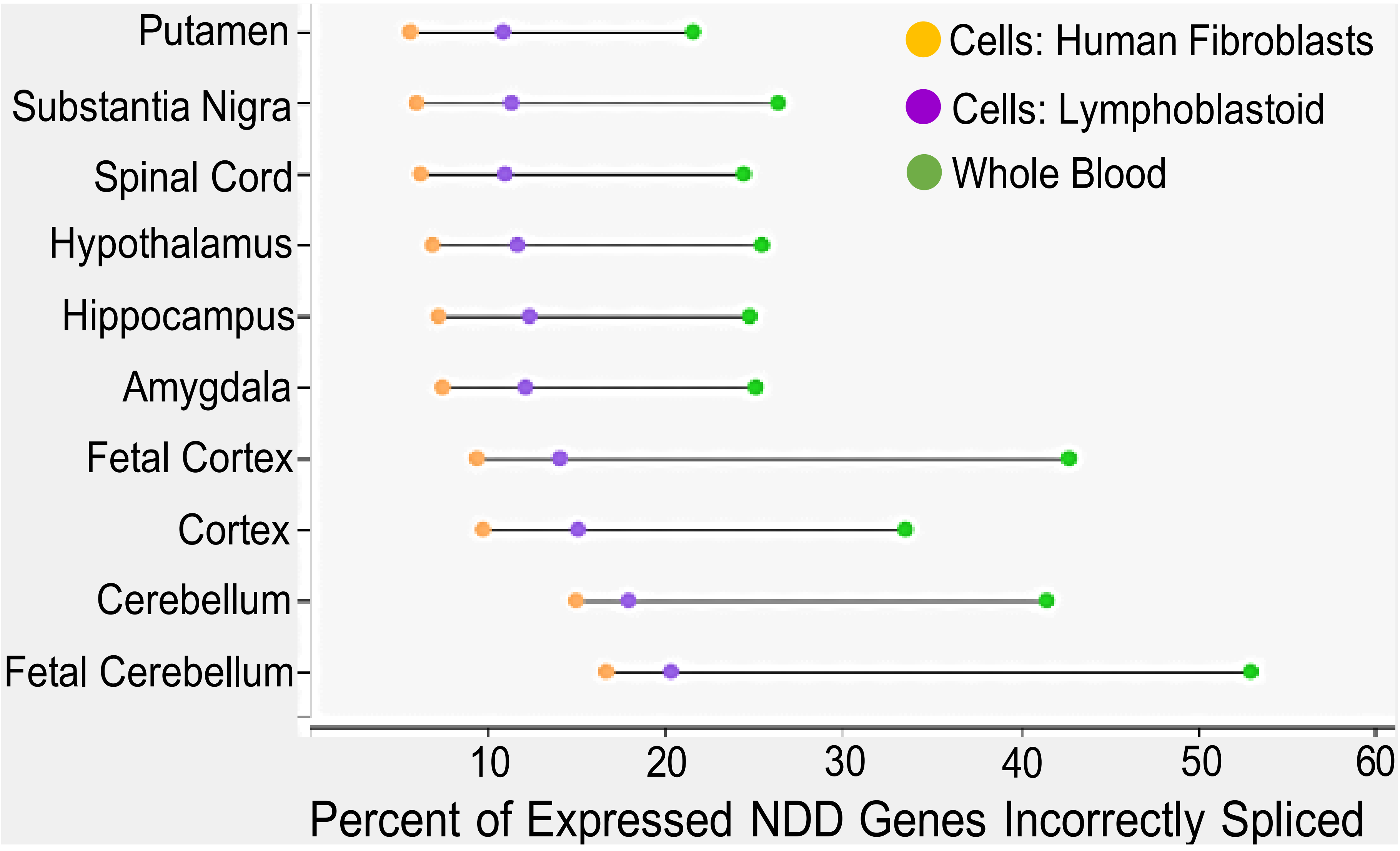

Figure S10. Neurological disorder genes are faithfully spliced in Human Dermal Fibroblasts. Comparison of local splicing events in 2484 broadly expressed neurological disorder genes between clinically relevant tissues (different brain regions from GTEx) and clinically accessible tissues including human dermal fibroblasts, lymphoblastoid cells lines, and whole blood. Analysis conducted using the MAJIQ-CAT tool which reports on the percentage of genes which are not correctly spliced in the clinically accessible tissue using the clinically relevant tissue as a reference. In all comparisons, human dermal fibroblasts displayed the least percentage of incorrectly spliced genes (6-16%), superior to LCLs and blood in which genes were more frequently incorrectly spliced.

Supplemental Figure S11

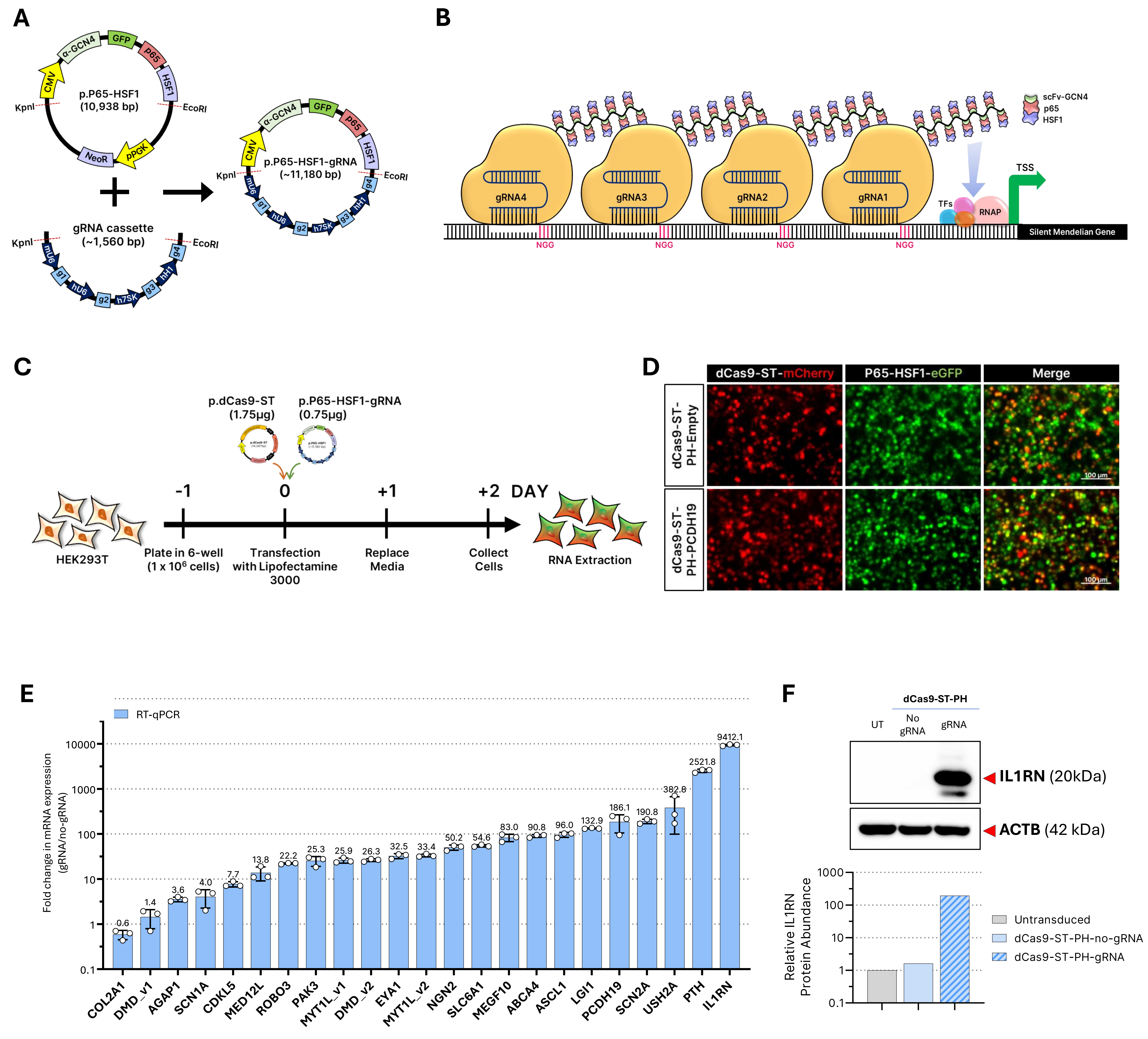

Supplemental Figure S11. dCas9-ST-PH-4gRNA based transactivation of SMGs in HEK293Ts. A. Engineering of p.P65-HSF1 and 4 gRNA expression cassettes into a single vector. Schematic diagram of p.P65-HSF1 and its cloning sites, KpnI and EcoRI used for the insertion of a cassette containing 4 guide RNA expression cassettes. In the multiplex gRNA expression cassette, each guide was inserted downstream of a different Pol III promoter including the mouse mU6, and human hU6, h7SK, and hH1 promoters. EcoRI and KpnI restriction sites flank the entire cassette to facilitate cloning. The negative control consists of an empty cassette with no gRNAs (encoding only the Pol III promoter and scaffold transactivating CRISPR-RNA aka. tracrRNA). All gRNA expression cassettes were synthesised by GenScript and supplied within the pUC57 plasmid backbone **B. A multiplex of four dCas9 proteins can each recruit up to ten copies of p65-HSF1 proteins to the target gene promoter on each allele.** Illustration of four guide RNAs targeting different sites upstream of a SMGs' TSS. Each gRNA recruits dCas9-ST scaffold to the binding site, and each dCas9-ST recruits up to ten copies of p65-HSF1 transactivating domains (TADs) through the scFv-GCN4 and SunTag (10 copies of GCN4 epitope) interaction. The TADs act like synthetic transcription factors to promote the assembly of endogenous transcriptional co-regulators and recruit RNA Pol II to the site, culminating in transcription of the endogenous promoter of the target SMG. **C. Transactivation of SMGs in HEK293T cells via transient transfection.** Diagram shows the experimental pipeline for the transient expression of dCas9-ST-PH-gRNA in HEK293T using Lipofectamine 3000 in a 6-well format. Briefly, the dCas9-ST (1.75µg) and P65-HSF1-gRNA (0.75µg) are co-transfected. One day post-transfection, cells had a fresh media change, and two days post transfection, cells were collected for RNA analysis. **D. dCas9-ST-PH-gRNA complex is efficiently co-expressed in HEK293T.** Representative images showing the co-expression of green and red fluorescent proteins (eGFP and mCherry) two days after co-transfection of p.dCas9-ST and p.P65-HSF1-gRNA, indicating successful delivery and expression of the transgenes. Scale bar = 100µm. **E. dCas9-ST-PH-4gRNA transactivation experiments in HEK293T cells revealed that all SMG tested can be transactivated at varying levels.** Bar graph showing the activation of 21 SMGs in HEK293T via transient expression of dCas9-ST-PH-gRNA. Note that for two genes, *DMD* and *MYT1L*, two unique sets of guides were assayed (v1 and v2). Data presented are relative gene expression generated from RT-qPCR with values normalised to *ACTB*. RT-qPCR data are presented as mean and standard deviation from three technical replicates normalised to negative control (dCas9-ST-PH-no-gRNA). **F. Transactivation of mRNA expression results in protein production.** Western blot shows IL1RN protein in dCas9-ST-PH-gRNA treated HEK293T cells but not in the negative controls, untransduced (no treatment) and no-gRNA (cells treated with dCas9-ST-PH-no-gRNA). IL1RN (mouse-anti-IL1RN, ThermoFisher, #TA803422S), and β-actin (mouse-anti-- β-Actin, Sigma-Aldrich, #A2228) proteins at expected molecular weights. Western data is quantified in the graph.

Figure S12

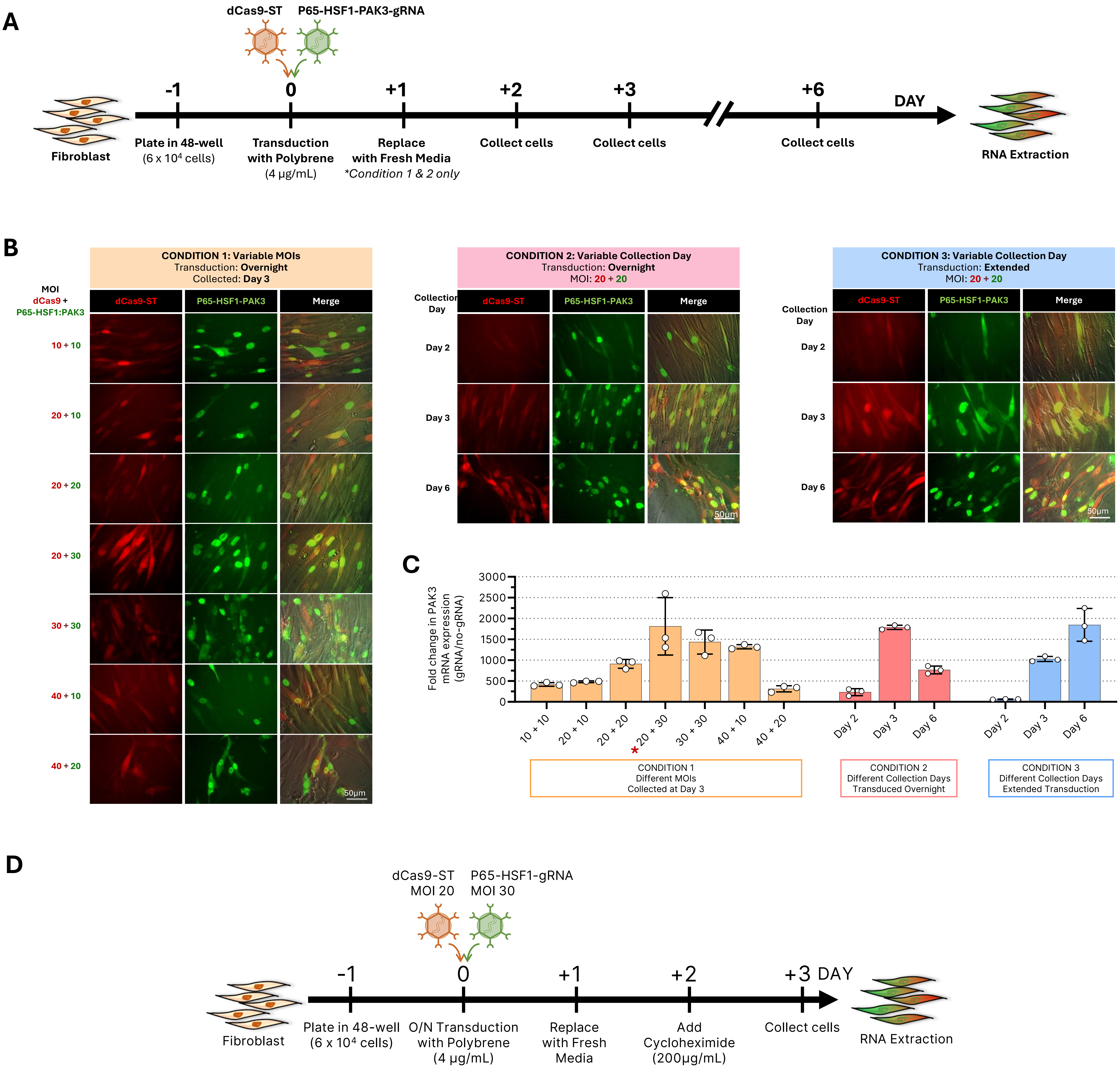

Figure S12. Optimisation of Lentiviral Delivery of dCas9-ST-PH to Human Dermal Fibroblasts. (A) Experimental pipeline of lentiviral delivery of dCas9-ST-PH targeting *PAK3 to* human dermal fibroblasts (HDFs). To identify the optimum condition for the most potent transactivation, different durations of lentiviral delivery, at different multiplicity of infection (MOI) were tested using transgenes targeting *PAK3*. For *Condition 1*: Cells were transduced overnight at varying MOI combinations. 24hr after transduction, cells had a fresh media change and were collected on Day 3 post-transduction. For *Condition 2*: Cells were transduced overnight with transgenes at equal MOI of 20. 24hr after transduction, cells had a fresh media change and were collected at either Day 2, 3, or 6 post-transductions. For *Condition 3*: Cells were transduced with transgenes at equal MOI of 20. The transduction media containing the virus has not been removed, instead was topped up with fresh media. Cells were also collected at either Day 2, 3, or 6, post-transduction**. (B)** dCas9-ST-PH-PAK3 transgene co-expression. Representative live-cell fluorescent images of cells expressing transgenes dCas9-ST (mCherry) and P65-HSF1:PAK3 (eGFP) were used to assess level of transduction efficiency at different conditions. **(C)** PAK3 mRNA expression serves as a functional readouts to identify the most potent condition. Bar graph showing the varying level of transactivation of *PAK3* mediated by dCas9-ST-PH-PAK3 under different conditions. Data presented are relative gene expression generated from RT-qPCR with values normalised to *ACTB*. RT-qPCR data are presented as mean and standard deviation from three technical replicates normalised to negative control (dCas9-ST-PH-no-gRNA). Note that Condition 1 at MOI combination of 20 + 30 (dCas9-ST + P65-HSF1-PAK3-gRNA, respectively) collected on Day 3 achieved the highest level of *PAK3* RNA expression and hence was adapted for subsequent experiments with HDFs. D. Schematic of the optimised transient lentiviral transduction protocol for transactivation of genes in HDFs.

Figure S13

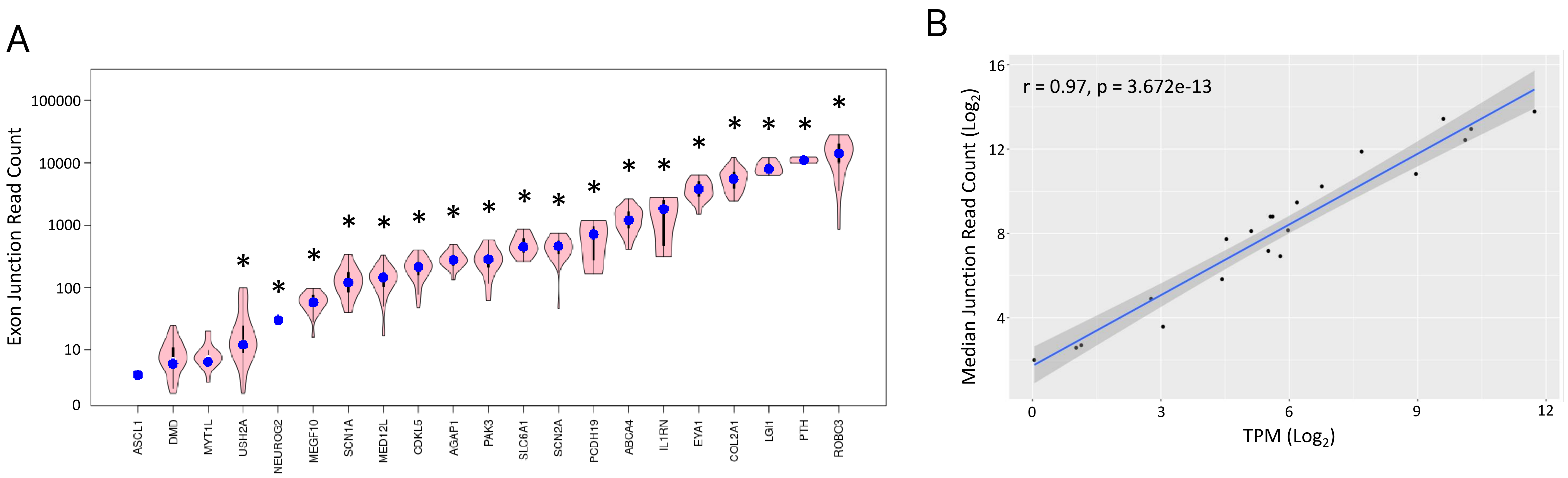

Figure S13. Number and distribution of RNA-seq reads mapping to exon-exon junctions in transactivated SMGs and their correlation to TPM. **(A)** For each transactivated SMG, the number of reads mapping across each exon-exon junction of the most predominately expressed isoform was calculated. Data is expressed as violin plot to show transcript wide distribution of read depth across junctions, with the median value highlighted in blue. Asterisks identifies the 17/20 genes with read counts satisfying the MRSD parameters used to define suitability for RNA-seq based assessment of splicing (i.e. genes with minimum 8 junction reads across 75% of junctions). **(B)** The median read depth for junctions is highly correlated with overall gene expression (transcripts per million (TPM)) following gene transactivation (Pearsons Correlation displayed).

Figure S14

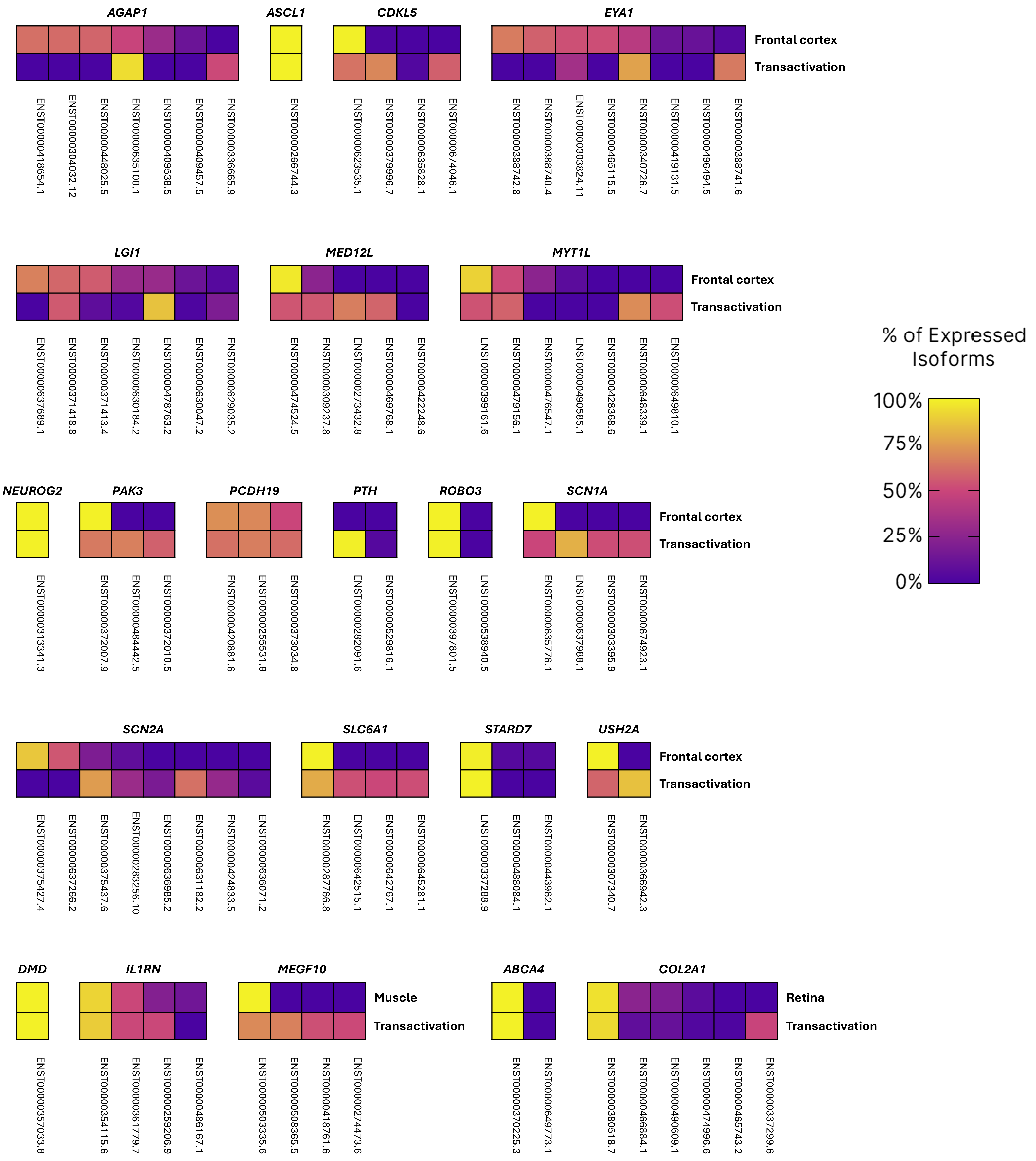

Figure S14. Transactivation of multiple isoforms of SMGs in HDFs. SMGs were transactivated in HDFs and RNA using multiplex dCas9-ST-PH-gRNA approach and subjected to short read RNAseq. Reads were mapped with Salmon and assigned to annotated gene transcripts and quantified as transcripts per million (TPM). RNA Heatmap showing the proportion of expressed gene isoforms following transactivation and compared to the clinically relevant tissue (data from GTEx).

Figure S15

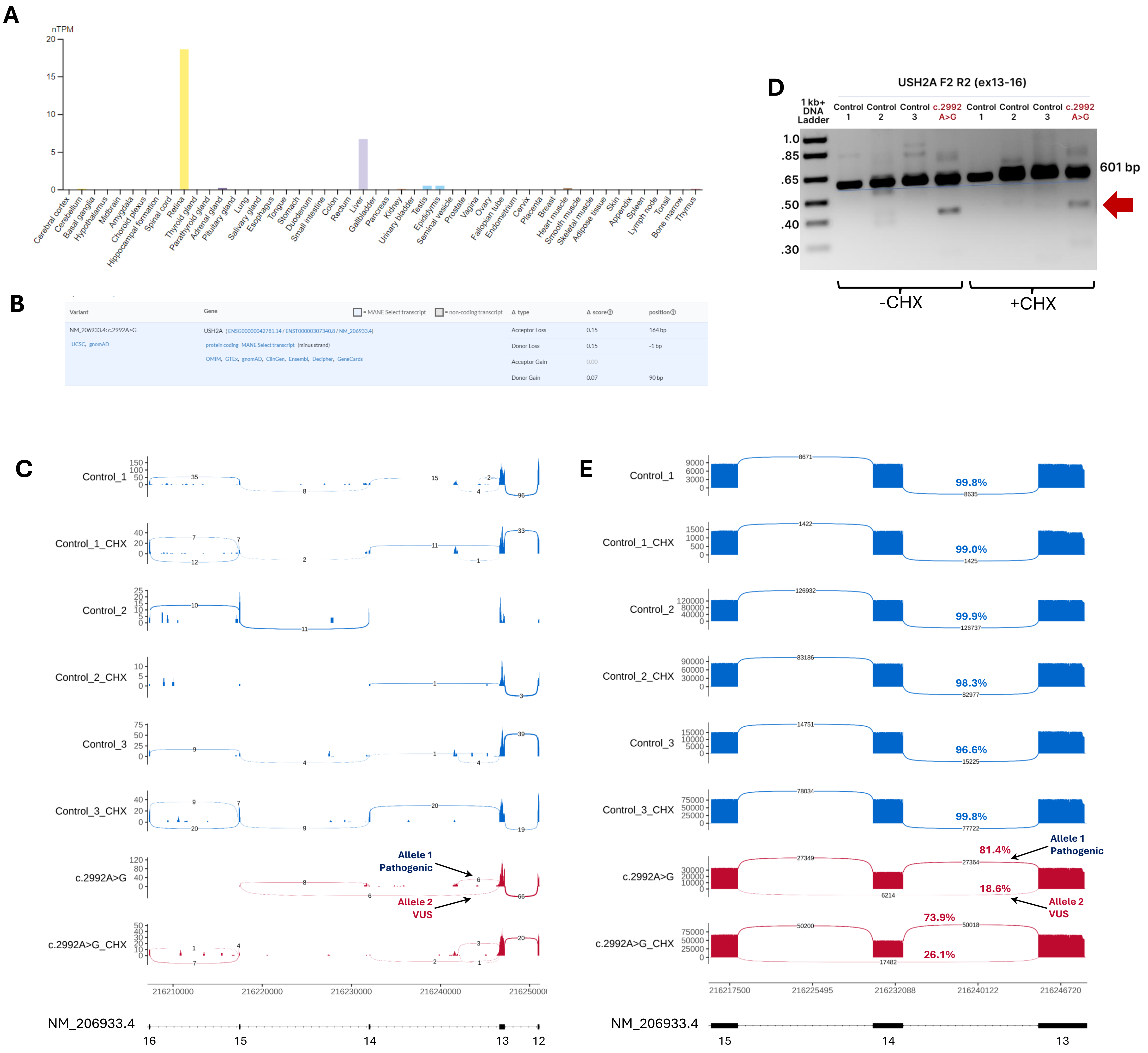

Figure S15. Transactivation of USH2A in HDFs reveals deleterious impact of a VUS on RNA splicing. (A) Bar graph of USH2A mRNA expression across human tissues (data derived from Human Protein Atlas). **(B)** Output of SpliceAI analysis of the USH2A VUS. Low delta scores indicate low probability of the variant being splice altering. **(C-E)** Investigation of the VUS impact on RNA splicing. RNA isolated following transactivation of *USH2A* in HDFs derived from healthy control (n=3) and affected (n=1) individuals in the presence and absence of CHX. **(C)** RNA subjected to short read RNAseq. Sashimi plots report *USH2A* mRNA splicing. Note that in the affected individual only, reads are found which skip exon 14. **(D)** RNA subjected to RT-PCR using primers which span exon 14. Note lower size amplicons in the VUS sample only, which align with skipping of exon 14. **(E)** RT-PCR products subjected long read Oxford Nanopore sequencing with reads presented as Sashimi plots to reflect the splicing of *USH2A* RNA template. Note skipping of Exon 14 occurs only in VUS samples. Arrows in the sashimi plots specify the reads coming from the alleles with pathogenic variant and allele with VUS as segregated by allelic phasing. Skipping of Exon 14 deletes 184 bp of the transcript (2810_2993del), and shifts the reading frame, resulting in a stop codon in Exon 15, p.(Gly937Aspfs*13). Note the number of reads from the VUS allele is slightly sensitive to CHX treatment. Sashimi plots are generated using ggsashimi. Y-axis scale on sashimi plots indicates sequence read coverage.

Figure S16

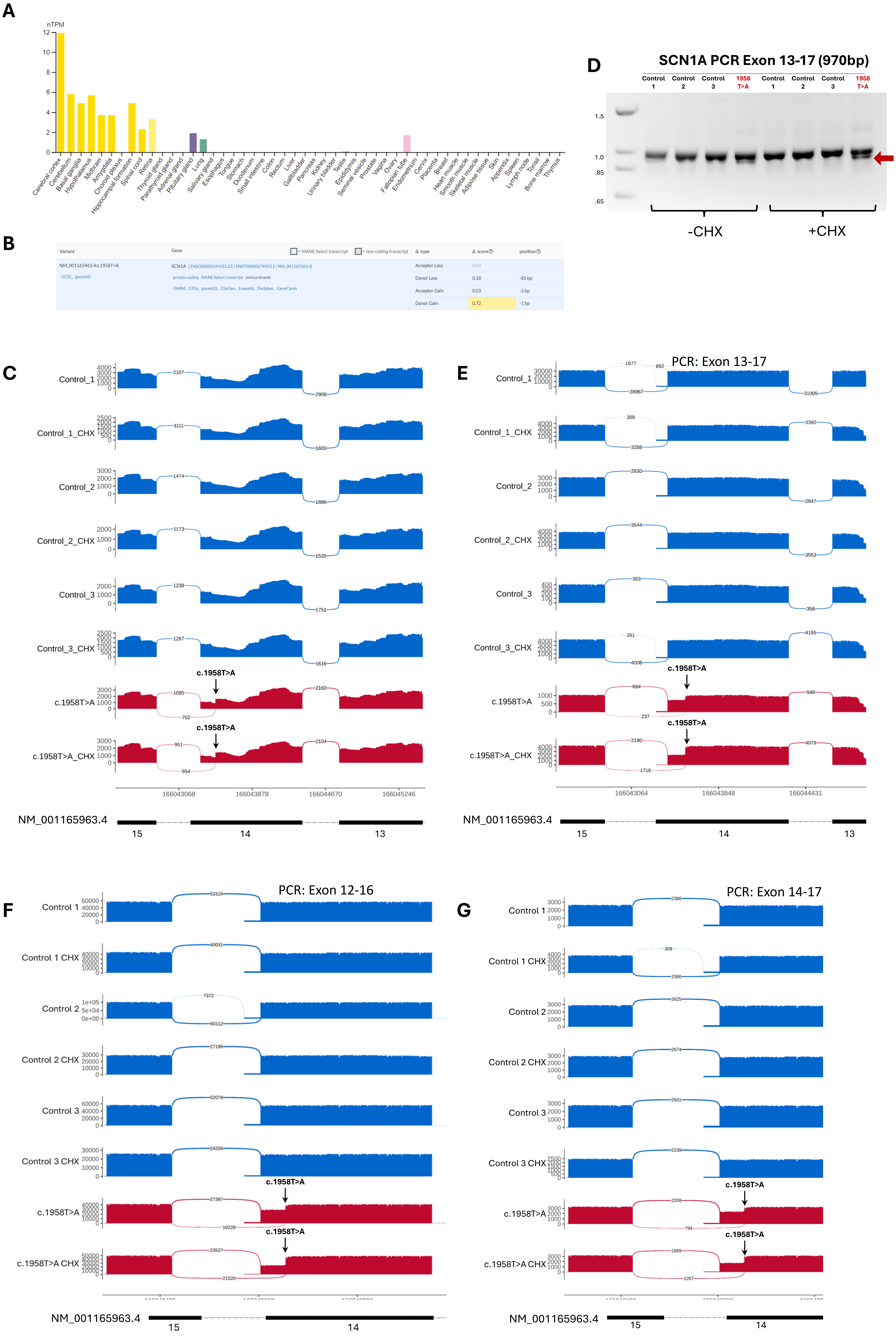

Figure S16. Transactivation of *SCN1A* in HDFs reveals deleterious impact of a VUS on RNA splicing. (A) Bar graph of *SCN1A* mRNA expression across human tissues (data derived from Human Protein Atlas). **(B)** Output of SpliceAI analysis of the SCN1A VUS. The high delta score (yellow) indicate high probability of the variant creating a new splice donor site 1 bp upstream of the variants genomic coordinate. **(C-E)** Investigation of the VUS impact on RNA splicing. RNA isolated following transactivation of *SCN1A* in HDFs derived from healthy control (n=3) and affected (n=1) individuals in the presence and absence of CHX. **(C)** RNA subjected to short read RNAseq. Sashimi plots report *SCN1A* mRNA splicing. Note that in the affected individual only, reads are found which splice out of the predicted gained splice donor site. **(D)** RNA subjected to RT-PCR using primers which span exon 14. Note lower size amplicons in the VUS sample only, which align with use of the predicted gained donor site in Exon 14. **(E-G)** Three independent RT-PCR products subjected long read Oxford Nanopore sequencing with reads presented as Sashimi plots to reflect the splicing of *SCN1A* RNA template. Note splicing out of an Exon 14 at the predicted donor gain site occurs only in VUS samples. Arrows in the sashimi plots indicate the exonic location of the VUS, which results strengthening the internal exon 13 splice donor usage. Sashimi plots are generated using ggsashimi. Y-axis scale on sashimi plots indicates sequence read coverage.

Figure S17

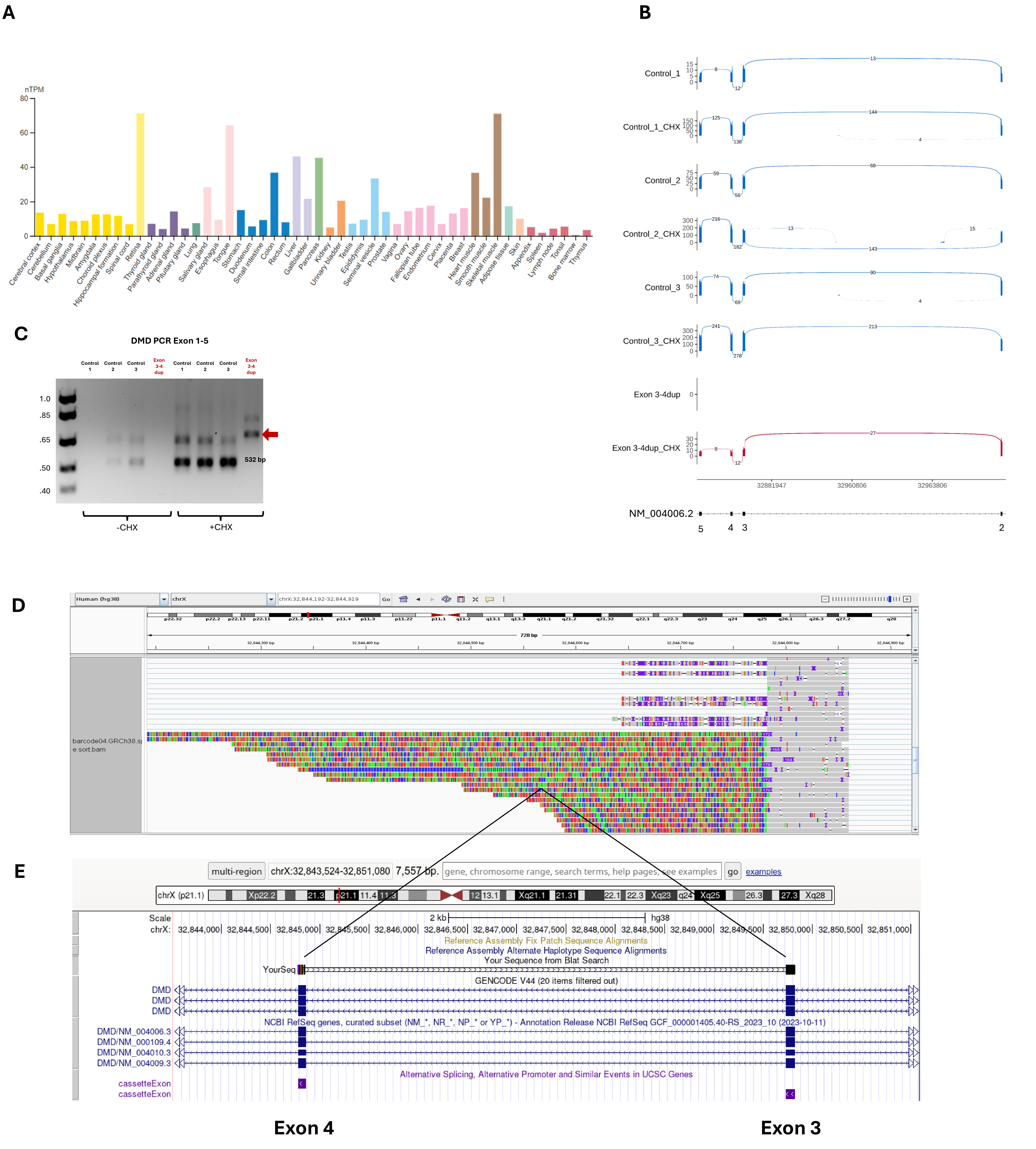

Figure S17. Transactivation of *DMD* in HDFs reveals mechanistic impact of a VUS on RNA splicing. (A) Bar graph of DMD mRNA expression across human tissues (data derived from Human Protein Atlas). **(B-E)** Investigation of the VUS impact on RNA splicing. RNA isolated following transactivation of *DMD* in HDFs derived from healthy control (n=3) and affected (n=1) individuals in the presence and absence of CHX. **(B)** RNA subjected to short read RNAseq. Sashimi plots report *DMD* mRNA splicing. Note that no reads from affected individual aligned to Exons 2-5 in the absence of CHX. **(C)** RNA subjected to RT-PCR using primers which span exons 1-5. Note higher size amplicons in the VUS sample only, which align with expected size of duplication of Exons 3 and 4. **(D)** RT-PCR products subjected long read Oxford Nanopore sequencing. IGV alignment of PCR amplicon reads to exon 4. Note misalignment of bases (coloured) into intron 4. **(E)** Misaligned sequence from D were submitted to BLAT search tool in UCSC. These sequences aligned with Exons 3 and 4 of DMD, confirming inclusion of duplicated exons 3 and 4 into transcripts.

Figure S18

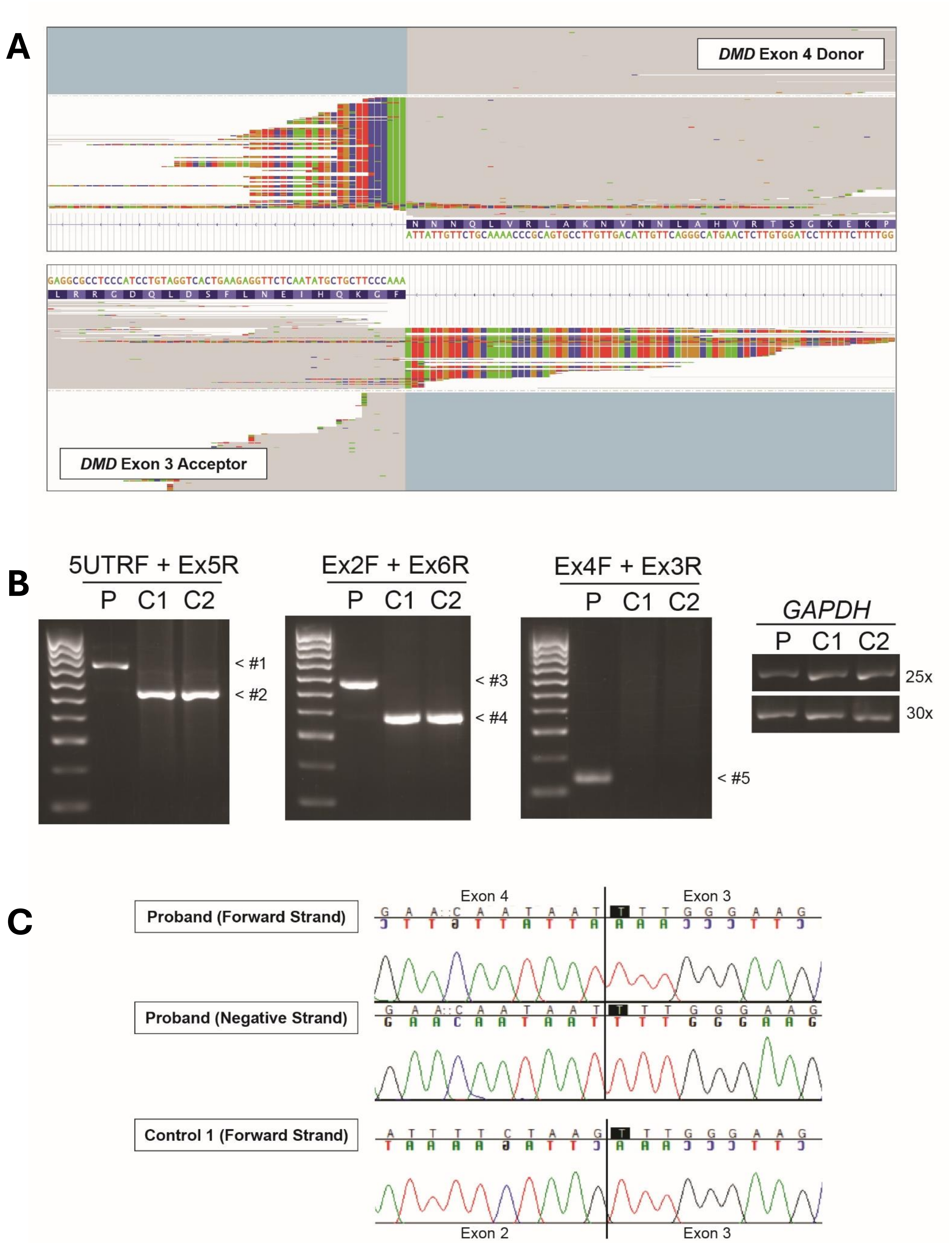

Figure S18. Analysis of a *DMD* variant using mRNA isolated from a muscle biopsy of the affected individual. (A) RNA-seq reads from skeletal muscle showing junctional reads with soft clipping in the proband which map from the exon 4 donor to the exon 3 acceptor. Reads indicate that the DMD exon 3-4 duplication in the affected individual is in-tandem. Total RNA was prepared using Illumina Stranded Total RNA Prep with Ribo-Zero Plus rRNA Depletion to yield 96,376,763 150bp paired-end reads. Reads were aligned to the GRCh38/hg38 reference genome using STAR-2.7.8a aligner. **(B)** Orthogonal reverse transcription PCR using multiple primer pairs concordantly identify the duplication of DMD Exons 3 and 4 to be in-tandem. Reverse transcription PCR of DMD cDNA derived from RNA isolated from skeletal muscle of the proband. Using two sets of primers flanking the variant we detect abnormal DMD exon 1-2-3-4-3-4-5 (Band #1) or exon 2-3-4-3-4-5-6 splicing (Band #3) only in proband. Conversely, we detect annotated DMD exon 1-2-3-4-5 (Band #2) or exon 2-3-4-5-6 (Band #4) splicing in cDNA from control muscle. Using a forward primer in exon 4 and a reverse primer in exon 3 we detect Band #5 only in the proband that Sanger sequencing confirms corresponds to aberrant exon 4-3 splicing. Amplification of GAPDH demonstrates broadly equal cDNA loading. Proband (P; male, 4 years), Control 1 (C1; male, 4 years), Control 2 (C2; male, 4 years). **(C)** Sanger sequencing chromatograms of the cDNA amplicons corresponding to the in-tandem DMD exon 3-4 duplication in the proband (exon 1-2-3-4-3-4-5; Band #1) and annotated DMD exon 1-2-3-4-5 splicing in controls (Band #2).

Figure S19

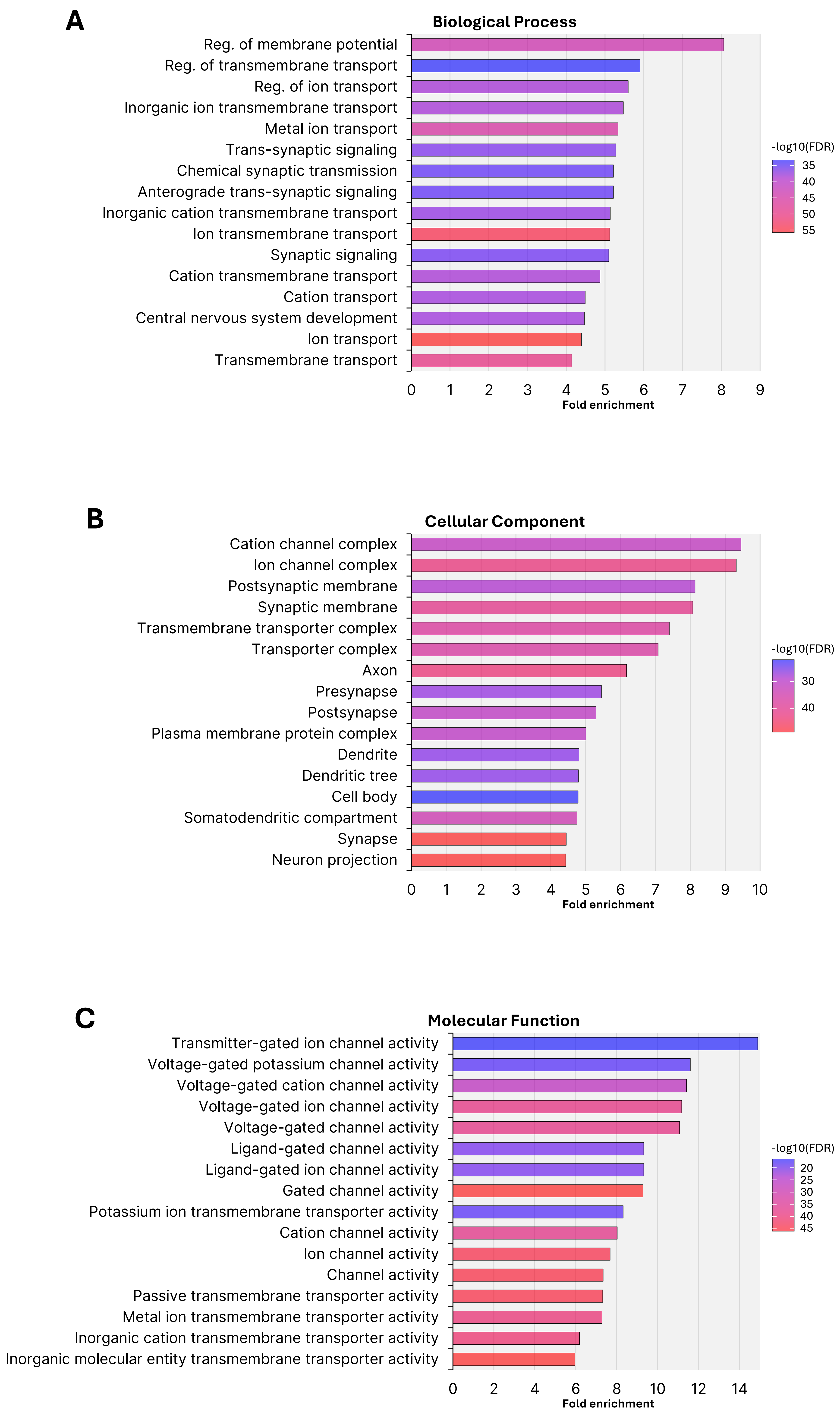

Figure S19. Gene Ontology analysis of the 516 silent neurological genes (SNGs). The SNGs were subjected to gene ontology analysis using ShinyGo v8. The highest-ranking GO terms are reported as fold enrichment and coloured based on false discovery rate (-log10FDR). **(A)** Biological Processes. **(B)** Cellular Function. **(C)** Molecular Function.

Figure S20

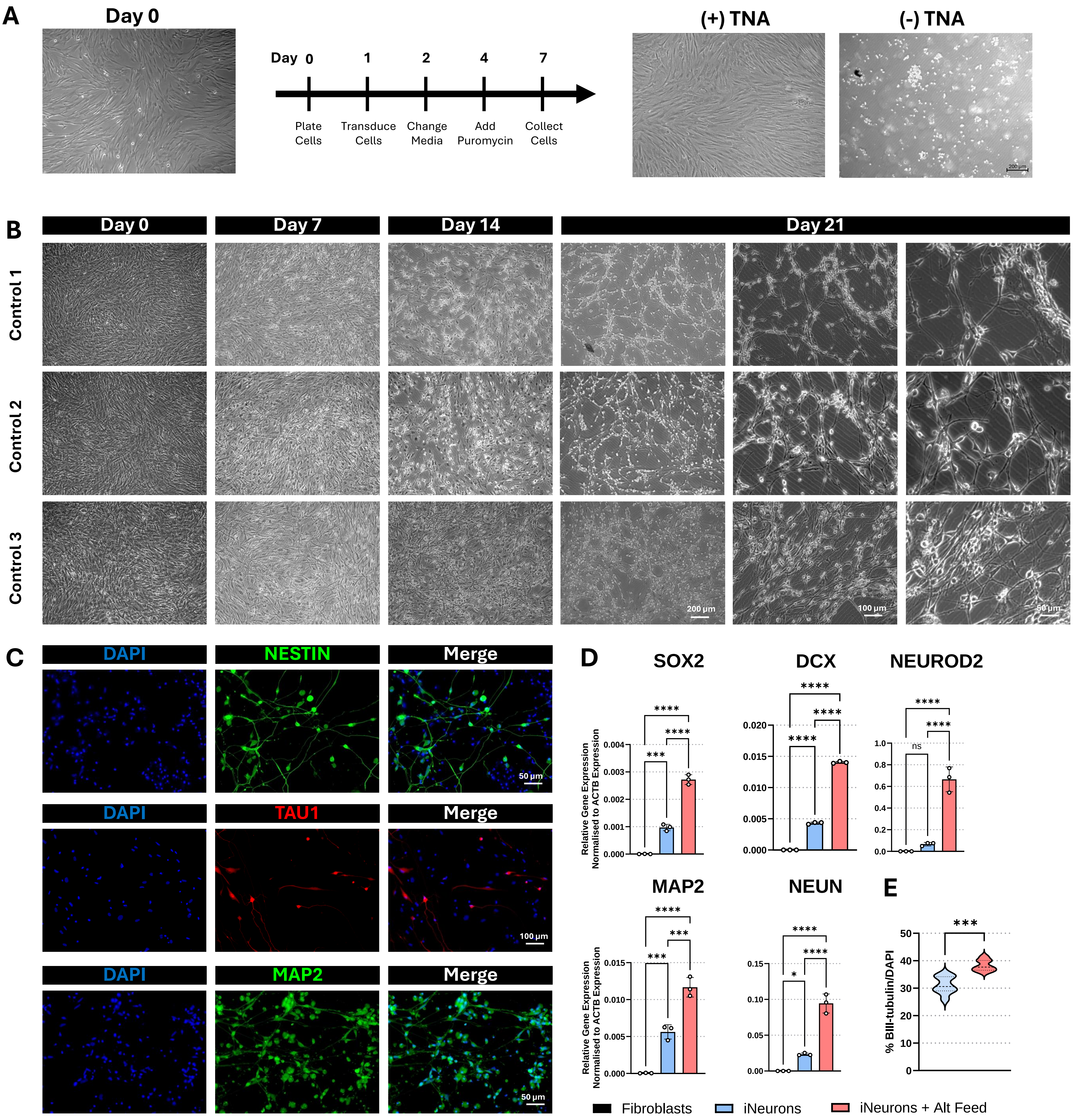

Figure S20. Transdifferentiation of HDFs to iNeurons. (A) Creation of HDF cell lines harbouring the TNA transgene. with Puromycin. HDFs are transduced with lentiviruses to deliver the TNA transgenes which confers puromycin resistance. Representative phase contrast images of confluent fibroblasts (left) and fibroblasts transduced with or without the TNA lentivirus and treated with puromycin for 72 hours (right). **(B)** Transdifferentiation of control HDFs to iNeurons**.** Representative phase contrast images of the transdifferentiation of n=3 control HDFs. **(C)** iNeurons display overt neuronal morphology and express a neuronal marker genes. Immunofluorescent imaging of day 22 control iNeurons: NESTIN (green), TAU1 (red), MAP2 (green), DAPI (blue). **(D-E)** An optimized method of transdifferentiation. HDFs transdifferentiated using either the existing protocol or an alternative protocol featuring a different media replenishment schedules (see methods; Alt Feed). **(D)** Efficiency of transdifferentiation of HDFs to iNeurons was assessed at day 22 of culture using RT-qPCR analysis of neuronal cell marker genes *SOX2*, *DCX*, *NEUROD2*, *MAP2* and *NEUN*. Expression normalised to *ACTB* expression; n=3 experiments. **(E)** Efficiency of transdifferentiation of HDFs to iNeurons was assessed at day 22 of culture using quantification of iNeurons. Cultures were fixed and immunofluorescently stained using an iNeuron marker *TUBB3* and nuclear marker DAPI. The percentage of iNeurons was calculated as TUBB3:DAPI counts. n=9 replicates. Statistical significance determined by an unpaired students T-test (E) or an ordinary one-way ANOVA with Turkey's multiple comparison test (D). Significance set as: * $p < 0.05$, ** < 0.01, *** $p < 0.001$ and **** $p<0.0001$.

Figure S21

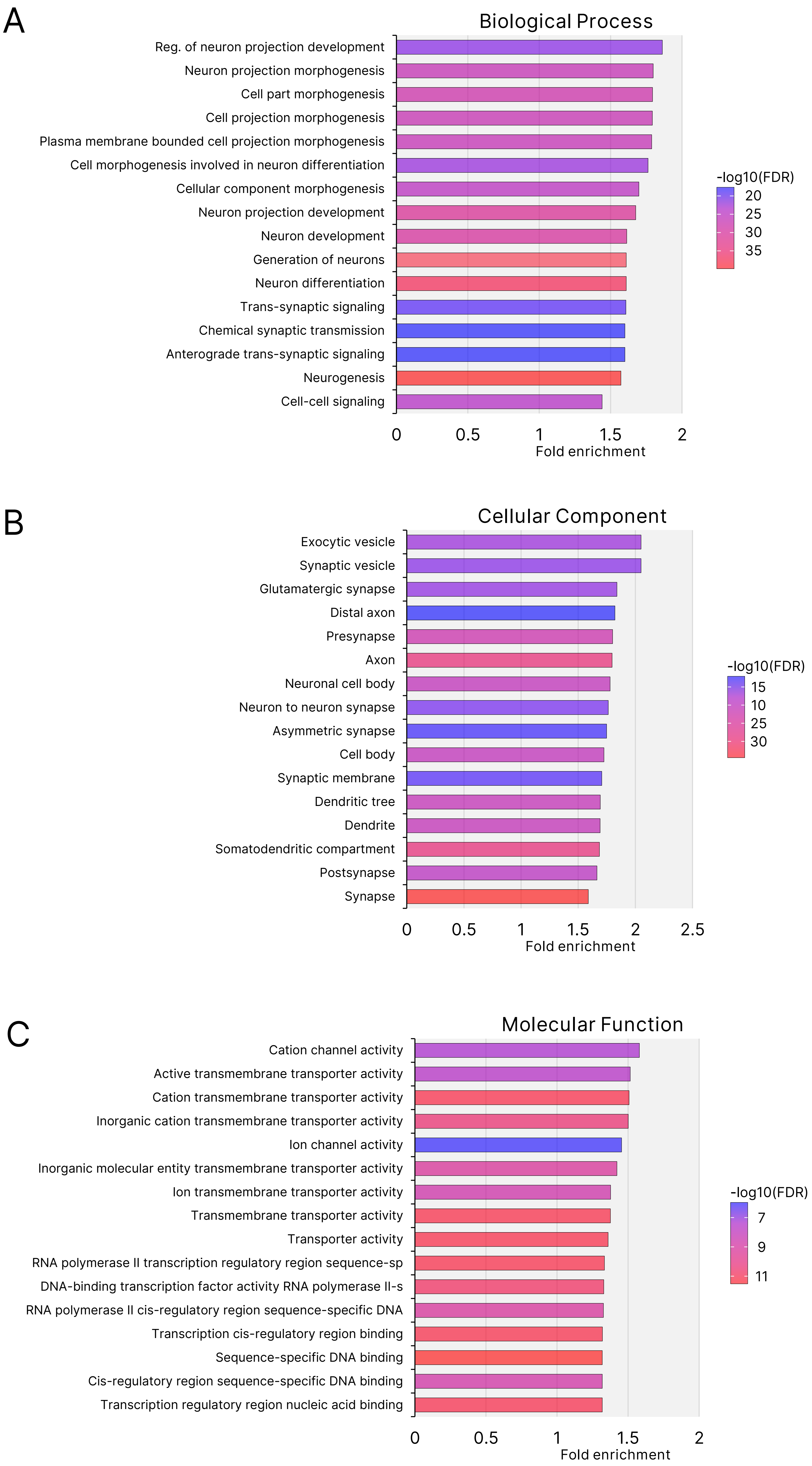

Figure S21. Gene Ontology analysis of the up-regulated genes in iNeurons after 26 days of transdifferentiation. Upregulated genes were subjected to gene ontology analysis using ShinyGo v8. The highest-ranking GO terms are reported as fold enrichment and coloured based on false discovery rate (-log10FDR). **(A)** Biological Processes. **(B)** Cellular Function. **(C)** Molecular Function.

Figure S22

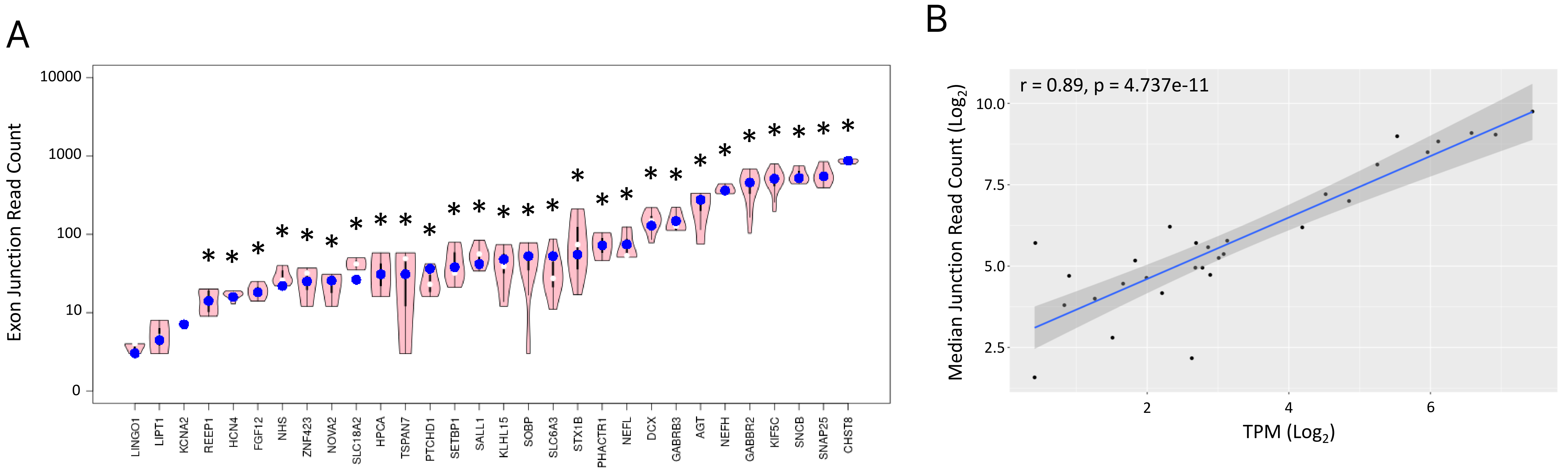

Figure S22. Number and distribution of RNA-seq reads mapping to exon-exon junctions in 30 SNGs expressed in iNeurons and their correlation to TPM. (A) A subset of 30 SNGs expressed in iNeurons were selected for analysis based on their expression levels (10 with TPM<5; 10 with TPM>5 and <10; and 10 with TPM>10). For each selected gene, the number of reads mapping across each exon-exon junction of the most predominately expressed isoform was calculated. Data is expressed as violin plot to show transcript wide distribution of read depth across junctions, with the median value highlighted in blue. Asterisks identifies the 17/20 genes with read counts satisfying the MRSD parameters used to define suitability for RNA-seq based assessment of splicing (i.e. genes with minimum 8 junction reads across 75% of junctions). **(B)** The median read depth for junctions is highly correlated with overall gene expression (as TPM) in iNeurons (Pearsons Correlation displayed).

Figure S23

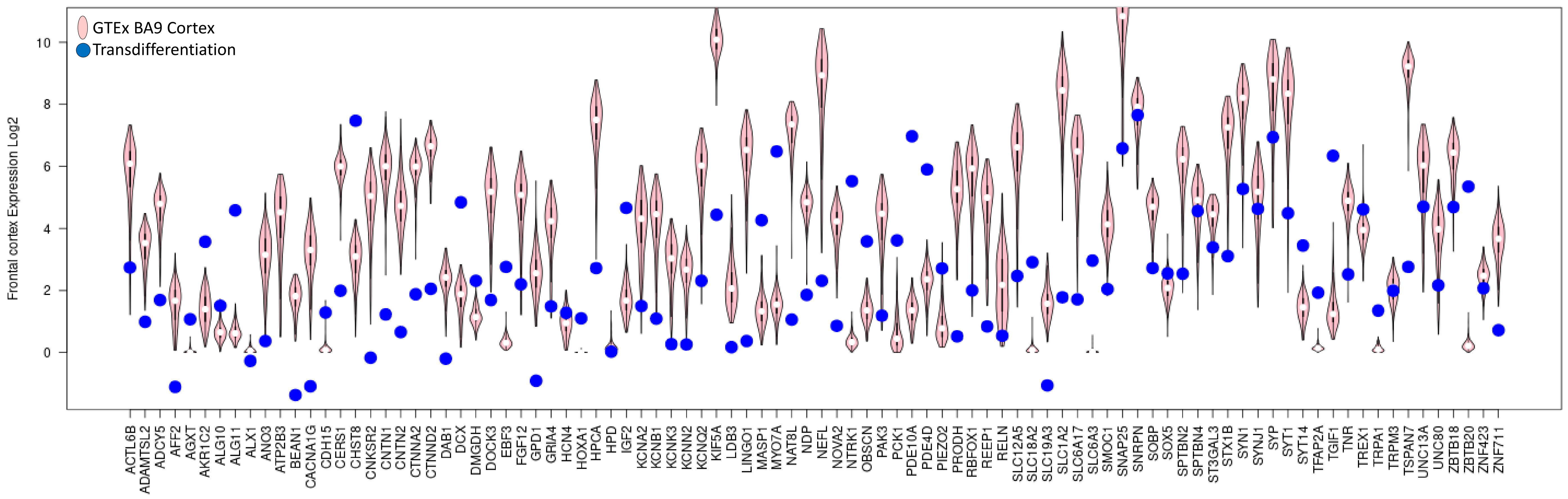

Figure S23. Comparison of the expression levels of SNGs in iNeurons with adult cortex. The expression level (TPM) of 93 of the 193 SNGs expressed in iNeurons (blue dots) is compared to range of expression levels (TPM) overserved in cortex samples from the GTEx data base Version 8 (pink violins). Note comparisons of the remaining 100 of the 193 genes are displayed in Figure 6.

Figure S24

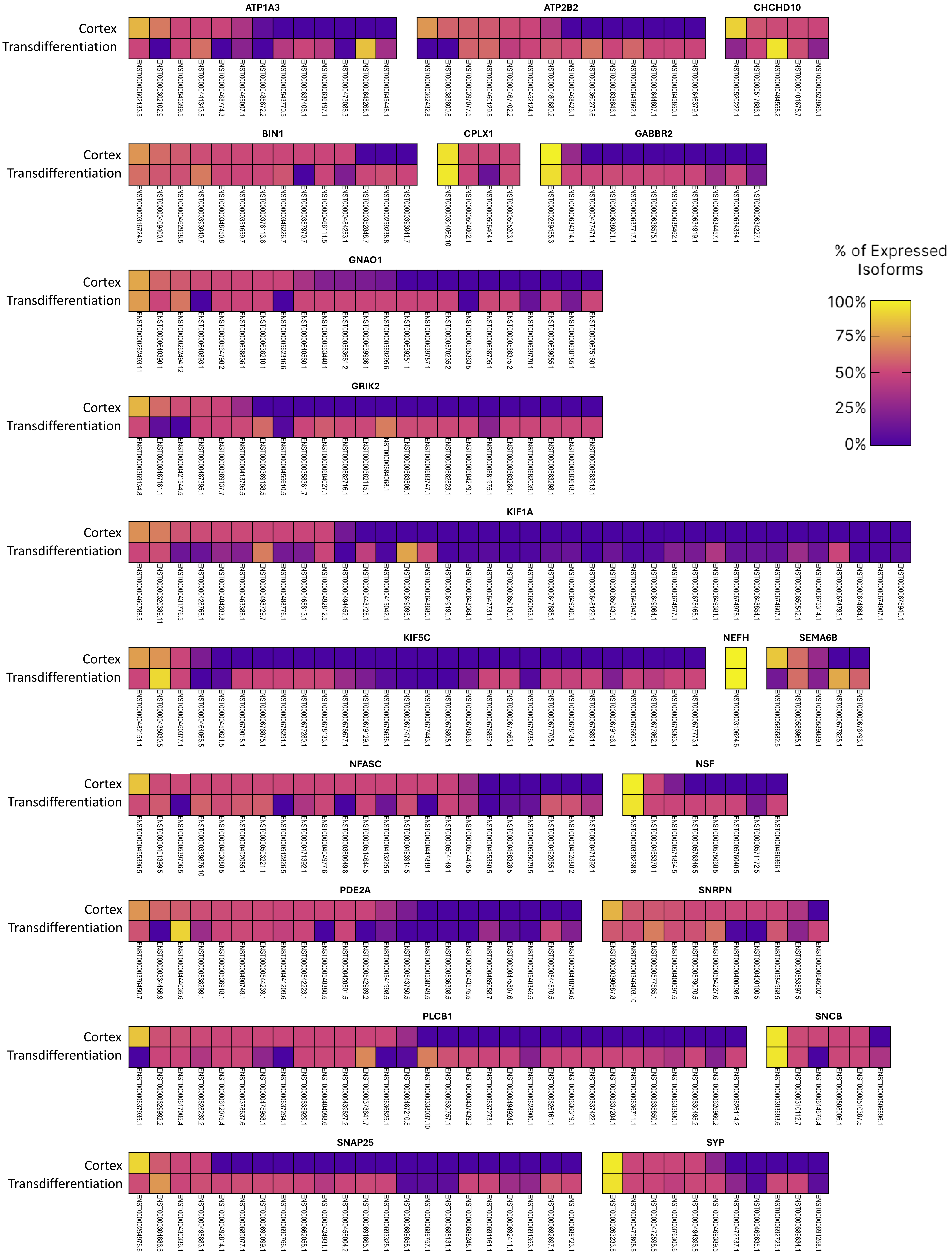

Figure S24. Comparison of the isoform diversity of 20 SNGs expressed in iNeurons with adult cortex. 20 out of the 193 SNGs that are expressed in iNeurons were randomly selected for analysis. The expression levels (Transcripts per million, TPM) of each gene isoform were quantified and the proportion of isoform expression calculated for each gene. Frontal cortex data was extracted from the GTEx data base Version 8.

Figure S25

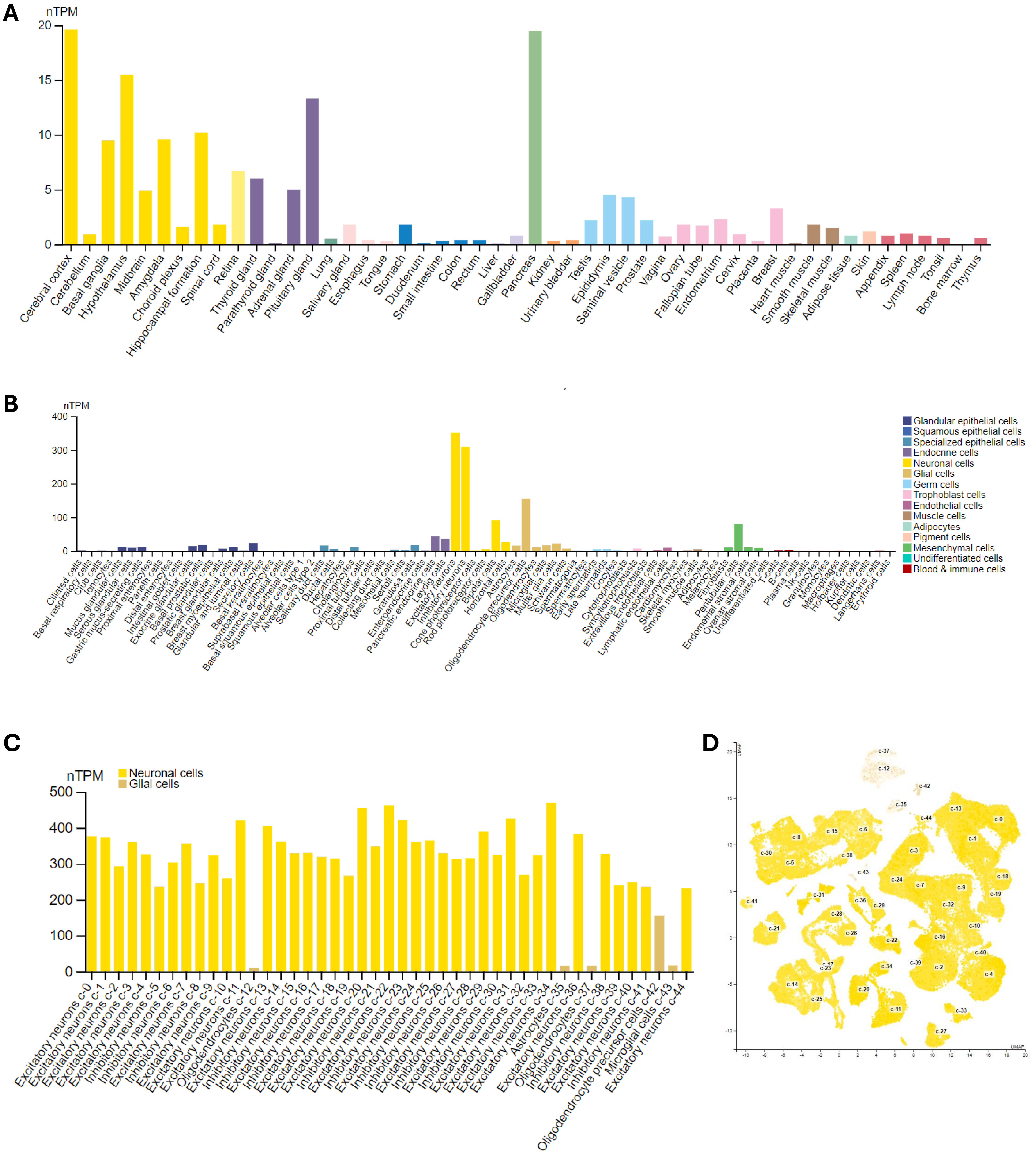

Figure S25. Expression profiles of *PAK3* in human tissues and cells. **(A)** *PAK3* mRNA expression across human tissues. *PAK3* is expressed in several brain regions, as well as pancreas, thyroid, adrenal and pituitary glands. **(B)** *PAK3* expression in cell types. *PAK3* is most highly expressed in excitatory and inhibitory neurons. **(C and D)** *PAK3* expression in brain cell types. Single cell expression data reveals that excitatory and inhibitory neurons of most subtypes express high levels of *PAK3*. All data derived from Human Protein Atlas.

Figure S26

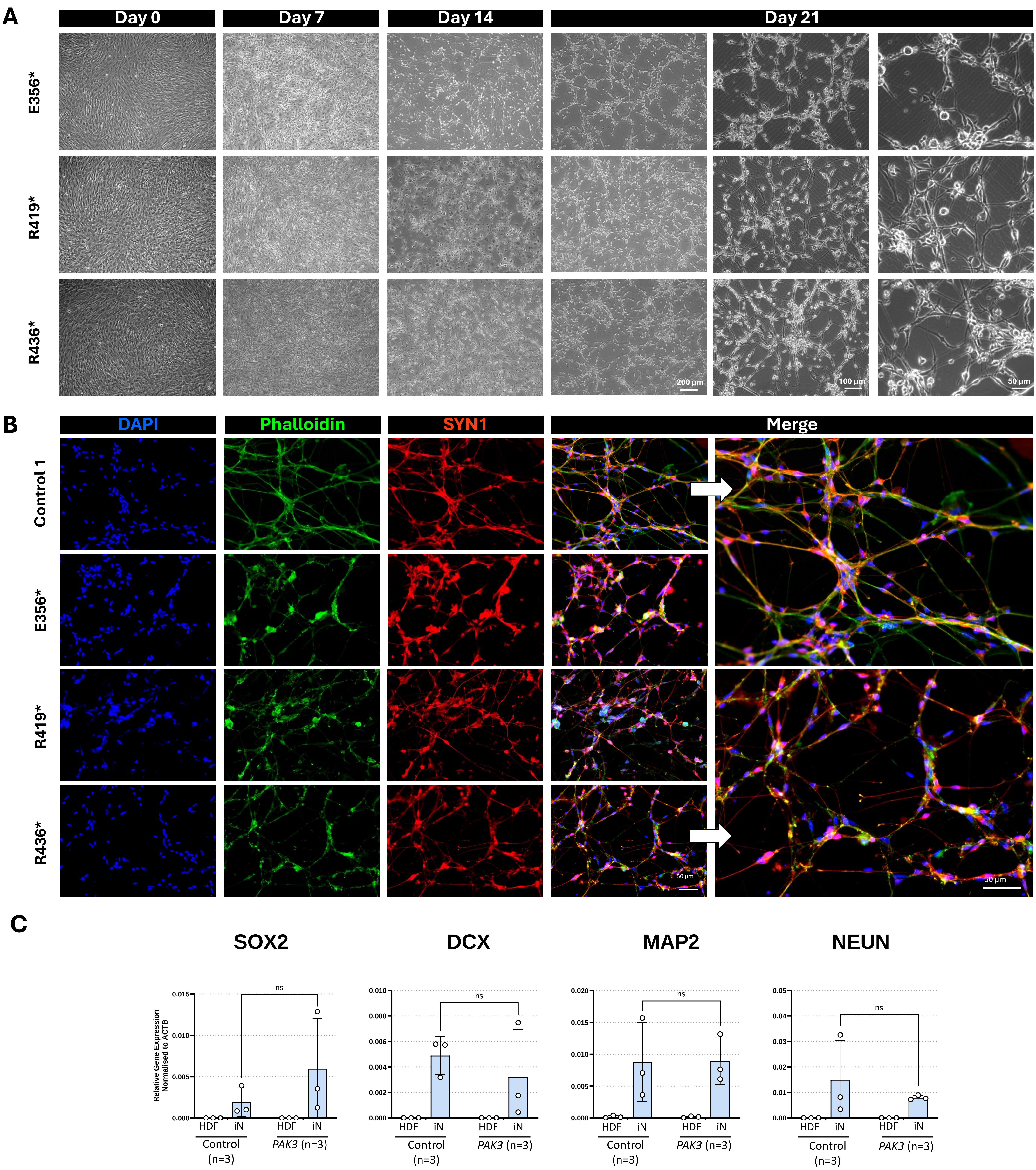

Figure S26. Transdifferentiation of HDFs derived from individuals with nonsense variants in PAK3 into iNeurons. (A) Representative phase contrast images of the transdifferentiation of HDFs harbouring variants E356*, R419* and R436* to iNeurons. (B-C) Control and PAK3 nonsense variant iNeurons display overt neuronal morphology and express neuronal cell marker genes. iNeurons were generated after 22 days of transdifferentiation and analysed. (B) Immunofluorescent detection of Phalloidin (green), SYN1 (red), and DAPI (blue). (C) qRT-PCR of *SOX2*, *DCX*, *MAP2* and *NEUN* mRNA expression in HDFs and day 22 iNeurons (iN) derived from three controls and three individuals with *PAK3* nonsense variants. Expression normalised to *ACTB* expression. Statistical analysis was determined by two-way with Šídák's multiple comparison test. Significance set as: * $p < 0.05$.

PERSYST Investigator Team

Dimitar N. Azmanov,[1] Christopher P. Barnett,[2,3,4] Simon C. Barry,[5,6,7] Gareth Baynam,[8,9,10] Samuel F. Berkovic,[11] John Christodoulou,[12,13] David J. Coman,[14,15] Sandra Cooper,[16,17,18] Mark A. Corbett,[2,19] Martin Delatycki,[12,20,21] Tracy E. Dudding,[22] Sue Fletcher,[23] Alison E. Gardner,[2,19] Jozef Gecz,[2,19,24] Megan J Higgins,[25] Michael S. Hildebrand,[11,26] Lachlan A. Jolly,[19,27] Ryan Lister,[28,29] Julie McGaughran,[25,30] Christian Pflueger,[28,29] Cathryn Poulton,[31] Tony Roscioli,[32,33,34] Ingrid Scheffer,[11,13,26,35] Hamish S. Scott,[2,36] Andrew H. Sinclair,[13,26] Amanda B. Spurdle,[37,38] Tiong Y. Tan,[12,13] Clare L. van Eyk,[2,19] and Irina Voineagu,[39]

1. Diagnostic Genomics, PathWest Laboratory Medicine, QEII Medical Centre E Block, Perth, WA, 6009, Australia.
2. Adelaide Medical School, University of Adelaide, Adelaide, SA, 5005, Australia.
3. Paediatric and Reproductive Genetics Unit, Women's and Children's Hospital, North Adelaide, SA, 5006, Australia.
4. Department of Genetics and Molecular Pathology, SA Pathology, Adelaide, SA, 5000, Australia.
5. Molecular Immunology, Robinson Research Institute, University of Adelaide, Adelaide, SA, 5000, Australia.
6. Carina Biotech, Level 2 Innovation & Collaboration Centre, UniSA Bradley Building, Adelaide, SA, 5001, Australia.
7. Department of Gastroenterology, Women's and Children's Health Network, North Adelaide, SA, 5006, Australia.
8. Telethon Kids Institute and Division of Paediatrics, Faculty of Health and Medical Sciences, University of Western Australia, Perth, WA, 6000, Australia.
9. Genetic Services of Western Australia, Western Australian Department of Health, Perth, WA, 6000, Australia.
10. Western Australian Register of Developmental Anomalies, Western Australian Department of Health, Perth, WA, 6000, Australia.
11. Epilepsy Research Centre, Department of Medicine, The University of Melbourne, Austin Health, Heidelberg, VIC, 3084, Australia.
12. Victorian Clinical Genetics Services, Murdoch Children's Research Institute, Melbourne, VIC, 3000, Australia.
13. Department of Paediatrics, University of Melbourne, The Royal Children's Hospital, Parkville, VIC, 3052, Australia.
14. Metabolic Medicine, Queensland Children's Hospital, South Brisbane, QLD, 4101, Australia.
15. School of Medicine, University of Queensland, Herston, QLD, 4006, Australia.
16. Kids Neuroscience Centre, Kids Research, Children’s Hospital at Westmead, Westmead, NSW, 2145, Australia.
17. Faculty of Medicine and Health, The University of Sydney, Sydney, NSW, 2000, Australia.
18. Children's Medical Research Institute, Westmead, NSW, 2145, Australia.
19. The Robinson Research Institute, University of Adelaide, Adelaide, SA, 5005, Australia.
20. Department of Paediatrics, Faculty of Medicine, Dentistry and Health Sciences, University of Melbourne, Melbourne, VIC, 3000, Australia.
21. Bruce Lefroy Centre, Murdoch Children's Research Institute, Parkville, VIC, 3052, Australia.
22. Hunter Genetics, Warratah, NSW, 2298, Australia.
23. Centre for Molecular Medicine and Innovative Therapeutics, Murdoch University, Perth, WA, 6000, Australia.
24. South Australian Health and Medical Research Institute, Adelaide, SA, 5000, Australia.
25. Genetic Health QLD, Royal Brisbane and Women's Hospital, Herston, QLD, 4006, Australia.
26. Murdoch Children’s Research Institute, Parkville. VIC, 3052, Australia.
27. School of Biomedicine, University of Adelaide, Adelaide, SA, 5005, Australia.
28. Harry Perkins Institute of Medical Research, Nedlands, WA, 6009, Australia.
29. Australian Research Council Centre of Excellence in Plant Energy Biology, School of Molecular Sciences, The University of Western Australia, Crawley, WA, 6009, Australia.
30. Faculty of Medicine, The University of Queensland, Saint Lucia, QLD, 4067, Australia.
31. Genetic Services of Western Australia, King Edward Memorial Hospital, Perth, WA, 6008, Australia.
32. NSW Health Pathology Randwick Genomics, Prince of Wales Hospital, Sydney, NSW, 2000, Australia.
33. Neuroscience Research Australia (NeuRA), University of New South Wales, Sydney, NSW, 2000, Australia.
34. School of Clinical Medicine, UNSW, Sydney, NSW, 2000, Australia.
35. Florey Institute of Neuroscience and Mental Health, University of Melbourne, Parkville, VIC, 3052, Australia.
36. Department of Genetics and Molecular Pathology, Centre for Cancer Biology, An SA Pathology and University of South Australia Alliance, Adelaide, SA, 5000, Australia.
37. QIMR Berghofer Medical Research Institute, Herston, QLD, 4006, Australia.
38. Faculty of Medicine, University of Queensland, Brisbane, QLD, 4000, Australia.
39. School of Biotechnology and Biomolecular Sciences, Cellular Genomics Futures Institute, and the RNA Institute, University of New South Wales, Sydney, NSW, 2052, Australia.

Acknowledgements

We would like to the acknowledge the individuals and families involved in this study. We are thankful for the contributions and support by the following: Falak Helwani (Rare Voices Australia, Melbourne, VIC, 3000, Australia), Kris Pierce (Epilepsy Foundation Australia, Surrey Hills, VIC, 3127 Australia, and Genetic Epilepsy Team Australia, Australia), Heather Renton and Maya Pinn (Syndromes Without a Name, Australia, Melbourne, VIC, 3000, Australia), Randal Grosse (South Australian Health and Medical Research Institute, Adelaide 5000, Australia), Jason Gummow (Functional Genomics South Australia, Robinson Research Institute, University of Adelaide, Adelaide, SA, 5005, Australia), Chi Lynch-Sutherland (Kids Neuroscience Centre, The Children's Hospital at Westmead, Westmead, NSW, 2145, Australia.), Tessa Mattiske (Australian Genomics, Murdoch Children's Research Institute, Melbourne, VIC, 3000, Australia) and Emma Tudini (Australian Genomics, QIMR Berghofer Medical Research Institute, Herston, QLD, 4006, Australia). L.A.J was supported by Robinson Research Institute Career Development Fellowship, and the Women's and Children's Hospital Fund - First 1000 Days Fellowship. J.G. was supported by the National Health and Medical Research Council (NHMRC) of Australia Research Fellowship (1155224). R.L. was supported by a NHMRC Investigator Grant (1178460). S.T.C. is supported by a National Health and Medical Research Council (NHMRC) of Australia Senior Investigator Grant (GNT2017952). The project is supported by the Australian Government Medical Research Future Fund (MRFF 2016447) to the PERSYST Investigator Team, led by J.G. and L.A.J, an NHMRC Ideas Grant (2029395) to L.A.J., the University of Adelaide School of Biomedicine Mature Grant to L.A.J. A part of this work was also funded by the NHMRC MRFF 'RNA for Rare Disease' research program (MRFF 2015930). Nanopore sequencing was funded by the Faculty of Health and Medical Sciences, University of Adelaide infrastructure funding to M.A.C. The research conducted at the Murdoch Children's Research Institute was supported by the Victorian Government's Operational Infrastructure Support Program. The Chair in Genomic Medicine awarded to J.C. is generously supported by The Royal Children's Hospital Foundation. J.V.B is supported by grants from the French Agence Nationale de la Recherche, ANR-21-CE17-0053 ID-GePhe-PAK and from the Fondation Jérôme Lejeune 2022-#2160. The PAK3 patients were identified through the DEFIDIAG programm (NCT04154891) funded by The French Ministry of Health in the framework of the French initiative for genomic medicine (Plan France Médecine Génomique 2025; PFMG 2025), INSERM, and the Agence Nationale de la Recherche under the "Investissements d'avenir" program (ANR-10-IAHU-01). The authors acknowledge the South Australian Genomics Centre which provided RNA-seq services. The SAGC is supported by the National Collaborative Research Infrastructure Strategy (NCRIS) via BioPlatforms Australia and by the SAGC partner institutes. We acknowledge communication of data provided by the Genotype-Tissue Expression (GTEx) Project - supported by the Common Fund of the Office of the Director of the National Institutes of Health, and by NCI, NHGRI, NHLBI, NIDA, NIMH, and NINDS (data obtained from the GTEx Portal on 08/01/24: https://GTExportal.org/home/).
